# Lineage-specific amplification and epigenetic regulation of LTR-retrotransposons contribute to the structure, evolution, and function of Fabaceae species

**DOI:** 10.1186/s12864-023-09530-y

**Published:** 2023-07-27

**Authors:** Long-Long Yang, Xin-Yu Zhang, Li-Ying Wang, Yan-Ge Li, Xiao-Ting Li, Yi Yang, Qing Su, Ning Chen, Yu-Lan Zhang, Ning Li, Chuan-Liang Deng, Shu-Fen Li, Wu-Jun Gao

**Affiliations:** College of Life Sciences, Henan Normal University, Xinxiang, 453007 P. R. China

**Keywords:** Fabaceae species, LTR-RTs, Genome evolution, LTR-RT insertion, Transcriptional activity, DNA methylation

## Abstract

**Background:**

Long terminal repeat (LTR)-retrotransposons (LTR-RTs) are ubiquitous and make up the majority of nearly all sequenced plant genomes, whereas their pivotal roles in genome evolution, gene expression regulation as well as their epigenetic regulation are still not well understood, especially in a large number of closely related species.

**Results:**

Here, we analyzed the abundance and dynamic evolution of LTR-RTs in 54 species from an economically and agronomically important family, Fabaceae, and also selected two representative species for further analysis in expression of associated genes, transcriptional activity and DNA methylation patterns of LTR-RTs. Annotation results revealed highly varied proportions of LTR-RTs in these genomes (5.1%~68.4%) and their correlation with genome size was highly positive, and they were significantly contributed to the variance in genome size through species-specific unique amplifications. Almost all of the intact LTR-RTs were inserted into the genomes 4 Mya (million years ago), and more than 50% of them were inserted in the last 0.5 million years, suggesting that recent amplifications of LTR-RTs were an important force driving genome evolution. In addition, expression levels of genes with intronic, promoter, and downstream LTR-RT insertions of *Glycine max* and *Vigna radiata*, two agronomically important crops in Fabaceae, showed that the LTR-RTs located in promoter or downstream regions suppressed associated gene expression. However, the LTR-RTs within introns promoted gene expression or had no contribution to gene expression. Additionally, shorter and younger LTR-RTs maintained higher mobility and transpositional potential. Compared with the transcriptionally silent LTR-RTs, the active elements showed significantly lower DNA methylation levels in all three contexts. The distributions of transcriptionally active and silent LTR-RT methylation varied across different lineages due to the position of LTR-RTs located or potentially epigenetic regulation.

**Conclusion:**

Lineage-specific amplification patterns were observed and higher methylation level may repress the activity of LTR-RTs, further influence evolution in Fabaceae species. This study offers valuable clues into the evolution, function, transcriptional activity and epigenetic regulation of LTR-RTs in Fabaceae genomes.

**Supplementary Information:**

The online version contains supplementary material available at 10.1186/s12864-023-09530-y.

## Background

Transposable elements (TEs) are omnipresent in plant genomes and long terminal repeat (LTR)-retrotransposons (LTR-RTs) are the most widespread components [1]. In many plant species, LTR-RTs can occupy the majority of their genomes. For example, LTR-RTs comprise over 70% and 76% of the genomes in wheat and garlic, respectively [2, 3]. A typical LTR-RT is well characterized by its structure features essential for retrotransposition, such as two highly similar LTRs and target site duplications (TSDs) flanking them [4]. A primer binding site (PBS) downstream of the 5ʹ LTR and a polypurine tract (PPT) upstream of the 3ʹ LTR are also observed [5]. Between the PBS and PPT sites is the internal region, which contains open reading frame (ORF), Gag and Pol [6, 7]. Gag, a gene that encodes structural proteins composed of the virus-like particle, and Pol encodes enzymes such as reverse transcriptase (RT) for replication, RNase H (RH) and integrase (INT), which are associated with the proliferation and integration of LTR-RTs into their host genomes [8]. According to the sequence similarity and the order of RT, RH and INT domains, LTR-RTs are primarily classified into *Copia* (GAG-PR-INT-RT-RH) and *Gypsy* (GAG-PR-RT-RH-INT) superfamilies [9]. Based on the phylogenetic analysis of the polyprotein domains, *Copia* and *Gypsy* superfamilies are generally sub-classified into nine and seven evolutionary lineages, respectively [10].

LTR-RTs transpose by employing a ‘copy-and-paste’ mechanism via an RNA intermediate, thus contributing to their considerable copies and often playing a crucial role in genome expansion. In addition, LTR-RTs insertion and deletion maintains the balance of the host genomes. The deletion is mainly result from unequal homologous recombination and illegitimate recombination, generating solo LTRs and fragmented LTR-RTs, respectively [11]. LTR-RTs are considered an evolutionary driving force that can shape the genome structure and function [12]. Moreover, the prevalence of LTR-RTs can also induce gene translocation, chromosome rearrangement [13], and regulate gene expression [14]. For instance, in apple petals, a *Gypsy* LTR-RT insertion into upstream of *MD17G1261000* allele, affecting *MYB110a* expression and then alter the flower color [15]. Hence, a comprehensive study of LTR-RTs is essential for understanding genome evolution and function. Especially, comparative analysis of LTR-RTs across some related species, such as in a family, can provide details on LTR-RT dynamics, leading to a better understanding of LTR-RTs involved in the genome evolution and function.

In most cases, LTR-RTs in the genome are transcriptionally silent, whereas under certain circumstances, such as tissue culture [16], demethylation agent [17], and abiotic stress [18], LTR-RTs can be activated. Many epigenetic mechanisms are implicated in suppressing LTR-RT transcription, including DNA methylation, histone modification, and heterochromatin formation. Among the most significant and thorough-studied epigenetic modifications is DNA methylation, which can control LTR-RT activity [19, 20]. There are three types of sequence contexts in plant genomes where cytosine methylation process is involved: CG, CHG, and CHH (H denotes A, T, or C) [21, 22]. Previous studies suggested that DNA methylation can induce the repression of LTR-RT activity, thus limits mutational effects, indicating a positive effect during the evolutionary process [23]. Furthermore, transcriptionally active LTR-RTs can further modify the epigenetic status of particular genomic regions by generating cis-regulatory elements [24]. These studies establish a link between DNA methylation and LTR-RT activation. However, few studies have focused on the intrinsic relationship between DNA methylation and the LTR-RT transcriptional activity of each lineage on a whole genome level.

The Fabaceae family, also called legume and Leguminosae, embraces near 765 genera and more than 19,500 species [25]. It comprises the third largest number of species among angiosperms. Fabaceae species are widely distributed as important crops, mainly including economically and agronomically important crop species, such as soybean (*Glycine max*) and hyacinth bean (*Lablab purpureus*). Currently, numerous Fabaceae genomes have been assembled to a relatively high level, and TEs, including LTR-RTs, from them have been identified [26–28]. Nevertheless, the characterization of LTR-RTs concentrates mainly on single or several species with different LTR-RT annotation pipelines, making the result incomparable. It remains unclear how LTR-RTs contribute to genome structure and evolution, proliferation dynamics, and methylation patterns. In this study, we systematically investigate the LTR-RTs in 54 Fabaceae species, focusing on the abundance, evolutionary dynamics, gene regulating patterns as well as the epigenetic modification of LTR-RTs.

## Results

### Phylogenetic relationship of Fabaceae species

**Fig. 1:**
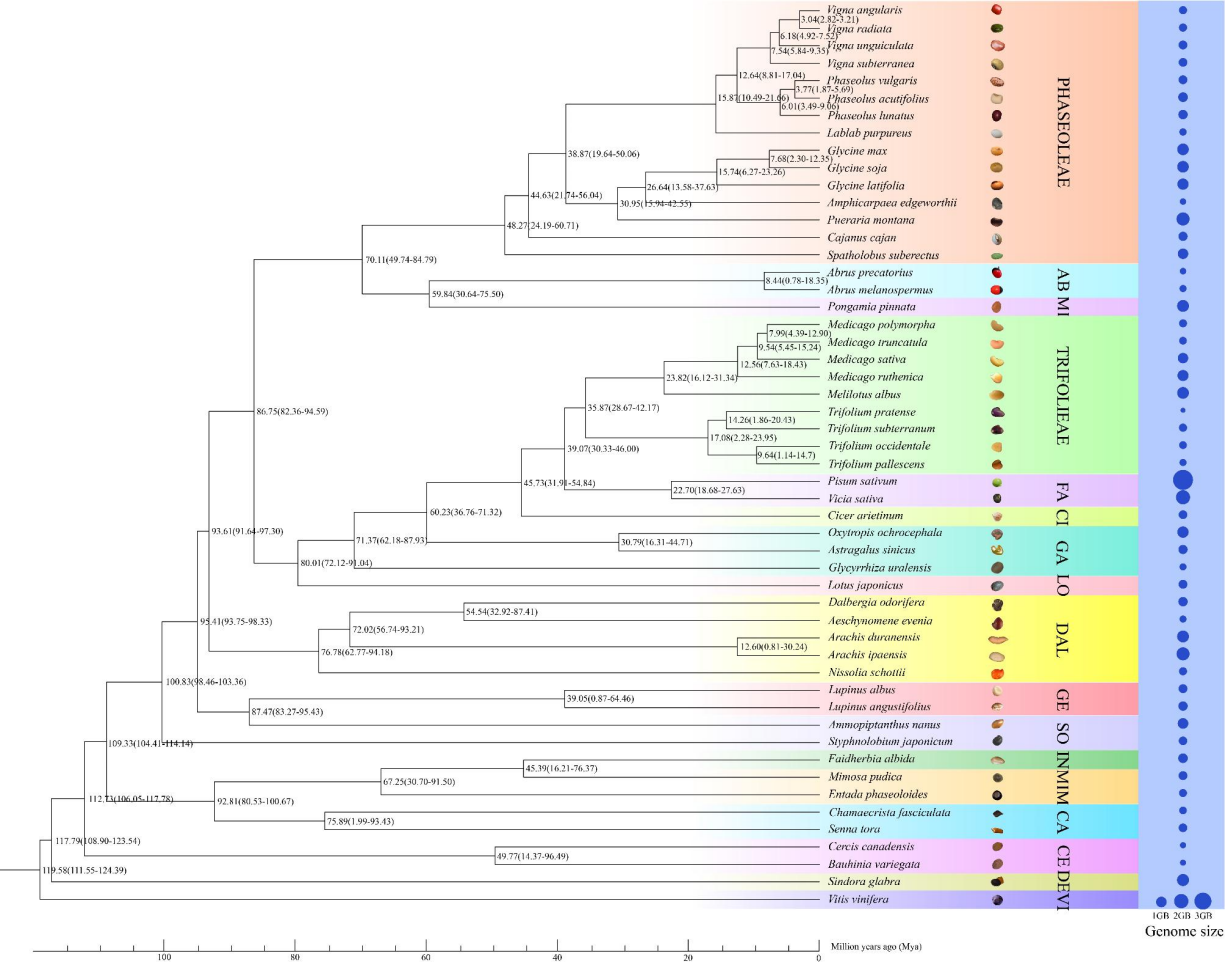
Phylogenetic tree and divergence time in 51 diploid Fabaceae species. *Vitis vinifera* was used as an outgroup. Values at branch points indicated estimates and 95% credibility intervals of divergence time (million years ago [Mya]). Names of seventeen tribes were shown on the right, and fifteen of them were abbreviated: AB, Abreae; MI, Millettieae; FA, Fabeae; CI, Cicereae; GA, Galegeae; LO, Loteae; DAL, Dalbergieae; GE, Genisteae; SO, Sophoreae; IN, Ingeae; MIM, Mimoseae; CA, Cassieae; CE, Cercideae; DE, Detarieae. Scale bar = 5 million years

In this study, we analyzed 54 Fabaceae species belonging to 36 genera from 17 tribes, and the 51 diploid species were used for phylogenetic analysis (Supplementary Table 1). The results showed that the Fabaceae species diverged from the common progenitor of the Vitaceae family approximately 119.58 million years ago (Mya) (111.55 ~ 124.39, 95% credibility interval). Then, the tribe Detarieae consisting of *Sindora glabra* and the tribe Cercideae containing *Cercis canadensis* and *Bauhinia variegata* diverged from the common progenitors approximately 117.79 (108.90 ~ 123.54) and 112.73 (106.05 ~ 117.78) Mya successively. The heterogeneous tribe Phaseoleae including eight genera (*Vigna*, *Phaseolus*, *Lablab*, *Glycine*, *Amphicarpaea*, *Pueraria*, *Cajanus*, and *Spatholobus*) appeared to form a sister clade to the tribe Abreae; the divergence time of these two clades was approximately 70.11 (49.74 ~ 84.79) Mya. Based on the phylogenetic tree, speciation event had most recently occurred between the two *Vigna* species, *V. angularis* and *V. radiata*; they diverged from the common progenitor approximately 3.04 (2.82 ~ 3.21) Mya (Fig. 1). The results were consistent with a previous study [29].

A remarkable diversity of genome sizes was observed in these Fabaceae species.

From 309 Mb in *Trifolium pratense* to 3,920 Mb in *P. sativum*, genome sizes varied more than tenfold. We performed comparative analyses across genome size in Fabaceae family, but genome contraction or expansion was species-specific and no general trend was observed in different species groups (Supplementary Table 2).

### Identification and characterization of intact LTR-RTs

**Fig. 2:**
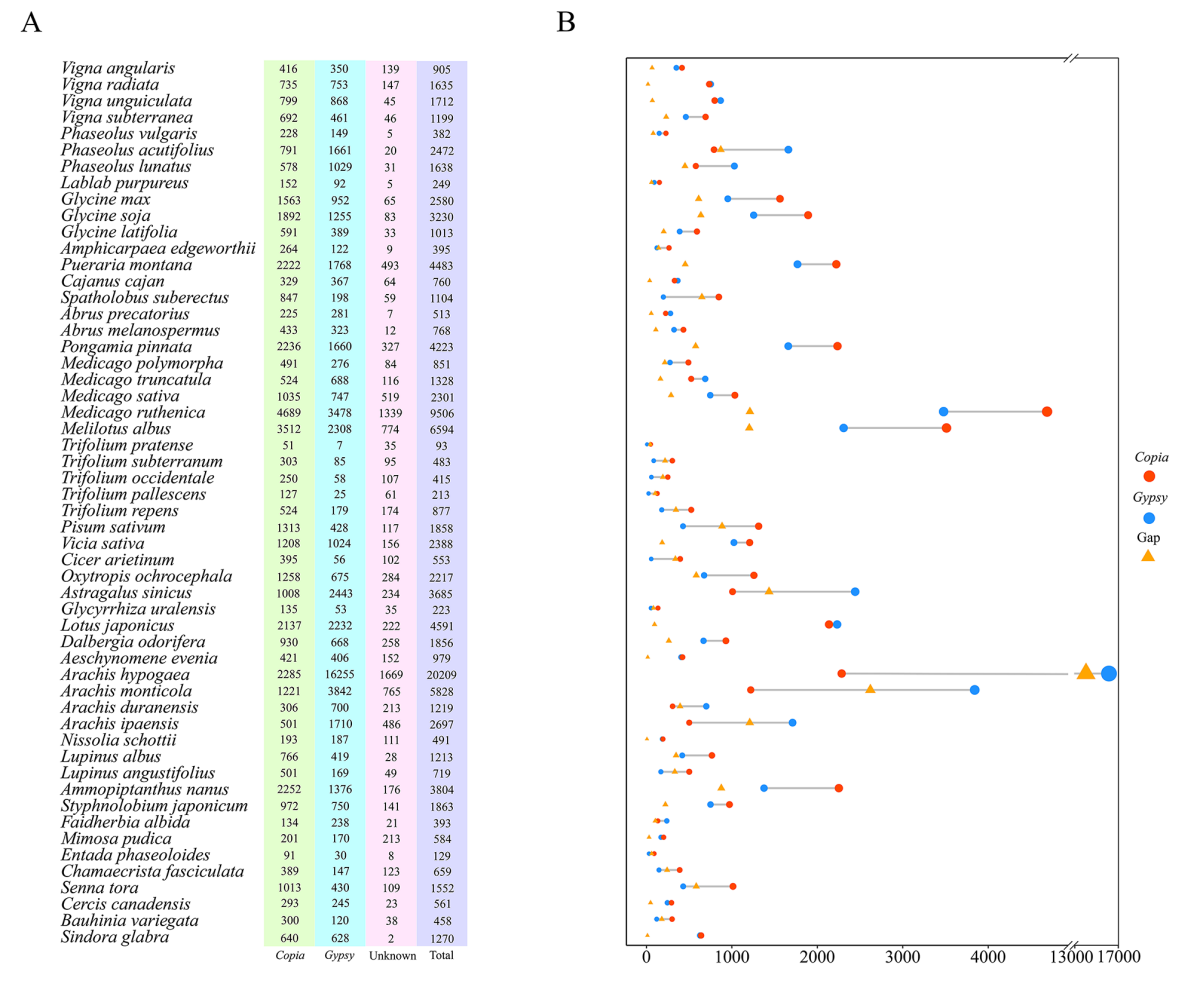
Comparative analysis of intact LTR-RT numbers in Fabaceae species. Each row represents the same species. **(A)** Specific quantities of intact LTR-RTs in Fabaceae species. **(B)***Copia* versus *Gypsy* numbers in 54 Fabaceae species. The sizes of circles and triangles increase as the values

It is well known that genome assembly quality is a critical factor for intact LTR-RT detection. Hence, we selected relatively high-quality genomes with our criteria and they were also currently the latest versions on available, with the aim of decreasing genome-quality interference as much as possible. The following results were based on these genomes. Among the 54 Fabaceae species, 113,921 intact LTR-RTs had been identified in total, including 47,362 *Copia* (41.6%) and 55,930 *Gypsy* (49.1%) elements. There were also 10,629 elements, accounting for 9.3% of the genome, defined as Unknown elements due to a lack of recognized classification patterns. The number of intact LTR-RTs in each species presented dramatic variation, ranging from 93 in *T. pratense* to 20,209 in *Arachis hypogaea* (Fig. 2A). Regarding the number distribution of *Copia* and *Gypsy*, it also revealed a great difference (Fig. 2B). The length of the LTR-RTs varied from 1,126 bp to 21,543 bp, with an average length of 7,812 bp and a standard deviation of 3,259 bp. The terminal LTRs presented a maximum of 6,908 bp and a minimum of 99 bp, with an average length of 1,086 bp (standard deviation = 791 bp). We compared the average length of LTR-RTs with their corresponding LTRs in each species. Notably, the average length of *Gypsy* elements was conspicuously greater than that of the *Copia* and Unknown elements in almost all species (50/54) (Supplementary Fig. 1A, Supplementary Table 2). Consistently, the average length of the LTRs of *Gypsy* elements was also the greatest, followed by Unknown and *Copia* elements (Supplementary Fig. 1B).

### Genome composition of LTR-RTs

The genome-wide content of LTR-RT fractions ranged from 5.1% in *T. pratense* to 68.4% in *P. sativum* based on the aforementioned intact elements (Supplementary Fig. 2A). Interestingly, the genome sizes of *T. pratense* and *P. sativum* were the minimum and maximum in this study, respectively. Similarly, the ratios between *Copia* and *Gypsy* contents differed dramatically among the 54 genomes, ranging from 0.08 in *A. hypogaea* to 4.17 in *Chamaecrista fasciculata* (Supplementary Fig. 2B). About two-thirds of these species comprised more *Gypsy* elements and fewer *Copia* elements, such as in *G. max*, *A. precatorius*, and the other 34 species, whereas in the remaining one-third of species, *Copia* elements were predominant (Supplementary Table 3).

Notably, only 1,858 intact LTR-RTs were deciphered in *P. sativum*, far less than that in *A. hypogaea* (20,209), whereas their genome sizes were comparable and both very large. However, their total LTR-RT contents were similar. This result indicated a link between genome size and their total LTR-RT contents. As was vividly demonstrated in Supplementary Fig. 3A, B, a significantly positive correlation was detected between the genome size and the entire LTR-RT fraction or genome proportion (Pearson correlation R = 0.9327 and 0.6403, respectively; p < 0.01).

### Evolutionary dynamics of LTR-RTs in Fabaceae species

**Fig. 3:**
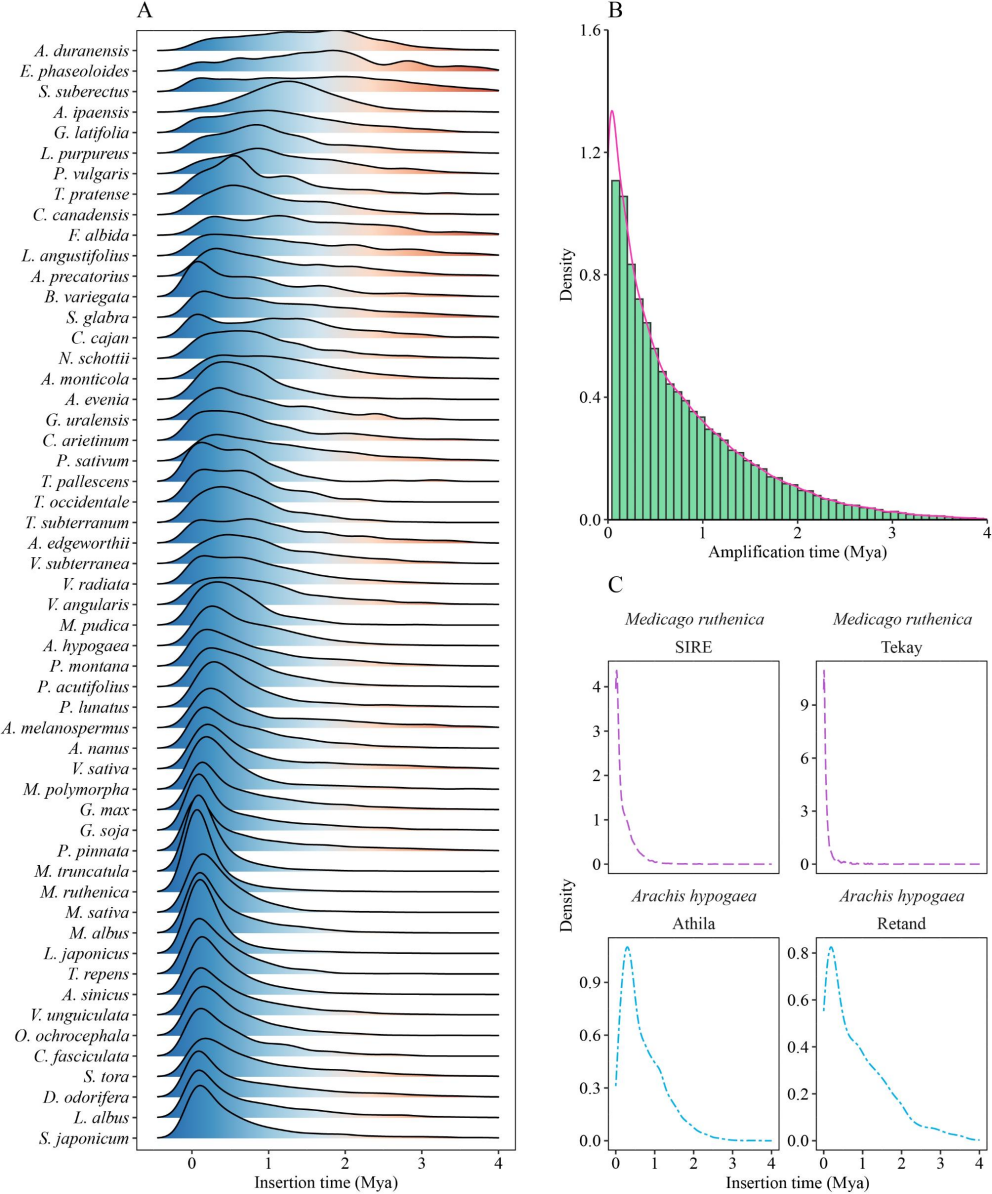
Distribution of intact LTR-RT amplification patterns **(A)** Insertion time (My) in each species. Blue indicates more recent insertions and light red exhibits more ancient insertions. **(B)** Frequency distribution of amplification time of LTR-RTs from all Fabaceae species. **(C)** Lineage-specific amplifications in *Medicago ruthenica* and *Arachis hypogaea*

In each species, we analyzed the transposition time of intact LTR-RTs based on the similarity of the two terminal LTRs, and the results revealed that the insertion events of almost all of the identified intact LTR-RTs occurred during the last 4 million years (MY). Actually, older insertions have not been considered due to the usage of a minimum of 90% identity between the two LTRs for their identification. Based on the parameter sets, the distribution of insertion time exhibited at least one round of LTR-RT burst within each genome. Several species had more complicated amplification patterns, such as two ancient rounds of bursts in *E. phaseoloides* and a long-period burst in *S. suberectus* (Fig. 3A). The bursts distribution varied among different species. Specifically, two diploid progenitors of cultivated peanut (*A. hypogaea*), *A. ipaensis* and *A. duranensis*, showed sustaining expansions, whereas *A. hypogaea* underwent a rapid burst during the last 0.5 MY. Taking consideration of all the 113,921 intact LTR-RTs in the 54 studied species, the LTR-RTs had experienced one expansion period (Fig. 3B). The expansion period was from 0.5 Mya to the present, and about 52.56% of the LTR-RTs were inserted in this stage.

**Fig. 4:**
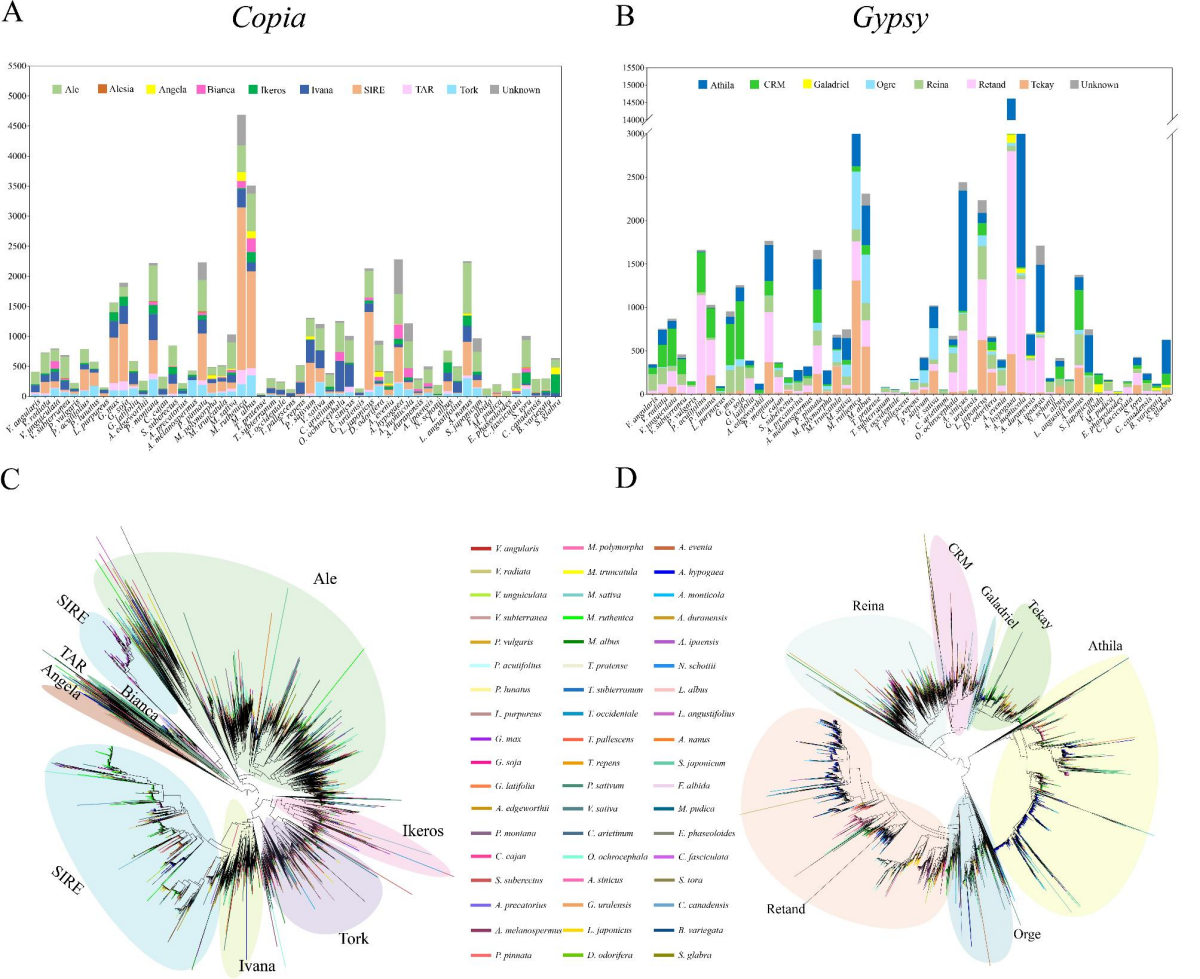
Distribution and phylogeny of different lineages of LTR-RTs in 54 Fabaceae species. **(A)** Distribution in number of *Copia* lineages identified in Fabaceae species. **(B)** Distribution in number of *Gypsy* lineages. **(C)** Phylogenetic tree of *Copia* lineages based on their RT domains. **(D)** Phylogenetic tree of *Gypsy* lineages. The trees were rooted in midpoint

We further divided the *Copia* and *Gypsy* elements into various lineages according to the sequence similarity of their coding regions to illustrate the evolutionary landscape of LTR-RTs in Fabaceae species. The results showed that *Copia* elements consisted of nine lineages (Ale, Alesia, Angela, Bianca, Ikeros, Ivana, SIRE, TAR, and Tork), whereas there were seven in *Gypsy* (Athila, CRM, Galadriel, Ogre, Reina, Retand, and Tekay) (Fig. 4A, B). The percentages of the majority of lineages showed large variation among different species, and the percentage of the SIRE lineage showed the largest variation, ranging from 70.4% in *M. ruthenica* to complete absence in *Abrus precatorius*, *T. pratense*, and so on. The sequences of non-redundant conserved RT domains were used to construct two phylogenetic trees (Fig. 4C, D). As illustrated in the evolutionary circular dendrograms, *Copia* lineages Ale and SIRE, as well as *Gyspy* lineages Athila and Retand, were more complex and heterogeneous than the other lineages in Fabaceae species.

**Fig. 5:**
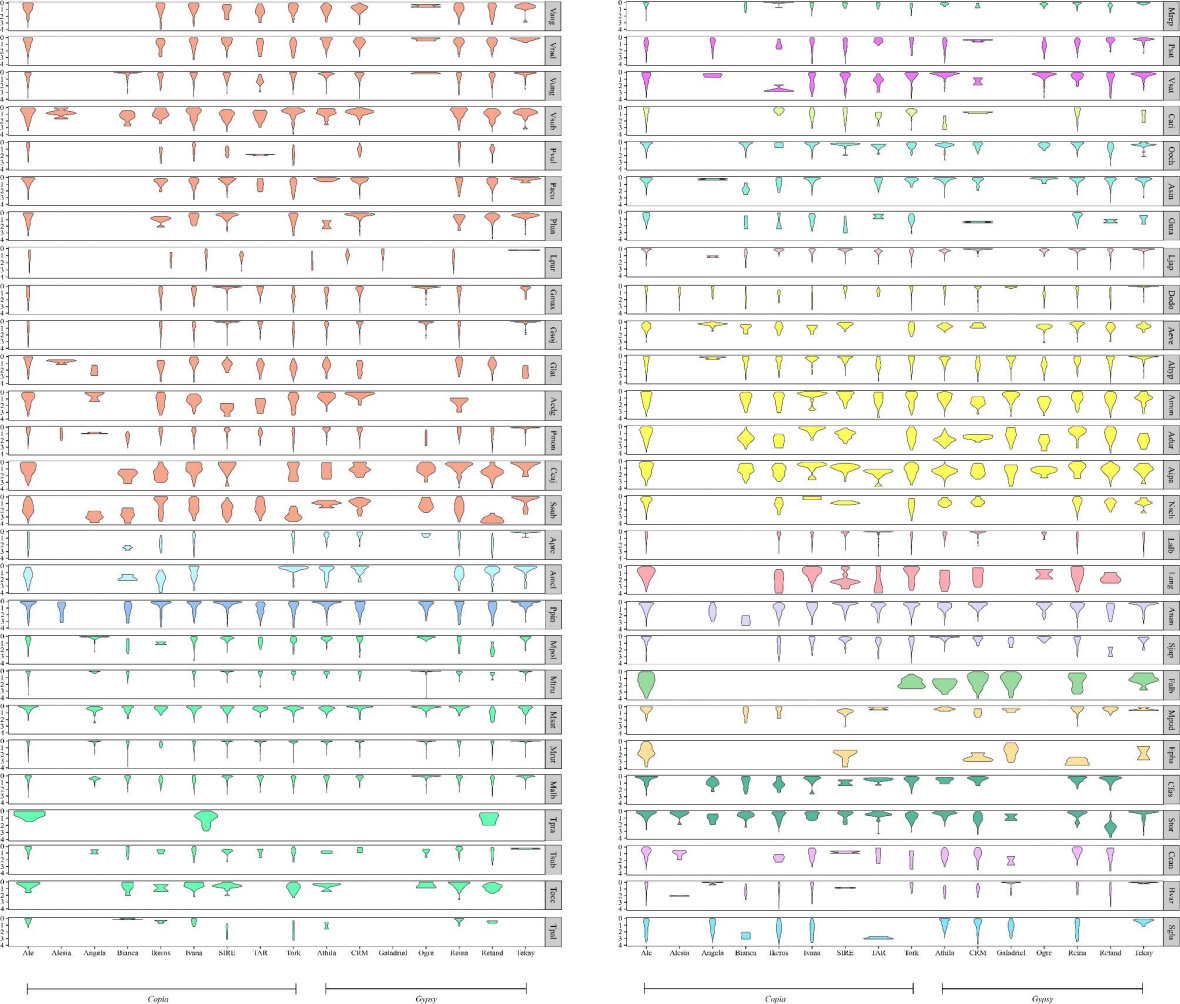
Estimation of LTR-RT insertion time of different lineages in Fabaceae species. The same color of each violin diagram represents the same tribe. The shaded parts on the right side of the violin plots are the abbreviation of every species name. For example, Gmax represents *Glycine max*

Further detail insertion time analysis on the lineage level revealed that different LTR-RT lineages in the same species always had similar burst patterns, whereas some visible discrepancies also existed. A majority of species, however, showed divergent evolutionary dynamics; some lineages exhibited short, recent expansions in one species while they showed continual moderate activity in another (Fig. 5). For example, in *M. ruthenica* with the youngest mean insertion time (0.23 MY), all lineages were inserted recently; in *E. phaseoloides* with the most ancient mean insertion time (1.65 MY), most LTR-RT lineages were inserted earlier. Combined with the lineage proportions and distributions in different species, the observations suggested that different species underwent various lineage-specific amplifications to shape their genomes. For example, the SIRE and Tekay lineages underwent one recent round of burst in *M. ruthenica*; the Athila and Retand lineages were amplified relatively complicatedly in *A. hypogaea* (Fig. 3C).

The generation of new intact LTR-RTs was balanced by deletions, which result from the formation of solo LTRs by ectopic recombination or illegitimate recombination, contributing to the genome either contracting or expanding [30]. For a detailed understanding of how this process impacted LTR-RT expansion, we calculated the ratios of solo LTRs to intact elements (S/I). The absence of correlation (Pearson’s *R* = 0.11 with *p* = 0.44) between S/I ratios and genome size indicated the removal of intact LTR-RTs was not significantly affected by genome size in the analyzed species (Supplementary Fig. 4A). On the other hand, our result revealed a weak but positive correlation between the S/I ratios and the average intact LTR-RT insertion times in each species (Pearson’s *R* = 0.3, *p* = 0.03; Supplementary Fig. 4B), which suggested that more solo LTRs would be formed over evolutionary time because the longer the LTR-RTs inserted, the higher the theoretical possibility of unequal recombination.

### Effects of LTR-RTs on related-gene expression

**Fig. 6:**
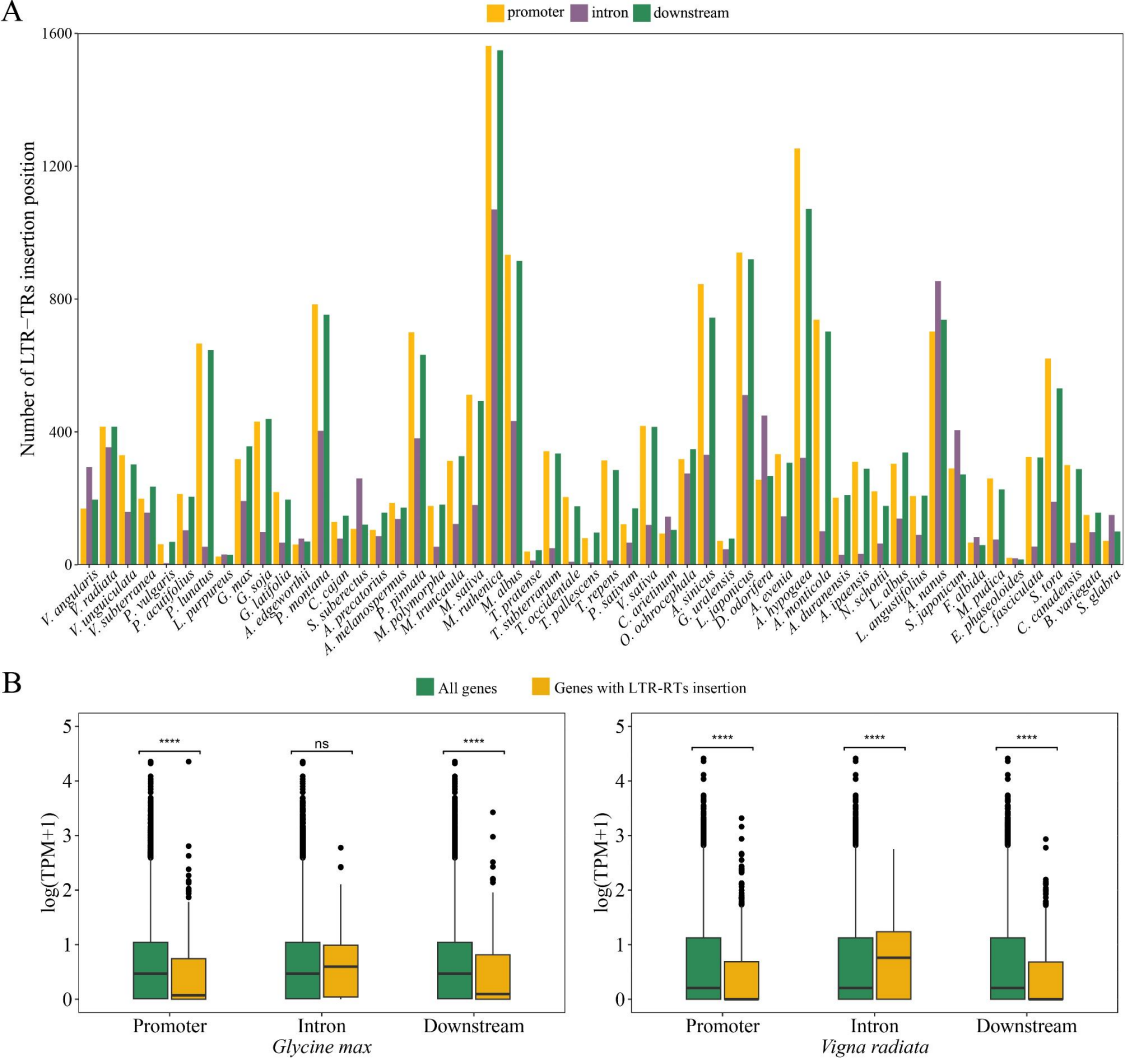
Impact of LTR-RTs on gene structure and expression. **(A)** Numbers of LTR-RT inserted locations in promoters, introns and downstream regions, respectively. **(B)** Comparison of gene expression level between genes with associated LTR-RT insertions and the whole gene set in *Glycine max* and *Vigna radiata*. p ^****^ < 0.0001; ns represents p > 0.05

In order to investigate the impact of LTR-RTs on gene structure and function, we analyzed the LTR-RT-related genes. The results exhibited that a total of 21–1562 genes had promoter LTR-RT insertions, 5–1070 genes had intron LTR-RT insertions, and 16–1549 genes had downstream LTR-RT insertions, respectively (Fig. 6A). Besides, several genes had exonic LTR-RT insertions, indicating LTR-RTs could be recruited as exons of functional genes in Fabaceae species.

To further assess the effect of LTR-RT insertion on gene expression, we performed detailed analyses of gene expression in two agronomically important species, *G. max* and *V. radiata*. T-test analysis demonstrated that genes with LTR-RT insertions in the promoter and downstream regions had significantly lower expression levels than those of the entire gene set (*p* < 0.001) in these two species. On the contrary, the expression levels of genes with intron LTR-RT insertions were significantly higher than those of the entire gene set (*p* < 0.001) in *Vigna radiata*. In *Glycine max*, no significant difference was observed between the expression levels of genes with intronic insertion and those of all genes (*p* > 0.05; Fig. 6B).

### Methylation patterns of transcriptionally active and silent LTR-RTs

To compare DNA methylation patterns of genes and LTR-RTs, we constructed methylation profiles by covering gene and LTR-RT bodies and their flanking regions in *G. max* and *V. radiata*. A much higher methylation level was detected in LTR-RTs than in genes. LTR-RT bodies were more hypermethylated than both upstream and downstream regions in CG, CHG, and CHH methylation contexts (Supplementary Fig. 5), which were consistent with the DNA methylation patterns of LTR-RTs in other plant species.

**Fig. 7:**
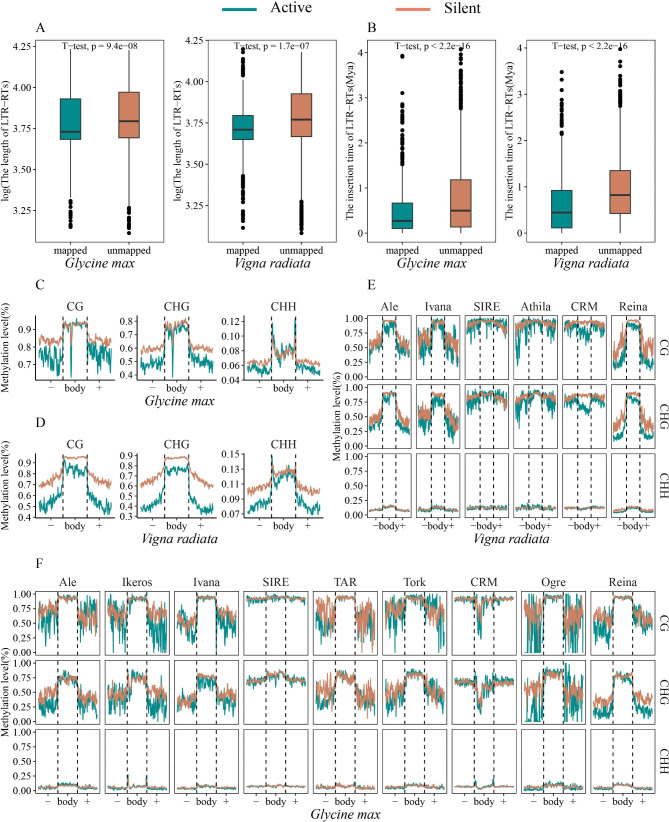
Comparison analysis in transcriptionally active LTR-RTs and silent LTR-RTs. **(A)** The differences between transcriptionally active LTR-RTs and silent LTR-RTs in terms of LTR-RT length. **(B)** The differences between transcriptionally active LTR-RTs and silent LTR-RTs in terms of LTR-RT insertion time. **(C)** DNA methylation distributions of transcriptionally active LTR-RTs and silent LTR-RTs in *Glycine max*. **(D)** DNA methylation distributions of transcriptionally active LTR-RTs and silent LTR-RTs in *Vigna radiata*. **(E)** Comparisons of DNA methylation distributions in transcriptionally active LTR-RTs lineages and silent LTR-RTs lineages in *Vigna radiata*. **(F)** Comparisons of DNA methylation distributions in transcriptionally active LTR-RTs lineages and silent LTR-RTs lineages in *Glycine max*. “-” and “+” mean upstream and downstream 2-kb regions of LTR-RTs, respectively

We further analyzed transcriptome datasets to identify transcriptionally active LTR-RTs in *G. max* and *V. radiata*. An intact LTR-RT with CPM (counts per million) values > 1 was considered to be expressed (Supplementary Table 4). With the criterion, 691 and 513 transcriptionally active intact LTR-RTs were identified in *G. max* and *V. radiata*, respectively. Compared with silent LTR-RTs, transcriptionally active LTR-RTs displayed shorter sizes and more recent insertion times, suggesting that shorter and younger LTR-RTs were prone to transpose in the genome (Fig. 7A, B). Subsequently, we compared the methylation levels in each methylation context of transcriptionally active and silent LTR-RTs in *G. max* and *V. radiata*. Both species exhibited the same pattern of apparently higher methylation levels in all contexts in silent LTR-RTs, confirming that DNA methylation played pivotal roles in repressing the activity of LTR-RTs (Fig. 7C, D).

To further detect methylation differences in LTR-RTs between transcriptionally active LTR-RTs and silent LTR-RTs, we compared the average methylation levels in each methylation context of major lineages. In general, the same lineages in the two species showed similar methylation patterns, although some differences also existed (Fig. 7E, F). Almost all lineages showed higher methylation levels of LTR-RT bodies than those of the adjacent regions, except for CRM, SIRE, and Athila lineages. The CRM lineage showed opposite results, that is, the methylation levels of LTR-RT bodies were lower than those of the flanking regions, whereas Athila and SIRE lineages displayed uniform distribution patterns in three methylation contexts in upstream 2-kb regions, body regions, and downstream 2-kb regions. To better interpret the phenomenon, we calculated TEs percentage density in the flanking regions of CRM, SIRE, and Athila versus other lineages with lower methylation levels. A considerably high TEs percentage density in the flanking regions of CRM, SIRE, and Athila was observed compared to the others (Supplementary Fig. 6). In *V. radiata*, the observations exhibited a widespread hypermethylated state in the majority of lineages in silent LTR-RTs compared to transcriptionally active LTR-RTs in CG and CHG methylation contexts (Fig. 7E). A t-test analysis of the body regions showed that the methylation levels in silent LTR-RTs were significantly higher than those in transcriptionally active LTR-RTs in all contexts except for the SIRE lineage (Supplementary Fig. 7A). In *G. max*, the most evident differences in body regions between transcriptionally active and silent LTR-RTs were observed for CG and CHG methylation in Ikeros, Ivana, TAR, and Orge lineages (Fig. 7F, Supplementary Fig. 7B). Compared with transcriptionally active LTR-RTs, most lineages of silent LTR-RTs showed increased methylation levels in upstream 2-kb regions, in particular in CG and CHG contexts. Both species had less distinct differences in methylation levels in CHH context, suggesting the universal hypermethylation state of silent LTR-RTs in CG and CHG contexts was the primary cause of deactivation of LTR-RTs.

Furthermore, we evaluated the impact of the activity of LTR-RTs on the associated genes. The results showed that the expression of genes with intronic active LTR-RT insertions was significantly higher than these with intronic silent LTR-RT insertions. No significant difference was observed between genes with transcriptionally active and silent LTR-RT insertions within their promoter or downstream regions (Supplementary Fig. 8).

## Discussion

In the evolutionary process of most plant species, LTR-RTs underwent several bursts and accumulated massive copy numbers, contributing to significant genome expansion [31]. A comprehensive comparative analysis of LTR-RTs in species of the same family or genus can enable us to systematically understand the impact of LTR-RT insertions in these related species. To date, many Fabaceae genomes have been sequenced and assembled to a high level for the sake of their agronomic or economic benefits as crops, vegetables, high-quality lumber, and medicinal herbs. These genomes make it convenient to carry out systematical comparative analyses of LTR-RT fractions among various species. In this study, we concentrated on 54 species of Fabaceae family whose genome sizes differed more than tenfold. It has been well documented that polyploidization and the amplification of TEs were the main actuation factors of the genome expansion [7]. Most of our studied species were diploid, whereas only three species (*Trifolium repens*, *A. hypogaea*, and *A. monticola*) were polyploid. For the diploid species, although they may experience polyploidization events, the events are usually ancient. For example, the investigation of WGDs (whole-genome duplication) in Fabaceae had been facilitated by widespread sequence datasets and the results reflected that ancestral Fabaceae WGD occurred ~ 55 Mya [32]. A more recent *Glycine*-specific genome duplication had subsequently occurred 5‒13 Mya [33]. The followed diploidization process can attenuate the influence of the polyploidization events on genome expansion [34]. Thus, genome expansion is less likely to be triggered by recent polyploidization events except for the three polyploid species.

As is demonstrated in this study, the total LTR-RT contents show a significant positive correlation with the genome size, indicating that the genome size variation of Fabaceae species is most possibly caused by LTR-RT proliferation. Interestingly, the LTR-RT components in *P. sativum* (~ 3.92 Gb) are the most abundant, accounting for 68.4% of the genome size, but the intact LTR-RTs are few. On the one hand, the insertion of intact LTR-RTs was more ancient, along with a higher ratio of solo LTRs to intact LTR-RTs in *P. sativum* than most other Fabaceae species. Compared with other investigated Fabaceae species, the *P. sativum* genome was evolving at a faster pace, potentially through transposon-mediated unequal recombination giving rise to ectopic double-strand break repair [35]. Thus, intact LTR-RT insertion bursts occurred anciently. On the other hand, the genome of *P. sativum* is quite complicated. Although the reference genome of pea was assembled to chromosome level, its contig N50 value was only 37.9 kb [36]. Relatively poor genome integrity made a possible factor to annotate few intact LTR-RTs. These variations lead to more solo LTRs or truncated LTRs and fewer intact LTR-RT identifications.

Almost all intact LTR-RTs were inserted into genomes in the last 4 million years based on LTR-RT insertion time estimation and Fabaceae species divergence time estimation, indicating the recent LTR-RT bursts are underway [37]. The recent RT amplification may be an indispensable force driving genome evolution. As shown in previous studies, the Ogre lineage played a pivotal role in genome evolution in Fabeae tribe [38], and the *Gypsy*-like sequences *Gorge1*, *Gorge2*, and *Gorge3* profoundly increased the genome size in *Gossypium* [39]. We also found recent lineage-specific LTR-RT bursts in *M. ruthenica* and ancient bursts in *A. hypogaea*. *Copia*/SIRE and *Gypsy*/Tekay were inserted into the genome of *M. ruthenica* during 0.5 Mya. In contrast, *Gypsy*/Athila and *Gypsy*/Retand in *A. hypogaea* exhibited relatively complicated amplification patterns. These findings revealed that different genomes displayed lineage-specific amplification of LTR-RT evolution. This variation was primarily attributed to the diverse evolutionary processes that each individual plant genome underwent [40].

In addition to affecting genome structure and evolution, there is growing evidence that LTR-RTs can significantly impact LTR-related gene expression [41]. LTR-RTs mainly insert into genomic regions such as introns, promoters, and downstream regions to regulate gene expression through various mechanisms. Comparative transcriptome analysis of two agronomically important grains, *G. max* and *V. radiata*, showed that the expression levels of genes with promoter LTR-RT insertions were significantly lower in comparison to the complete gene set. This result exhibited that LTR-RTs could influence the expression of downstream genes via several potential mechanisms, such as disruption of cis-regulatory sequences. Similarly, the expression of genes with downstream LTR-RT insertions appeared to decrease than the whole gene set. However, the expression levels of genes with intronic LTR-RT insertions exhibited diverse profiles. In accordance with a previous report, a specific grape cultivar ‘Regent’ was found to have an increased expression of the *alternate oxidase* (*Aox*) gene due to a *Copia* insertion in one intron. This insertion resulted in longer primary transcripts, leading to potentially higher levels of transcription [42]. These findings clearly indicate that different effects of LTR-RTs on gene expression are contingent upon their location.

As other autonomous transposons, LTR-RTs can transcribe, move, and facilitate adaptation in different genomic locations using the transposase under the stimulation of biotic and abiotic elicitors [43]. However, the majority of LTR-RTs in the genome still remain silent due to epigenetic suppression by the host genome. Transcriptionally active intact LTR-RTs in *G. max* and *V. radiata* are 691 (2580 in total) and 513 (1635 in total), respectively. Transcriptionally active LTR-RTs can become immobile through stochastic processes, such as the accumulation of mutations that eliminate ORFs or render translated proteins inactive, including single nucleotide changes, insertions, and deletions. LTR-RTs can also lose their mobility in the course of their own frequent transposition [44]. In our study, the length and insertion time of transcriptionally active LTR-RTs were significantly lower than those of silent LTR-RTs, suggesting that shorter and younger LTR-RTs maintained higher mobility and transpositional potential in genome.

Under normal conditions, in the absence of mutations, biotic or abiotic stress, LTR-RTs are silenced or inactivated by epigenetic silencing mechanisms, such as DNA methylation via siRNA-mediated pathways [45]. As expected, the methylation level of transcriptionally active LTR-RTs is lower than that of silent LTR-RTs in all methylation contexts in these two Fabaceae species. In addition, the extent to which transcriptionally active LTR-RTs are representative of the total element diversity present in plant genomes is not well understood, nor are the DNA methylation patterns in specific lineages. We found DNA methylation of LTR-RT upstream regions in *G. max* and LTR-RT body regions in *V. radiata* primarily regulated the activity of LTR-RTs, respectively. Lineages close to telomeres are distributed with one CG and CHG methylation level peak in LTR-RT body region and two mCG-level and mCHG-level valleys around upstream and downstream regions; the CHH methylation level exhibits a uniform distribution. In contrast, lineages near centromeres are evenly distributed in CG, CHG, and CHH methylation levels. Previous studies have shown that CRM, Athila, and SIRE lineages are preferentially located within clustered accumulation in gene-poor regions, such as heterochromatin flanking the centromeres [46–48], and therefore have higher methylation levels along LTR-RT promoter and downstream regions. Furthermore, LTR-RTs in particular and TEs are frequently inserted close to or within each other, the TEs percentage density in the flanking regions of CRM, Athila, and SIRE lineages accounted for over 80%, which could be another factor resulting in hypermethylation. In the present study, many intact LTR-RTs that were inserted into promoter regions decreased the expression of genes situated nearby in plants. The expression may be controlled by epigenetic regulation, which potentially further mediates phenotypic diversity and adaptation.

## Conclusions

The present study demonstrated the dynamic nature of LTR-RT insertions in Fabaceae species, and the systematic characterization analysis of LTR-RTs revealed their imperative role in structure, evolution, and function of Fabaceae genomes. Discrepancies in the genome composition of LTR-RTs and lineage-specific amplification patterns were observed in these species. Deletions of intact LTR-RTs and generations of solo LTRs balanced each genome. The impacts of LTR-RT insertions on related gene expression were also clear-cut. Further, comprehensive analysis based on different LTR lineages provided insights into the transcriptional activity of LTR-RTs caused by diverse DNA methylation patterns. By and large, our study in Fabaceae species has provided valuable clues in unraveling the intricate relationships between genome evolution, gene expression, transcriptional activity of LTR-RTs and DNA methylation. These clues may serve as a reference for posterity’s research on epigenetic regulation effects among various LTR lineages and potential phenotype influences.

## Methods

### Genomic datasets collection

Genomes with gene annotation files of 54 Fabaceae species were used in this study. Phylogenetic analyses were conducted using *Vitis vinifera* as an outgroup. All download links were listed in Supplementary Table 1. When multiple genome assembly versions were available for each species, we chose the higher quality assembly by synthetically considering the contig N50 value, scaffold N50 value, genome coverage, assembly level, Benchmarking Universal Single-Copy Orthologous value (BUSCO). All of the genomes confirmed the criteria of BUSCO score > 85%, contig N50 longer than 10 kb, or scaffold N50 longer than 1 Mb (Supplementary Table 5).

### Phylogeny reconstruction and estimation of divergence time

The protein sequences from 51 diploid Fabaceae species and *Vitis vinifera* were analyzed using OrthoFinder v2.5.2 [49] to identify sets of orthologous genes. Single-copy orthologs were used to construct the phylogenetic tree. We gradually aligned the single-copy orthologous protein sequences using MAFFT v7.487 [50]. PAL2NAL v14 was subsequently used to generate codon alignments using the aligned protein sequences among these species [51]. Further alignment trimming was performed using Gblocks 0.91b. Finally, we constructed a phylogenetic tree using IQ_TREE v1.6.12 [52] based on these alignments with standard model and 1,000 bootstrap replicates with *Vitis vinifera* as the outgroup and visualized using Figtree v1.4.4. The evolutionary timescale was estimated by MCMCTREE within the PAML v4.9j package [53]. Calibration points were obtained from the TimeTree database [54] for confining the nodes of the divergence time.

### LTR-retrotransposon annotation and classification

To *de novo* detect, annotate, and analyze intact LTR-RTs in 54 Fabaceae genomes, a genome-wide annotation was performed. Briefly, LTR_FINDER_parallel v1.2 [55] and LTRharvest [56] were used to predict intact LTR-RT candidates. A candidate’s two LTR regions were at least 90% identical under both parameter sets, which confine a minimum LTR length of 100 bp and a maximum LTR length of 7,000 bp. High-confidence LTR-RTs with perfect micro-structures of terminal motifs and target site duplication were identified from LTR-RT candidates using LTR_retriever v2.9.0 [57], and these LTR-RTs were regarded as intact LTR-RTs. The final identified intact LTR-RTs were mainly classified into *Copia* and *Gypsy* superfamilies. Then according to the structural features of protein domains, *Copia* and *Gypsy* superfamilies were further divided into different lineages using TEsorter v1.3 software [58] on the basis of REXdb [59]. Finally, RepeatMasker v4.1.1 was used to investigate the LTR-RT component of an individual genome, including fragmented and truncated LTR-RTs, with the TE consensuses yielded by LTR-retriever as a library.

### Insertion time estimation of intact LTR-RTs

We estimated the insertion time of each intact LTR-RT based on the nucleotide divergence (K) between 5ʹ and 3ʹ LTRs by using the LTR_retriever. Synonymous substitution rate (r) of 1.3 × 10^− 8^ mutations per site per year was used for calculations in this study [60]. The estimated insertion time (T) was measured with the formula *T = k/2r*.

### Analysis of the phylogeny of intact LTR-RTs

In order to construct phylogenetic trees of *Copia* and *Gypsy* elements, the RT protein sequences were extracted, and CD-hit v4.8.1 [61] was used to remove redundant sequences with parameters “-c 1 -aL 0.9 -AL 10 -aS 1 -AS 1 -d 0”. RT sequences were then aligned globally using muscle version 3.8.1551 [62]. The *Copia* and *Gypsy* phylogenetic trees were generated by FastTree [63] and further edited using the iTOL online tool [64].

### Solo LTR detection

In the annotation output of RepeatMasker, some LTR-related regions exactly covered LTR consensus sequences (identity > 80%) but with no internal sequences flanking them. We critically detected the upstream and downstream 6 bp regions of each LTR-related region; if a 4–6 bp TSD was presented, the LTR-related sequence would be regarded as a solo LTR [65]. Custom R scripts (available on request) were used to implement this process.

### Analysis of LTR‑RT associated gene expression

Genes were regarded as associated with LTR-RTs if one LTR-RT was inserted into their introns, exons, promoters (5 kb flanking sequences from transcription start site [TSS]), or downstream 5 kb region (5 kb flanking sequences from transcription termination site [TTS]). Accordingly, we categorized genes into four groups: one with intronic LTR-RT insertions, one with exonic LTR-RT insertions, one with promoter LTR-RT insertions, and one with downstream LTR-RT insertions.

RNA sequence reads of *G. max* and *V. radiata* were downloaded from the NCBI SRA with the accession numbers SRR12494493, SRR12494493, SRR16477676, and SRR16477677 (two replications per species). The reads were aligned to reference genomes using HISAT2 v2.2.1 [66] with default parameters. FeatureCounts v2.0.1 [67] was used for gene quantification with TPM (transcripts per million).

### Identification of transcriptionally active LTR-RTs and global expression

Transcriptionally active LTR-RTs were analyzed by mapping the aforementioned RNA-seq reads to corresponding reference genomes. HISAT2 was used with parameters “--all --no-mixed” for the sake of suppressing unpaired alignments for paired reads, retaining all paired-end counts to improve accuracy. According to the existence of non-coding regions such as PBS, PPT, and flanking LTR sequences, the expression levels of LTR-RTs were quantified with CPM using featureCounts.

### Analysis of methylation levels of LTR-RTs

Methylome data of *G. max* and *V. radiata* were obtained from the NCBI SRA with accession numbers SRR12494495 and SRR16477683, respectively. Fastp v0.23.2 [68] was used to trim low-quality reads. Maps of clean reads to reference genomes were performed using Bismark v0.24.0 [69]. Based on the methylation detection results of Bismark, genes, LTR-RTs, or other genomic regions can be calculated in terms of methylation levels using BatMeth2 [70]. Graphics summarizing the analyzed results were drawn using ggplot2 in R v4.1.3 software.

## Electronic supplementary material

Below is the link to the electronic supplementary material.

Supplementary Material 1

## Data Availability

All data generated or analyzed during this study are included in this published article and its supplementary information files.
