# Supplementary material for: Lineage-specific amplification and epigenetic regulation of LTR-retrotransposons contribute to the structure, evolution, and function of Fabaceae species
Source: BMC Genomics. 2023 Jul 27;24:423. doi: 10.1186/s12864-023-09530-y (PMC10373317; doi:10.1186/s12864-023-09530-y)
Supplement: Supplementary file 1 — Supplementary Material 1 [file 12864_2023_9530_MOESM1_ESM.pdf]

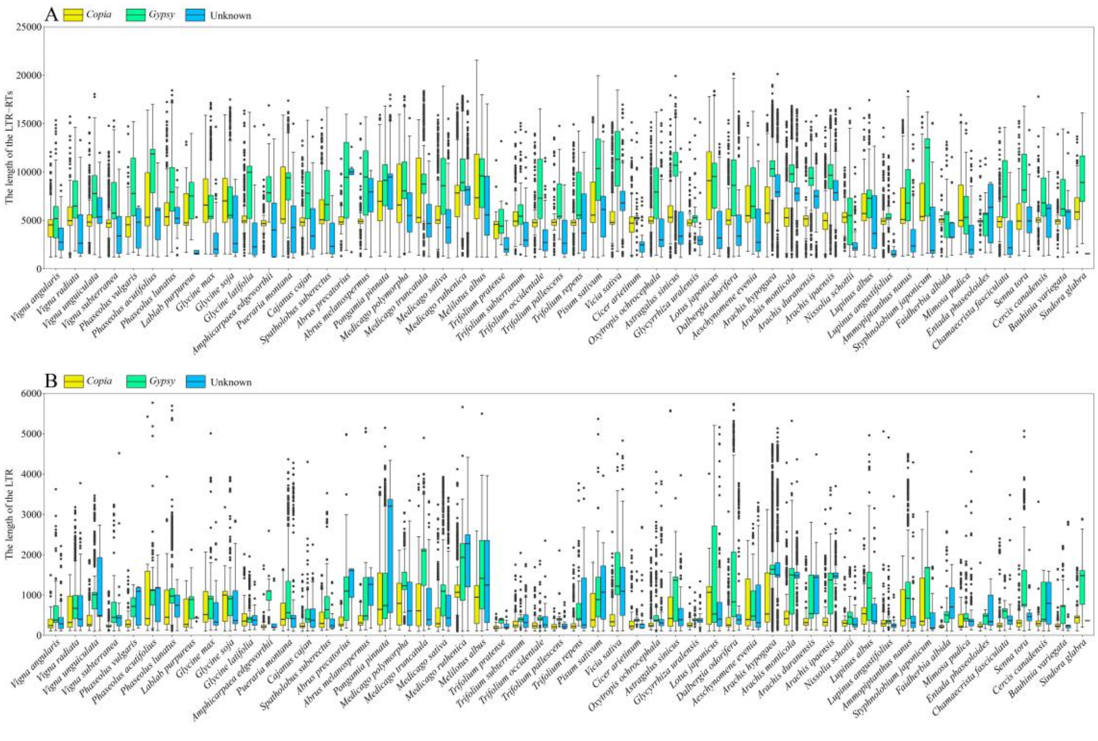

Supplemental Fig. 1. Length of intact LTR-RTs (A) and their corresponding LTRs (B).

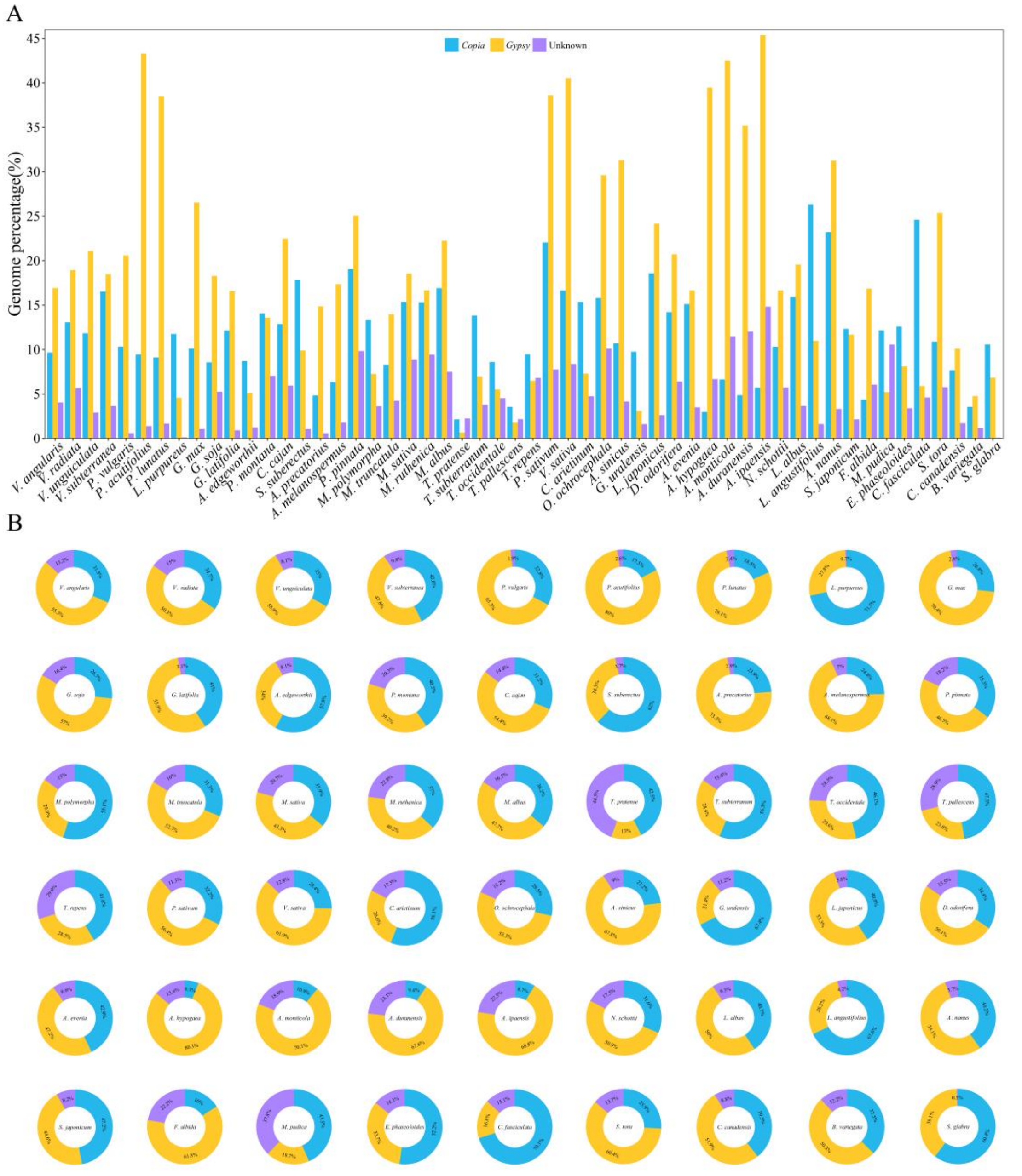

Supplemental Fig. 2. Genomic content (A) and Proportion (B) of LTR-RT fractions in *Copia*, *Gypsy* and Unknown elements in 54 Fabaceae species.

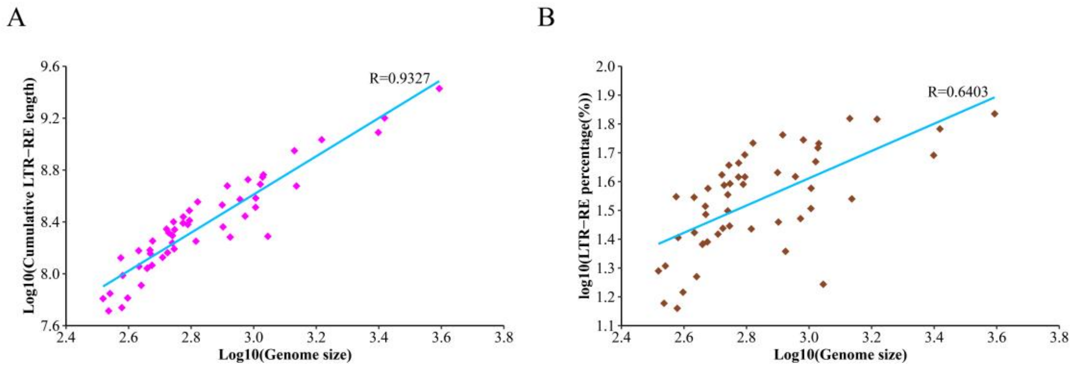

Supplemental Fig. 3. Correlation between genome size and LTR-RT fractions in Fabaceae species. (A) Genome size versus the total length of LTR-RT fraction including intact and fractionated elements; (B) Genome size versus the percentage of genomes occupied by LTR-RT fraction.

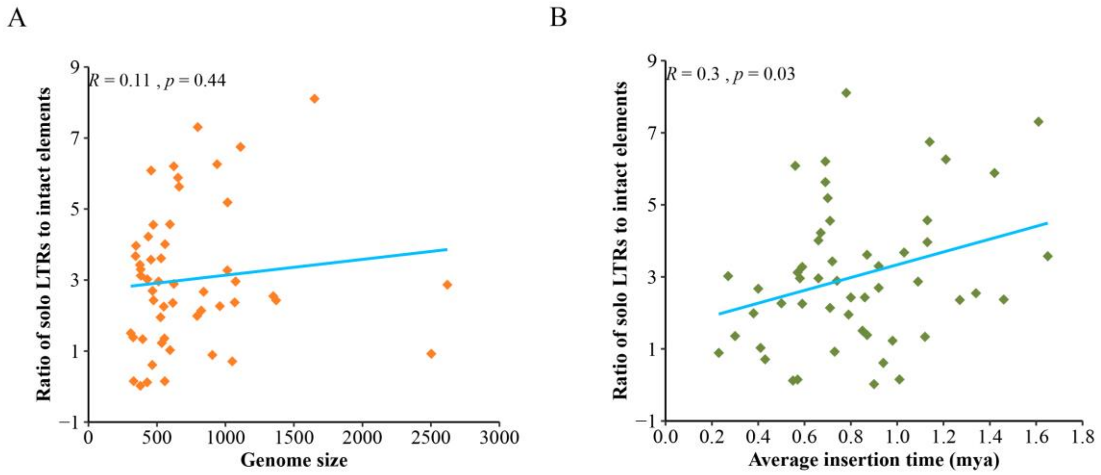

Supplemental Fig. 4. Genetic factors associated with solo LTRs formation. (A) Ratios of solo LTRs to intact elements (S/I) versus genome size. (B) S/I versus average insertion time (mya).

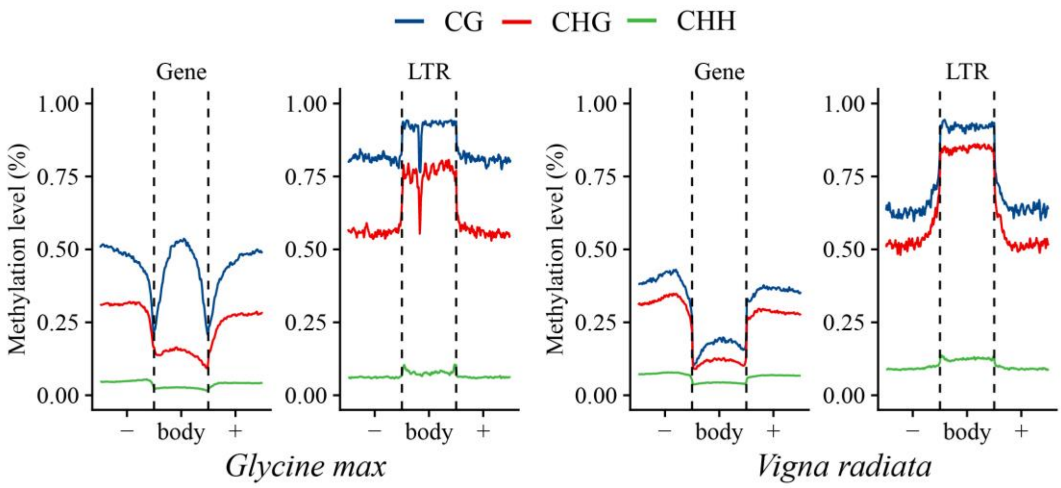

Supplemental Fig. 5. Landscape of DNA methylation in genes and LTR-RTs. Comparison of average methylation level of CG, CHG, CHH context distribution over genes and LTR-RTs.

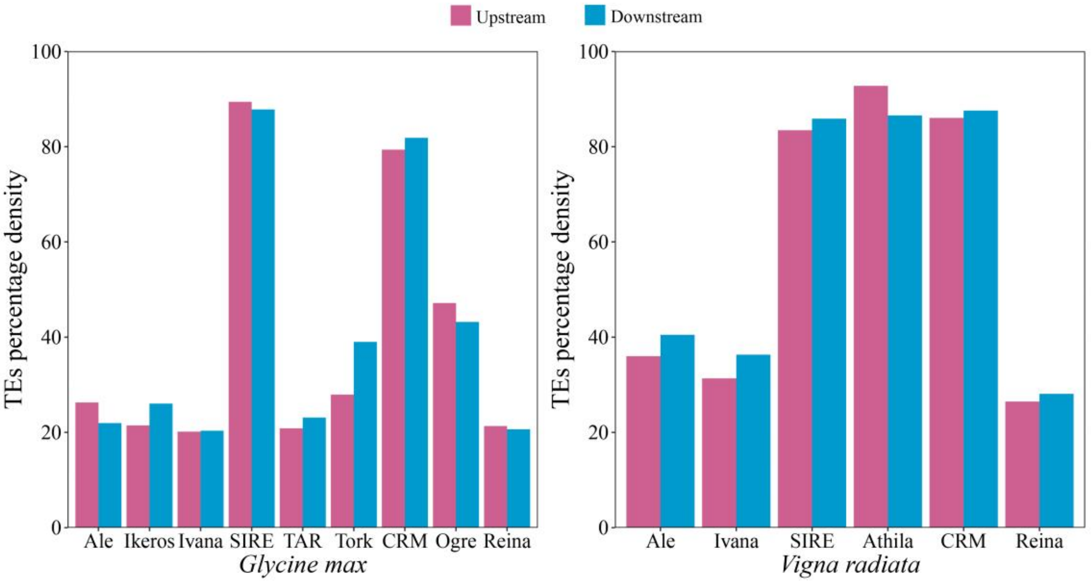

Supplemental Fig. 6. TEs percentage density in the Upstream and downstream regions of various lineages in *Glycine max* and *Vigna radiata*.

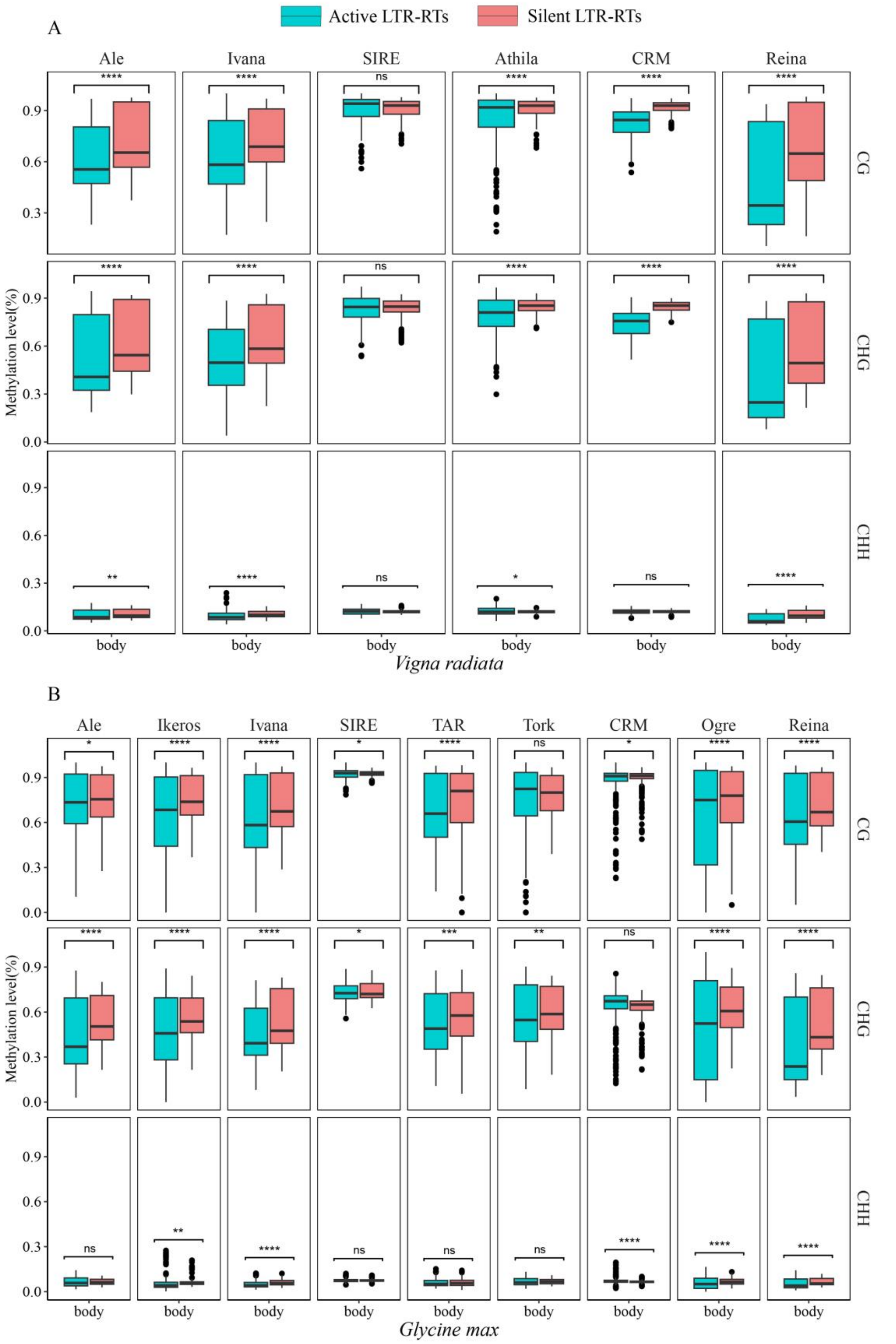

Supplemental Fig. 7. T-test analysis of the body regions of the methylation levels in transcriptionally active LTR-RTs verse silent LTR-RTs in all contexts in *Vigna radiata*

(A) and *Glycine max* (B).

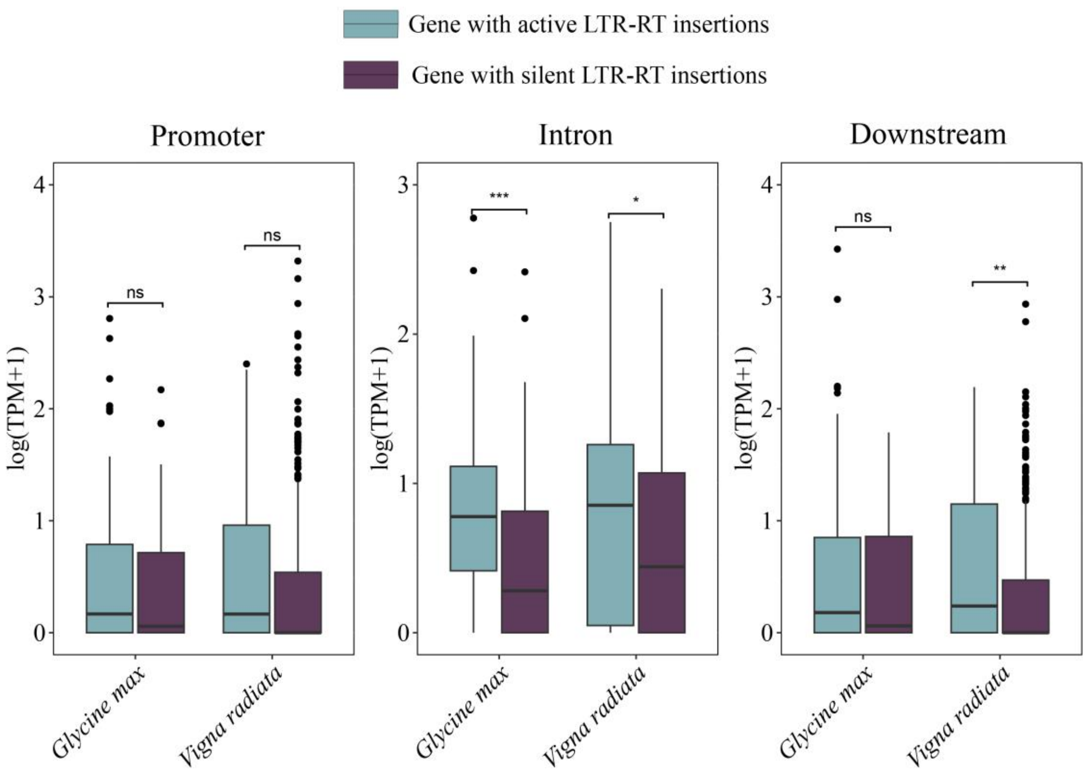

Supplemental Fig. 8. Comparisons of gene expression between transcriptionally active and silent LTR-RT insertions into promoter, intron and downstream regions.

Supplemental Table 1. Detailed information of the genomes of 54 Fabaceae species and 1 related Vitaceae species.

Species	Genus	Tribe	Family	Estimated genome size (Mb)	Assembled genome size (Mb)	Chromosome number	Ploidy	Genome download linkage
Vigna angularis	*Vigna*	*Phaseoleae*	*Fabaceae*	542	467.3	22	diploid	https://www.ncbi.nlm.nih.gov/genome/11109?genome_assembly_id=244761
Vigna radiata	*Vigna*	*Phaseoleae*	*Fabaceae*	479.4	475.2	22	diploid	https://doi.org/10.6084/m9.figshare.19583446
Vigna unguiculata	*Vigna*	*Phaseoleae*	*Fabaceae*	590	549.8	22	diploid	https://www.ncbi.nlm.nih.gov/genome/11030?genome_assembly_id=443132
Vigna subterranea	*Vigna*	*Phaseoleae*	*Fabaceae*	550	535.1	22	diploid	https://bioinformatics.psb.ugent.be/gdb/aocc/vigsu/
Phaseolus vulgaris	*Phaseolus*	*Phaseoleae*	*Fabaceae*	-	549.6	22	diploid	https://www.ncbi.nlm.nih.gov/genome/380?genome_assembly_id=48590
Phaseolus acutifolius	*Phaseolus*	*Phaseoleae*	*Fabaceae*	676	661.9	22	diploid	https://data.jgi.doe.gov/refine-download/phytozome?organism=PacutifoliusWLD&expanded=581
Phaseolus lunatus	*Phaseolus*	*Phaseoleae*	*Fabaceae*	686	623	22	diploid	https://doi.org/10.25387/g3.14398910
Lablab purpureus	*Lablab*	*Phaseoleae*	*Fabaceae*	423	395.5	22	diploid	https://bioinformatics.psb.ugent.be/gdb/aocc/labpu/
Glycine max	*Glycine*	*Phaseoleae*	*Fabaceae*	1016.3	1015.4	40	diploid	https://cegresources.icrisat.org/data_public/legumepedia_data/Glycine_max/
Glycine soja	*Glycine*	*Phaseoleae*	*Fabaceae*	-	1013.8	40	diploid	https://www.ncbi.nlm.nih.gov/genome/13239?genome_assembly_id=453634
Glycine latifolia	*Glycine*	*Phaseoleae*	*Fabaceae*	1130	939	40	diploid	https://drive.google.com/drive/folders/1_DJqYM9jnSN2Pfru_utgYudO-qBNXcNe?usp=sharing

Amphicarpaea edgeworthii	*Amphicarpaea*	*Phaseoleae*	*Fabaceae*	360.9	343.8	22	diploid	https://figshare.com/s/565549fb2611c26c229f
Pueraria montana	*Pueraria*	*Phaseoleae*	*Fabaceae*	1380	1370	22	diploid	Provided by Dr. zheng dan W
Cajanus cajan	*Cajanus*	*Phaseoleae*	*Fabaceae*	605.8	594.8	22	diploid	https://cegresources.icrisat.org/data_public/legumepedia_data/Cajanus_cajan/
Spatholobus suberectus	*Spatholobus*	*Phaseoleae*	*Fabaceae*	793	798	18	diploid	https://www.ncbi.nlm.nih.gov/genome/76287?genome_assembly_id=467861
Abrus precatorius	*Abrus*	*Abreae*	*Fabaceae*	-	347.2	22	diploid	https://www.ncbi.nlm.nih.gov/genome/74709?genome_assembly_id=430755
Abrus melanospermus	*Abrus*	*Abreae*	*Fabaceae*	438	381.3	22	diploid	https://www.ncbi.nlm.nih.gov/genome/115036?genome_assembly_id=1887941
Pongamia pinnata	*Pongamia*	*Millettieae*	*Fabaceae*	-	1074	22	diploid	https://ngdc.cncb.ac.cn/gwh/Assembly/20699/show
Medicago polymorpha	*Medicago*	*Trifolieae*	*Fabaceae*	505.2	457.5	14	diploid	https://ngdc.cncb.ac.cn/search/?dbId=gwh&q=GWHANWO00000000.1
Medicago truncatula	*Medicago*	*Trifolieae*	*Fabaceae*	465	430	16	diploid	https://www.ncbi.nlm.nih.gov/genome/6?genome_assembly_id=406060
Medicago sativa	*Medicago*	*Trifolieae*	*Fabaceae*	802	793.2	16	diploid	https://figshare.com/search?q=A%20chromosome-scale
Medicago ruthenica	*Medicago*	*Trifolieae*	*Fabaceae*	914	904.1	16	diploid	https://figshare.com/articles/dataset/Medicago_ruthenica/12726932
Melilotus albus	*Melilotus*	*Trifolieae*	*Fabaceae*	1150	1050	16	diploid	https://ngdc.cncb.ac.cn/gwh/Assembly/22100/show
Trifolium pratense	*Trifolium*	*Trifolieae*	*Fabaceae*	420	309	14	diploid	https://zenodo.org/record/17232
Trifolium subterranum	*Trifolium*	*Trifolieae*	*Fabaceae*	488.8	473.2	16	diploid	https://cegresources.icrisat.org/data_public/legumepedia_data/Trifolium_subterraneum/

Trifolium occidentale	*Trifolium*	*Trifolieae*	*Fabaceae*	530	437	8	diploid	https://www.dropbox.com/sh/umfcbe1r8ajccwx/AABYufY6LYPiExxeh-RQXaToa?dl=0
Trifolium pallescens	*Trifolium*	*Trifolieae*	*Fabaceae*	534	382	8	diploid	https://www.dropbox.com/sh/umfcbe1r8ajccwx/AABYufY6LYPiExxeh-RQXaToa?dl=0
Trifolium repens	*Trifolium*	*Trifolieae*	*Fabaceae*	1174	841	16	allotetraploid	https://www.dropbox.com/sh/umfcbe1r8ajccwx/AABYufY6LYPiExxeh-RQXaToa?dl=0
Pisum sativum	*Pisum*	*Fabeae*	*Fabaceae*	4450	3920	14	diploid	https://urgi.versailles.inra.fr/download/pea/
Vicia sativa	*Vicia*	*Fabeae*	*Fabaceae*	1800	1650	12	diploid	http://gigadb.org/dataset/view/id/100954/File_page/4
Cicer arietinum	*Cicer*	*Cicereae*	*Fabaceae*	532.3	530.3	16	diploid	https://cegresources.icrisat.org/data_public/legumepedia_data/Cicer_arietinum/
Oxytropis ochrocephala	*Oxytropis*	*Galegeae*	*Fabaceae*	914.4	958.8	16	diploid	https://www.ncbi.nlm.nih.gov/genome/108437?genome_assembly_id=1742926
Astragalus sinicus	*Astragalus*	*Galegeae*	*Fabaceae*	625.2	595.5	16	diploid	Provided by Dr. Wei dong C
Glycyrrhiza uralensis	*Glycyrrhiza*	*Galegeae*	*Fabaceae*	397	379	16	diploid	https://github.com/BioproductivityInformatics ResearchTeam/Glycyrrhiza_uralensis_genome/tree/master/download
Lotus japonicus	*Lotus*	*Loteae*	*Fabaceae*	500	554.3	12	diploid	https://genomevolution.org/coge/GenomeInfo.pl?gid=58121
Dalbergia odorifera	*Dalbergia*	*Dalbergieae*	*Fabaceae*	653.5	623.5	20	diploid	http://gigadb.org/dataset/100760
Aeschynomene evenia	*Aeschynomene*	*Dalbergieae*	*Fabaceae*	400	376	20	diploid	http://aeschynomenebase.fr/download
Arachis hypogaea	*Arachis*	*Dalbergieae*	*Fabaceae*	2540	2502.6	40	allotetraploid	https://peanutbase.org/peanut_genome
Arachis monticola	*Arachis*	*Dalbergieae*	*Fabaceae*	2700	2620	40	allotetraploid	http://gigadb.org/dataset/100453
Arachis duranensis	*Arachis*	*Dalbergieae*	*Fabaceae*	1084.3	1067.5	20	diploid	https://cegresources.icrisat.org/data_public/legumepedia_data/Arachis_duranensis/

Arachis ipaensis	*Arachis*	*Dalbergieae*	*Fabaceae*	1353.8	1349.5	20	diploid	https://cegresources.icrisat.org/data_public/legumepedia_data/Arachis_ipaensis/
Nissolia schottii	*Nissolia*	*Dalbergieae*	*Fabaceae*	471	466	unknown	diploid	http://gigadb.org/search/new?keyword=Nissolia+schottii
Lupinus albus	*Lupinus*	*Genisteae*	*Fabaceae*	584.5	558.7	50	diploid	https://www.ncbi.nlm.nih.gov/genome/15582?genome_assembly_id=770531
Lupinus angustifolius	*Lupinus*	*Genisteae*	*Fabaceae*	-	615.8	40	diploid	https://www.ncbi.nlm.nih.gov/genome/11024?genome_assembly_id=293370
Ammopiptanthus nanus	*Ammopiptanthus*	*Sophoreae*	*Fabaceae*	889	823.7	18	diploid	http://www.gigadb.org/dataset/100466
Styphnolobium japonicum	*Styphnolobium*	*Sophoreae*	*Fabaceae*	535.8	511.5	28	diploid	https://www.ncbi.nlm.nih.gov/genome/80607?genome_assembly_id=1839231
Faidherbia albida	*Faidherbia*	*Ingeae*	*Fabaceae*	661	653.7	26	diploid	https://bioinformatics.psb.ugent.be/orcae/aocc/
Mimosa pudica	*Mimosa*	*Mimoseae*	*Fabaceae*	896	557	52	diploid	http://gigadb.org/search/new?keyword=Mimosa+pudica
Entada phaseoloides	*Entada*	*Mimoseae*	*Fabaceae*	549	456.2	28	diploid	https://ftp.cngb.org/pub/CNSA/data1/CNP0001900/CNS0383647/CNA0029377/
Chamaecrista fasciculata	*Chamaecrista*	*Cassieae*	*Fabaceae*	550	429	16	diploid	http://gigadb.org/search/new?keyword=Chamaecrista+fasciculata
Senna tora	*Senna*	*Cassieae*	*Fabaceae*	547	526.4	26	diploid	https://www.ncbi.nlm.nih.gov/genome/44961?genome_assembly_id=1470505
Cercis canadensis	*Cercis*	*Cercideae*	*Fabaceae*	301	330	14	diploid	http://gigadb.org/search/new?keyword=Cercis+canadensis
Bauhinia variegata	*Bauhinia*	*Cercideae*	*Fabaceae*	327	326.4	28	diploid	https://www.ncbi.nlm.nih.gov/genome/111100?genome_assembly_id=1866184
Sindora glabra	*Sindora*	*Detarieae*	*Fabaceae*	1220	1110	24	diploid	Provided by Dr. Niu Y

Vitis vinifera	*Vitis*	*Viteae*	*Vitaceae*	486	469	38	diploid	https://www.ncbi.nlm.nih.gov/genome/401?genome_assembly_id=214125

Supplemental Table 2. Detection and characteristics of intact LTR-RTs in 54 Fabaceae species.

Species	Assembled genome size (Mb)	Number of intact LTR-RTs	Cumulative length	Number of each type			Average length of each type (bp)		
				Copia	*Gypsy*	Unknown	*Copia*	*Gypsy*	Unknown
Vigna angularis	467.3	904	4146428	416	350	139	4384	5381	3192
Vigna radiata	475.2	1635	9953762	735	753	147	5536	7035	3994
Vigna unguiculata	549.8	1712	11022459	799	868	45	5140	7673	5680
Vigna subterranea	535.1	1199	6609467	692	461	46	4905	6587	3873
Phaseolus vulgaris	549.6	382	2337514	228	149	5	4648	8435	4215
Phaseolus acutifolius	661.9	2472	22565343	791	1661	20	6474	10439	5270
Phaseolus lunatus	623	1638	12285473	578	1029	31	5925	8447	5445
Lablab purpureus	395.5	249	1577276	152	92	5	5762	7496	2368
Glycine max	1015.4	2580	17742929	1563	952	65	7172	6634	3338
Glycine soja	1013.8	3230	22542598	1892	1255	83	7166	6902	3893
Glycine latifolia	939	1013	6636233	591	389	33	5516	8395	3356
Amphicarpaea edgeworthii	343.8	395	2342631	264	122	9	4798	8476	4643
Pueraria montana	1370	4483	33190327	2222	1768	493	6770	8981	4602
Cajanus cajan	594.8	760	4752988	329	367	64	4986	7713	4403
Spatholobus suberectus	798	1104	6950547	847	198	59	6292	7195	3329
Abrus precatorius	347.2	513	3741085	225	281	7	5095	9004	9207
Abrus melanospermus	381.3	768	5158635	433	323	12	4876	9167	7210
Pongamia pinnata	1074	4223	33202852	2236	1660	327	7043	8796	8730
Medicago polymorpha	457.5	851	6643060	491	276	84	7448	8987	6018
Medicago truncatula	430	1328	10758783	524	688	116	7163	9319	5122
Medicago sativa	793.2	2301	15106461	1035	747	519	5763	8861	4861
Medicago ruthenica	904.1	9510	79505891	4689	3478	1339	7522	9720	7776
Melilotus albus	1050	6594	55448277	3512	2308	774	8149	9451	6480

Trifolium pratense	309	93	348010	51	7	35	4189	5668	2706
Trifolium subterranum	473.2	483	2503409	303	85	95	5317	6256	3797
Trifolium occidentale	437	415	2075472	250	58	107	5023	8025	3312
Trifolium pallescens	382	213	1029357	127	25	61	5164	6795	3340
Trifolium repens	841	877	4741236	524	179	174	5019	7338	4585
Pisum sativum	3920	1857	13997349	1313	428	117	6843	10104	5925
Vicia sativa	1650	2387	19416879	1208	1024	156	5726	11132	7102
Cicer arietinum	530.3	553	2419309	395	56	102	4672	5555	2576
Oxytropis ochrocephala	958.8	2217	13171054	1258	675	284	5305	7932	4025
Astragalus sinicus	595.5	3685	32062547	1008	2443	234	5629	10385	4345
Glycyrrhiza uralensis	379	223	1072217	135	53	35	4864	5615	3369
Lotus japonicus	554.3	4591	38945335	2137	2232	222	8565	8824	4257
Dalbergia odorifera	623.5	1856	12172231	930	668	258	5359	8710	5324
Aeschynomene evenia	376	979	6495776	421	406	152	6695	7568	3978
Arachis hypogaea	2502.6	20209	194826443	2285	16255	1669	6525	10220	8266
Arachis monticola	2620	5828	49817779	1221	3842	765	5477	9688	7725
Arachis duranensis	1067.5	1219	9504697	306	700	213	5273	9147	6987
Arachis ipaensis	1349.5	2697	22902390	501	1710	486	5387	9567	7908
Nissolia schottii	466	491	2406258	193	187	111	5417	5628	2778
Lupinus albus	558.7	1213	8130713	766	419	28	6632	6968	4686
Lupinus angustifolius	615.8	718	3573589	501	169	49	5036	5680	1912
Ammopiptanthus nanus	823.7	3804	25295210	2252	1376	176	6501	7321	3300
Styphnolobium japonicum	511.5	1863	14330524	972	750	141	6484	10017	3653
Faidherbia albida	653.7	393	1970163	134	238	21	4824	5168	4471
Mimosa pudica	557	584	2939263	201	170	213	6295	5854	3187
Entada phaseoloides	456.2	129	686419	91	30	8	5345	5139	5727

Chamaecrista fasciculata	429	659	3537332	389	147	123	5073	8003	3149
Senna tora	526.4	1550	9694944	1013	430	109	5246	8989	4897
Cercis canadensis	330	561	3485758	293	245	23	5289	7398	5372
Bauhinia variegata	326.4	458	2597803	300	120	38	5060	7205	5661
Sindora glabra	1110	1270	9638467	640	628	2	6238	8986	1567

Supplemental Table 3. Characterizations of fragmented LTR-RT copies in Fabaceae species.

Species	Number of each type			Total length of each type (bp)			Genome percentage of each type (%)			
	Copia	*Gypsy*	Unknown	*Copia*	*Gypsy*	Unknown	Total	*Copia*	*Gypsy*	Unknown
Vigna angularis	103774	143481	54253	45126498	79057753	18887782	30.62	9.66	16.92	4.04
Vigna radiata	110352	137222	75482	62100459	90026814	26923661	37.68	13.07	18.95	5.67
Vigna unguiculata	131348	158621	34331	65053825	115991280	15998410	35.84	11.83	21.10	2.91
Vigna subterranea	183166	185662	58620	88430953	98847169	19529251	38.65	16.53	18.47	3.65
Phaseolus vulgaris	85254	110697	7181	56726578	113093786	3249864	31.49	10.32	20.58	0.59
Phaseolus acutifolius	76262	166535	29173	62616450	286540179	9136922	54.13	9.46	43.29	1.38
Phaseolus lunatus	77167	153043	34012	56730021	239907744	10390107	49.28	9.11	38.51	1.67
Lablab purpureus	91638	33861	1268	46488970	18078672	462248	16.44	11.76	4.57	0.12
Glycine max	118142	199488	21179	102669358	269490194	10832103	37.72	10.11	26.54	1.07
Glycine soja	93530	209068	104775	86759034	185386144	53253566	32.10	8.56	18.29	5.25
Glycine latifolia	171954	184018	25648	113871510	155537019	8628596	29.61	12.13	16.56	0.92
Amphicarpaea edgeworthii	36488	19132	15122	29939802	17612321	4197344	15.05	8.71	5.12	1.22
Pueraria montana	387913	224094	304537	192506999	186086910	96318960	34.67	14.05	13.58	7.03
Cajanus cajan	211416	261087	117465	76498751	133665702	35373449	41.28	12.86	22.47	5.95
Spatholobus suberectus	262665	129717	23583	142463742	78947957	8444618	28.80	17.85	9.89	1.06
Abrus precatorius	26888	53704	4876	16813194	51596175	2025120	20.28	4.84	14.86	0.58
Abrus melanospermus	31842	53225	11521	24077082	66117890	6836900	25.45	6.31	17.34	1.79
Pongamia pinnata	274327	250115	150303	204556097	269211769	105593076	53.94	19.05	25.07	9.83
Medicago polymorpha	82347	48541	46545	61047157	33161087	16591838	24.22	13.34	7.25	3.63
Medicago truncatula	76386	58984	68944	35588993	60024851	18235579	26.48	8.28	13.96	4.24
Medicago sativa	185680	137234	208999	121827273	147064213	70391615	42.77	15.36	18.54	8.87

Medicago ruthenica	119281	87162	55937	138421751	150650297	85397429	41.42	15.31	16.66	9.45
Melilotus albus	120631	146943	70542	177546088	233661413	78698424	46.66	16.91	22.25	7.50
Trifolium pratense	24233	8183	36135	6631456	2036805	6952189	5.06	2.15	0.66	2.25
Trifolium subterranum	127564	56631	88280	65416937	32985752	17881652	24.58	13.83	6.97	3.78
Trifolium occidentale	93682	44582	76006	37557589	24081920	19747342	18.62	8.59	5.51	4.52
Trifolium pallescens	49356	25362	49639	13594179	6840909	8301397	7.52	3.56	1.79	2.17
Trifolium repens	169683	91427	206387	79658111	54648467	57369153	22.79	9.47	6.50	6.82
Pisum sativum	891949	1290822	415584	864173720	1513162305	304074125	68.40	22.05	38.60	7.76
Vicia sativa	260375	367904	156402	274320761	668820055	138190443	65.54	16.63	40.53	8.38
Cicer arietinum	117107	63596	61580	81431243	38677995	25173904	27.40	15.36	7.29	4.75
Oxytropis ochrocephala	294828	301096	195409	151516634	284025428	96940912	55.53	15.80	29.62	10.11
Astragalus sinicus	107281	156987	47833	63707251	186502583	24733335	46.17	10.70	31.32	4.15
Glycyrrhiza uralensis	95045	31354	30610	36943301	11733427	6114308	14.46	9.75	3.10	1.61
Lotus japonicus	122737	126134	48015	102914588	133929163	14550661	45.36	18.57	24.16	2.63
Dalbergia odorifera	162774	137885	85650	88545450	129126204	39820673	41.30	14.20	20.71	6.39
Aeschynomene evenia	50249	74801	29523	56862467	62598277	13165345	35.27	15.12	16.65	3.50
Arachis hypogaea	61670	756336	138661	74685685	987428939	167058448	49.12	2.98	39.46	6.68
Arachis monticola	273004	837596	399210	173731150	1113825541	300666565	60.62	6.63	42.51	11.48
Arachis duranensis	83341	333859	185767	52006711	375727629	128454569	52.10	4.87	35.20	12.03
Arachis ipaensis	127647	501240	269505	76919718	612088800	200079132	65.88	5.70	45.36	14.83
Nissolia schottii	97839	123658	83947	48059448	77552607	26699431	32.68	10.31	16.64	5.73
Lupinus albus	121429	175070	46541	88955900	109314342	20434840	39.14	15.92	19.56	3.66
Lupinus angustifolius	293187	116007	44112	162218949	67634423	10002446	38.95	26.34	10.98	1.62
Ammopiptanthus nanus	302016	216438	67176	191201160	257561354	27308532	57.79	23.21	31.27	3.32

Styphnolobium japonicum	70754	57048	28193	63075059	59705810	10986544	26.15	12.33	11.67	2.15
Faidherbia albida	48433	150569	58467	28547533	110132484	39578678	27.27	4.37	16.85	6.05
Mimosa pudica	206582	53022	289133	67651042	29007180	58832850	27.92	12.15	5.21	10.56
Entada phaseoloides	78058	49786	33228	57423550	36990161	15500868	24.09	12.59	8.11	3.40
Chamaecrista fasciculata	262112	43996	73684	105568111	25279356	19741340	35.10	24.61	5.89	4.60
Senna tora	127779	145275	89004	57267064	133522599	30313262	42.00	10.88	25.37	5.76
Cercis canadensis	50096	35361	14468	25306460	33357971	5652166	19.49	7.67	10.11	1.71
Bauhinia variegata	14261	13917	7420	11618724	15564958	3771117	9.48	3.56	4.77	1.16
Sindora glabra	147226	88944	3139	117392305	76067940	1029915	17.52	10.58	6.85	0.09

Supplementary Table 4. The expression of each intact LTR-RT in *G. max* and *V. radiata.*

Glycine max			*Vigna radiata*		
LTR-RT_id	Counts	CPM	LTR-RT_id	Counts	CPM
LTRRT_1	1	98.87285	LTRRT_1	0	0
LTRRT_2	8	790.9828	LTRRT_2	0	0
LTRRT_3	0	0	LTRRT_3	1	35.447166
LTRRT_4	0	0	LTRRT_4	7	248.13016
LTRRT_5	2	197.7457	LTRRT_5	0	0
LTRRT_6	6	593.2371	LTRRT_6	1	35.447166
LTRRT_7	0	0	LTRRT_7	0	0
LTRRT_8	0	0	LTRRT_8	1	35.447166
LTRRT_9	0	0	LTRRT_9	42	1488.781
LTRRT_10	0	0	LTRRT_10	0	0
LTRRT_11	1	98.87285	LTRRT_11	0	0
LTRRT_12	0	0	LTRRT_12	23	815.28482
LTRRT_13	0	0	LTRRT_13	0	0
LTRRT_14	0	0	LTRRT_14	0	0
LTRRT_15	0	0	LTRRT_15	0	0
LTRRT_16	0	0	LTRRT_16	6	212.683
LTRRT_17	0	0	LTRRT_17	0	0
LTRRT_18	0	0	LTRRT_18	0	0
LTRRT_19	0	0	LTRRT_19	0	0
LTRRT_20	0	0	LTRRT_20	0	0
LTRRT_21	0	0	LTRRT_21	0	0
LTRRT_22	0	0	LTRRT_22	0	0
LTRRT_23	1	98.87285	LTRRT_23	0	0
LTRRT_24	0	0	LTRRT_24	0	0
LTRRT_25	0	0	LTRRT_25	0	0
LTRRT_26	0	0	LTRRT_26	0	0
LTRRT_27	0	0	LTRRT_27	0	0
LTRRT_28	0	0	LTRRT_28	0	0
LTRRT_29	0	0	LTRRT_29	0	0
LTRRT_30	0	0	LTRRT_30	0	0
LTRRT_31	2	197.7457	LTRRT_31	0	0
LTRRT_32	0	0	LTRRT_32	0	0
LTRRT_33	2	197.7457	LTRRT_33	0	0
LTRRT_34	0	0	LTRRT_34	0	0
LTRRT_35	0	0	LTRRT_35	4	141.78866
LTRRT_36	0	0	LTRRT_36	0	0
LTRRT_37	0	0	LTRRT_37	0	0
LTRRT_38	0	0	LTRRT_38	0	0
LTRRT_39	0	0	LTRRT_39	3	106.3415
LTRRT_40	0	0	LTRRT_40	0	0

LTRRT_41	0	0	LTRRT_41	0	0
LTRRT_42	0	0	LTRRT_42	0	0
LTRRT_43	4	395.4914	LTRRT_43	16	567.15466
LTRRT_44	0	0	LTRRT_44	24	850.73198
LTRRT_45	0	0	LTRRT_45	0	0
LTRRT_46	0	0	LTRRT_46	0	0
LTRRT_47	0	0	LTRRT_47	0	0
LTRRT_48	0	0	LTRRT_48	0	0
LTRRT_49	10	988.7285	LTRRT_49	4	141.78866
LTRRT_50	0	0	LTRRT_50	0	0
LTRRT_51	0	0	LTRRT_51	0	0
LTRRT_52	0	0	LTRRT_52	0	0
LTRRT_53	0	0	LTRRT_53	0	0
LTRRT_54	0	0	LTRRT_54	11	389.91883
LTRRT_55	0	0	LTRRT_55	17	602.60182
LTRRT_56	2	197.7457	LTRRT_56	0	0
LTRRT_57	0	0	LTRRT_57	0	0
LTRRT_58	49	4844.7696	LTRRT_58	0	0
LTRRT_59	0	0	LTRRT_59	2	70.894332
LTRRT_60	2	197.7457	LTRRT_60	5	177.23583
LTRRT_61	0	0	LTRRT_61	0	0
LTRRT_62	1	98.87285	LTRRT_62	0	0
LTRRT_63	0	0	LTRRT_63	0	0
LTRRT_64	1	98.87285	LTRRT_64	0	0
LTRRT_65	0	0	LTRRT_65	0	0
LTRRT_66	2	197.7457	LTRRT_66	0	0
LTRRT_67	0	0	LTRRT_67	27	957.07348
LTRRT_68	0	0	LTRRT_68	1	35.447166
LTRRT_69	0	0	LTRRT_69	0	0
LTRRT_70	1	98.87285	LTRRT_70	0	0
LTRRT_71	0	0	LTRRT_71	1	35.447166
LTRRT_72	0	0	LTRRT_72	0	0
LTRRT_73	7	692.10995	LTRRT_73	0	0
LTRRT_74	2	197.7457	LTRRT_74	0	0
LTRRT_75	0	0	LTRRT_75	13	460.81316
LTRRT_76	0	0	LTRRT_76	0	0
LTRRT_77	4	395.4914	LTRRT_77	3	106.3415
LTRRT_78	0	0	LTRRT_78	185	6557.7257
LTRRT_79	3	296.61855	LTRRT_79	0	0
LTRRT_80	0	0	LTRRT_80	0	0
LTRRT_81	2	197.7457	LTRRT_81	0	0
LTRRT_82	0	0	LTRRT_82	6	212.683
LTRRT_83	0	0	LTRRT_83	0	0
LTRRT_84	7	692.10995	LTRRT_84	0	0

LTRRT_85	3	296.61855	LTRRT_85	0	0
LTRRT_86	0	0	LTRRT_86	0	0
LTRRT_87	1	98.87285	LTRRT_87	0	0
LTRRT_88	0	0	LTRRT_88	4	141.78866
LTRRT_89	0	0	LTRRT_89	0	0
LTRRT_90	63	6228.9895	LTRRT_90	0	0
LTRRT_91	1	98.87285	LTRRT_91	0	0
LTRRT_92	0	0	LTRRT_92	0	0
LTRRT_93	0	0	LTRRT_93	0	0
LTRRT_94	0	0	LTRRT_94	9	319.02449
LTRRT_95	0	0	LTRRT_95	0	0
LTRRT_96	1	98.87285	LTRRT_96	0	0
LTRRT_97	0	0	LTRRT_97	107	3792.8468
LTRRT_98	8	790.9828	LTRRT_98	1	35.447166
LTRRT_99	0	0	LTRRT_99	0	0
LTRRT_100	2	197.7457	LTRRT_100	0	0
LTRRT_101	0	0	LTRRT_101	0	0
LTRRT_102	0	0	LTRRT_102	0	0
LTRRT_103	0	0	LTRRT_103	0	0
LTRRT_104	0	0	LTRRT_104	0	0
LTRRT_105	0	0	LTRRT_105	0	0
LTRRT_106	0	0	LTRRT_106	0	0
LTRRT_107	0	0	LTRRT_107	0	0
LTRRT_108	0	0	LTRRT_108	0	0
LTRRT_109	12	1186.4742	LTRRT_109	0	0
LTRRT_110	1	98.87285	LTRRT_110	0	0
LTRRT_111	2	197.7457	LTRRT_111	0	0
LTRRT_112	0	0	LTRRT_112	11	389.91883
LTRRT_113	2	197.7457	LTRRT_113	9	319.02449
LTRRT_114	0	0	LTRRT_114	0	0
LTRRT_115	0	0	LTRRT_115	0	0
LTRRT_116	1	98.87285	LTRRT_116	0	0
LTRRT_117	0	0	LTRRT_117	0	0
LTRRT_118	0	0	LTRRT_118	0	0
LTRRT_119	0	0	LTRRT_119	0	0
LTRRT_120	0	0	LTRRT_120	0	0
LTRRT_121	0	0	LTRRT_121	2	70.894332
LTRRT_122	0	0	LTRRT_122	0	0
LTRRT_123	0	0	LTRRT_123	0	0
LTRRT_124	0	0	LTRRT_124	0	0
LTRRT_125	9	889.85565	LTRRT_125	0	0
LTRRT_126	0	0	LTRRT_126	0	0
LTRRT_127	0	0	LTRRT_127	2	70.894332
LTRRT_128	0	0	LTRRT_128	4	141.78866

LTRRT_129	0	0	LTRRT_129	0	0
LTRRT_130	0	0	LTRRT_130	0	0
LTRRT_131	0	0	LTRRT_131	22	779.83765
LTRRT_132	0	0	LTRRT_132	0	0
LTRRT_133	0	0	LTRRT_133	6	212.683
LTRRT_134	0	0	LTRRT_134	0	0
LTRRT_135	7	692.10995	LTRRT_135	0	0
LTRRT_136	3	296.61855	LTRRT_136	0	0
LTRRT_137	11	1087.6013	LTRRT_137	0	0
LTRRT_138	5	494.36425	LTRRT_138	0	0
LTRRT_139	0	0	LTRRT_139	6	212.683
LTRRT_140	2	197.7457	LTRRT_140	0	0
LTRRT_141	0	0	LTRRT_141	2	70.894332
LTRRT_142	0	0	LTRRT_142	3	106.3415
LTRRT_143	0	0	LTRRT_143	0	0
LTRRT_144	0	0	LTRRT_144	0	0
LTRRT_145	0	0	LTRRT_145	0	0
LTRRT_146	0	0	LTRRT_146	30	1063.415
LTRRT_147	0	0	LTRRT_147	0	0
LTRRT_148	0	0	LTRRT_148	11	389.91883
LTRRT_149	0	0	LTRRT_149	0	0
LTRRT_150	0	0	LTRRT_150	0	0
LTRRT_151	1	98.87285	LTRRT_151	1	35.447166
LTRRT_152	0	0	LTRRT_152	0	0
LTRRT_153	3	296.61855	LTRRT_153	3	106.3415
LTRRT_154	1	98.87285	LTRRT_154	3	106.3415
LTRRT_155	0	0	LTRRT_155	0	0
LTRRT_156	4	395.4914	LTRRT_156	0	0
LTRRT_157	0	0	LTRRT_157	0	0
LTRRT_158	0	0	LTRRT_158	1	35.447166
LTRRT_159	0	0	LTRRT_159	0	0
LTRRT_160	1	98.87285	LTRRT_160	0	0
LTRRT_161	6	593.2371	LTRRT_161	0	0
LTRRT_162	0	0	LTRRT_162	0	0
LTRRT_163	0	0	LTRRT_163	0	0
LTRRT_164	4	395.4914	LTRRT_164	5	177.23583
LTRRT_165	0	0	LTRRT_165	0	0
LTRRT_166	1	98.87285	LTRRT_166	3	106.3415
LTRRT_167	24	2372.9484	LTRRT_167	17	602.60182
LTRRT_168	0	0	LTRRT_168	22	779.83765
LTRRT_169	27	2669.5669	LTRRT_169	0	0
LTRRT_170	0	0	LTRRT_170	11	389.91883
LTRRT_171	0	0	LTRRT_171	1	35.447166
LTRRT_172	0	0	LTRRT_172	1404	49767.821

LTRRT_173	0	0	LTRRT_173	0	0
LTRRT_174	0	0	LTRRT_174	0	0
LTRRT_175	0	0	LTRRT_175	0	0
LTRRT_176	0	0	LTRRT_176	0	0
LTRRT_177	0	0	LTRRT_177	0	0
LTRRT_178	0	0	LTRRT_178	0	0
LTRRT_179	5	494.36425	LTRRT_179	0	0
LTRRT_180	0	0	LTRRT_180	0	0
LTRRT_181	0	0	LTRRT_181	0	0
LTRRT_182	0	0	LTRRT_182	0	0
LTRRT_183	0	0	LTRRT_183	0	0
LTRRT_184	34	3361.6769	LTRRT_184	2	70.894332
LTRRT_185	2	197.7457	LTRRT_185	0	0
LTRRT_186	0	0	LTRRT_186	1	35.447166
LTRRT_187	4	395.4914	LTRRT_187	3	106.3415
LTRRT_188	0	0	LTRRT_188	0	0
LTRRT_189	0	0	LTRRT_189	0	0
LTRRT_190	0	0	LTRRT_190	11	389.91883
LTRRT_191	1	98.87285	LTRRT_191	0	0
LTRRT_192	0	0	LTRRT_192	0	0
LTRRT_193	0	0	LTRRT_193	0	0
LTRRT_194	1	98.87285	LTRRT_194	2	70.894332
LTRRT_195	0	0	LTRRT_195	0	0
LTRRT_196	0	0	LTRRT_196	0	0
LTRRT_197	0	0	LTRRT_197	0	0
LTRRT_198	0	0	LTRRT_198	0	0
LTRRT_199	0	0	LTRRT_199	0	0
LTRRT_200	19	1878.5841	LTRRT_200	0	0
LTRRT_201	1	98.87285	LTRRT_201	0	0
LTRRT_202	0	0	LTRRT_202	0	0
LTRRT_203	0	0	LTRRT_203	0	0
LTRRT_204	0	0	LTRRT_204	3	106.3415
LTRRT_205	0	0	LTRRT_205	0	0
LTRRT_206	0	0	LTRRT_206	0	0
LTRRT_207	0	0	LTRRT_207	452	16022.119
LTRRT_208	7	692.10995	LTRRT_208	0	0
LTRRT_209	0	0	LTRRT_209	35	1240.6508
LTRRT_210	1	98.87285	LTRRT_210	0	0
LTRRT_211	20	1977.457	LTRRT_211	0	0
LTRRT_212	1	98.87285	LTRRT_212	0	0
LTRRT_213	0	0	LTRRT_213	0	0
LTRRT_214	2	197.7457	LTRRT_214	0	0
LTRRT_215	0	0	LTRRT_215	4	141.78866
LTRRT_216	0	0	LTRRT_216	0	0

LTRRT_217	0	0	LTRRT_217	0	0
LTRRT_218	0	0	LTRRT_218	0	0
LTRRT_219	0	0	LTRRT_219	0	0
LTRRT_220	0	0	LTRRT_220	3	106.3415
LTRRT_221	0	0	LTRRT_221	0	0
LTRRT_222	0	0	LTRRT_222	0	0
LTRRT_223	0	0	LTRRT_223	0	0
LTRRT_224	3	296.61855	LTRRT_224	0	0
LTRRT_225	32	3163.9312	LTRRT_225	0	0
LTRRT_226	2	197.7457	LTRRT_226	11	389.91883
LTRRT_227	1	98.87285	LTRRT_227	0	0
LTRRT_228	0	0	LTRRT_228	0	0
LTRRT_229	0	0	LTRRT_229	0	0
LTRRT_230	0	0	LTRRT_230	12	425.36599
LTRRT_231	0	0	LTRRT_231	0	0
LTRRT_232	3	296.61855	LTRRT_232	0	0
LTRRT_233	2	197.7457	LTRRT_233	0	0
LTRRT_234	0	0	LTRRT_234	0	0
LTRRT_235	0	0	LTRRT_235	3	106.3415
LTRRT_236	0	0	LTRRT_236	0	0
LTRRT_237	1	98.87285	LTRRT_237	0	0
LTRRT_238	2	197.7457	LTRRT_238	0	0
LTRRT_239	1	98.87285	LTRRT_239	2	70.894332
LTRRT_240	0	0	LTRRT_240	0	0
LTRRT_241	0	0	LTRRT_241	8	283.57733
LTRRT_242	0	0	LTRRT_242	0	0
LTRRT_243	0	0	LTRRT_243	0	0
LTRRT_244	0	0	LTRRT_244	0	0
LTRRT_245	0	0	LTRRT_245	0	0
LTRRT_246	0	0	LTRRT_246	0	0
LTRRT_247	3	296.61855	LTRRT_247	0	0
LTRRT_248	0	0	LTRRT_248	0	0
LTRRT_249	0	0	LTRRT_249	0	0
LTRRT_250	2	197.7457	LTRRT_250	7	248.13016
LTRRT_251	0	0	LTRRT_251	0	0
LTRRT_252	5	494.36425	LTRRT_252	0	0
LTRRT_253	0	0	LTRRT_253	0	0
LTRRT_254	0	0	LTRRT_254	0	0
LTRRT_255	0	0	LTRRT_255	5	177.23583
LTRRT_256	0	0	LTRRT_256	21	744.39049
LTRRT_257	0	0	LTRRT_257	0	0
LTRRT_258	0	0	LTRRT_258	30	1063.415
LTRRT_259	0	0	LTRRT_259	1	35.447166
LTRRT_260	0	0	LTRRT_260	14	496.26032

LTRRT_261	0	0	LTRRT_261	380	13469.923
LTRRT_262	0	0	LTRRT_262	0	0
LTRRT_263	0	0	LTRRT_263	0	0
LTRRT_264	0	0	LTRRT_264	0	0
LTRRT_265	1	98.87285	LTRRT_265	0	0
LTRRT_266	0	0	LTRRT_266	0	0
LTRRT_267	0	0	LTRRT_267	143	5068.9447
LTRRT_268	0	0	LTRRT_268	1	35.447166
LTRRT_269	0	0	LTRRT_269	7	248.13016
LTRRT_270	0	0	LTRRT_270	28	992.52065
LTRRT_271	0	0	LTRRT_271	0	0
LTRRT_272	2	197.7457	LTRRT_272	0	0
LTRRT_273	0	0	LTRRT_273	0	0
LTRRT_274	0	0	LTRRT_274	3	106.3415
LTRRT_275	0	0	LTRRT_275	10	354.47166
LTRRT_276	0	0	LTRRT_276	0	0
LTRRT_277	0	0	LTRRT_277	0	0
LTRRT_278	0	0	LTRRT_278	0	0
LTRRT_279	4	395.4914	LTRRT_279	0	0
LTRRT_280	10	988.7285	LTRRT_280	0	0
LTRRT_281	0	0	LTRRT_281	11	389.91883
LTRRT_282	0	0	LTRRT_282	113	4005.5298
LTRRT_283	4	395.4914	LTRRT_283	0	0
LTRRT_284	0	0	LTRRT_284	0	0
LTRRT_285	0	0	LTRRT_285	0	0
LTRRT_286	0	0	LTRRT_286	0	0
LTRRT_287	0	0	LTRRT_287	0	0
LTRRT_288	0	0	LTRRT_288	423	14994.151
LTRRT_289	0	0	LTRRT_289	0	0
LTRRT_290	0	0	LTRRT_290	1	35.447166
LTRRT_291	0	0	LTRRT_291	0	0
LTRRT_292	0	0	LTRRT_292	0	0
LTRRT_293	1	98.87285	LTRRT_293	0	0
LTRRT_294	0	0	LTRRT_294	0	0
LTRRT_295	1	98.87285	LTRRT_295	1	35.447166
LTRRT_296	0	0	LTRRT_296	0	0
LTRRT_297	0	0	LTRRT_297	0	0
LTRRT_298	27	2669.5669	LTRRT_298	19	673.49615
LTRRT_299	1	98.87285	LTRRT_299	0	0
LTRRT_300	1	98.87285	LTRRT_300	57	2020.4885
LTRRT_301	8	790.9828	LTRRT_301	1	35.447166
LTRRT_302	0	0	LTRRT_302	0	0
LTRRT_303	3	296.61855	LTRRT_303	0	0
LTRRT_304	5	494.36425	LTRRT_304	0	0

LTRRT_305	0	0	LTRRT_305	0	0
LTRRT_306	0	0	LTRRT_306	0	0
LTRRT_307	3	296.61855	LTRRT_307	21	744.39049
LTRRT_308	53	5240.261	LTRRT_308	16	567.15466
LTRRT_309	0	0	LTRRT_309	0	0
LTRRT_310	4	395.4914	LTRRT_310	0	0
LTRRT_311	46	4548.1511	LTRRT_311	0	0
LTRRT_312	0	0	LTRRT_312	0	0
LTRRT_313	0	0	LTRRT_313	0	0
LTRRT_314	3	296.61855	LTRRT_314	1	35.447166
LTRRT_315	0	0	LTRRT_315	0	0
LTRRT_316	0	0	LTRRT_316	0	0
LTRRT_317	0	0	LTRRT_317	15	531.70749
LTRRT_318	0	0	LTRRT_318	0	0
LTRRT_319	0	0	LTRRT_319	0	0
LTRRT_320	0	0	LTRRT_320	25	886.17915
LTRRT_321	55	5438.0067	LTRRT_321	11	389.91883
LTRRT_322	0	0	LTRRT_322	0	0
LTRRT_323	0	0	LTRRT_323	3	106.3415
LTRRT_324	0	0	LTRRT_324	0	0
LTRRT_325	0	0	LTRRT_325	36	1276.098
LTRRT_326	1	98.87285	LTRRT_326	0	0
LTRRT_327	13	1285.347	LTRRT_327	0	0
LTRRT_328	0	0	LTRRT_328	0	0
LTRRT_329	0	0	LTRRT_329	0	0
LTRRT_330	0	0	LTRRT_330	36	1276.098
LTRRT_331	3	296.61855	LTRRT_331	0	0
LTRRT_332	1	98.87285	LTRRT_332	22	779.83765
LTRRT_333	7	692.10995	LTRRT_333	14	496.26032
LTRRT_334	0	0	LTRRT_334	0	0
LTRRT_335	2	197.7457	LTRRT_335	4	141.78866
LTRRT_336	14	1384.2199	LTRRT_336	0	0
LTRRT_337	0	0	LTRRT_337	50	1772.3583
LTRRT_338	0	0	LTRRT_338	1	35.447166
LTRRT_339	4	395.4914	LTRRT_339	0	0
LTRRT_340	0	0	LTRRT_340	51	1807.8055
LTRRT_341	0	0	LTRRT_341	0	0
LTRRT_342	0	0	LTRRT_342	0	0
LTRRT_343	0	0	LTRRT_343	4	141.78866
LTRRT_344	0	0	LTRRT_344	0	0
LTRRT_345	3	296.61855	LTRRT_345	22	779.83765
LTRRT_346	0	0	LTRRT_346	2	70.894332
LTRRT_347	0	0	LTRRT_347	0	0
LTRRT_348	0	0	LTRRT_348	0	0

LTRRT_349	0	0	LTRRT_349	0	0
LTRRT_350	29	2867.3126	LTRRT_350	4	141.78866
LTRRT_351	0	0	LTRRT_351	0	0
LTRRT_352	0	0	LTRRT_352	7	248.13016
LTRRT_353	27	2669.5669	LTRRT_353	0	0
LTRRT_354	0	0	LTRRT_354	41	1453.3338
LTRRT_355	0	0	LTRRT_355	0	0
LTRRT_356	0	0	LTRRT_356	9	319.02449
LTRRT_357	0	0	LTRRT_357	235	8330.084
LTRRT_358	0	0	LTRRT_358	0	0
LTRRT_359	1	98.87285	LTRRT_359	0	0
LTRRT_360	0	0	LTRRT_360	12	425.36599
LTRRT_361	0	0	LTRRT_361	0	0
LTRRT_362	0	0	LTRRT_362	153	5423.4164
LTRRT_363	0	0	LTRRT_363	0	0
LTRRT_364	0	0	LTRRT_364	2	70.894332
LTRRT_365	7	692.10995	LTRRT_365	24	850.73198
LTRRT_366	0	0	LTRRT_366	0	0
LTRRT_367	0	0	LTRRT_367	8	283.57733
LTRRT_368	0	0	LTRRT_368	0	0
LTRRT_369	0	0	LTRRT_369	27	957.07348
LTRRT_370	1	98.87285	LTRRT_370	2	70.894332
LTRRT_371	1	98.87285	LTRRT_371	0	0
LTRRT_372	0	0	LTRRT_372	38	1346.9923
LTRRT_373	0	0	LTRRT_373	0	0
LTRRT_374	0	0	LTRRT_374	0	0
LTRRT_375	0	0	LTRRT_375	0	0
LTRRT_376	0	0	LTRRT_376	0	0
LTRRT_377	0	0	LTRRT_377	0	0
LTRRT_378	0	0	LTRRT_378	0	0
LTRRT_379	0	0	LTRRT_379	0	0
LTRRT_380	0	0	LTRRT_380	0	0
LTRRT_381	0	0	LTRRT_381	4	141.78866
LTRRT_382	0	0	LTRRT_382	0	0
LTRRT_383	0	0	LTRRT_383	0	0
LTRRT_384	0	0	LTRRT_384	0	0
LTRRT_385	3	296.61855	LTRRT_385	0	0
LTRRT_386	0	0	LTRRT_386	0	0
LTRRT_387	6	593.2371	LTRRT_387	81	2871.2204
LTRRT_388	0	0	LTRRT_388	1	35.447166
LTRRT_389	0	0	LTRRT_389	0	0
LTRRT_390	0	0	LTRRT_390	1	35.447166
LTRRT_391	0	0	LTRRT_391	44	1559.6753
LTRRT_392	2	197.7457	LTRRT_392	0	0

LTRRT_393	0	0	LTRRT_393	0	0
LTRRT_394	0	0	LTRRT_394	0	0
LTRRT_395	3	296.61855	LTRRT_395	0	0
LTRRT_396	0	0	LTRRT_396	0	0
LTRRT_397	0	0	LTRRT_397	0	0
LTRRT_398	1	98.87285	LTRRT_398	0	0
LTRRT_399	5	494.36425	LTRRT_399	1	35.447166
LTRRT_400	0	0	LTRRT_400	1	35.447166
LTRRT_401	0	0	LTRRT_401	1	35.447166
LTRRT_402	0	0	LTRRT_402	0	0
LTRRT_403	0	0	LTRRT_403	0	0
LTRRT_404	0	0	LTRRT_404	0	0
LTRRT_405	0	0	LTRRT_405	0	0
LTRRT_406	0	0	LTRRT_406	0	0
LTRRT_407	0	0	LTRRT_407	0	0
LTRRT_408	0	0	LTRRT_408	0	0
LTRRT_409	0	0	LTRRT_409	0	0
LTRRT_410	1	98.87285	LTRRT_410	0	0
LTRRT_411	0	0	LTRRT_411	0	0
LTRRT_412	0	0	LTRRT_412	0	0
LTRRT_413	0	0	LTRRT_413	0	0
LTRRT_414	1	98.87285	LTRRT_414	0	0
LTRRT_415	0	0	LTRRT_415	0	0
LTRRT_416	0	0	LTRRT_416	0	0
LTRRT_417	0	0	LTRRT_417	0	0
LTRRT_418	0	0	LTRRT_418	0	0
LTRRT_419	2	197.7457	LTRRT_419	0	0
LTRRT_420	0	0	LTRRT_420	0	0
LTRRT_421	0	0	LTRRT_421	0	0
LTRRT_422	0	0	LTRRT_422	0	0
LTRRT_423	0	0	LTRRT_423	0	0
LTRRT_424	0	0	LTRRT_424	2	70.894332
LTRRT_425	0	0	LTRRT_425	0	0
LTRRT_426	0	0	LTRRT_426	13	460.81316
LTRRT_427	0	0	LTRRT_427	39	1382.4395
LTRRT_428	1	98.87285	LTRRT_428	3	106.3415
LTRRT_429	0	0	LTRRT_429	19	673.49615
LTRRT_430	0	0	LTRRT_430	0	0
LTRRT_431	0	0	LTRRT_431	5	177.23583
LTRRT_432	10	988.7285	LTRRT_432	0	0
LTRRT_433	0	0	LTRRT_433	0	0
LTRRT_434	29	2867.3126	LTRRT_434	0	0
LTRRT_435	6	593.2371	LTRRT_435	0	0
LTRRT_436	0	0	LTRRT_436	0	0

LTRRT_437	2	197.7457	LTRRT_437	0	0
LTRRT_438	8	790.9828	LTRRT_438	170	6026.0182
LTRRT_439	0	0	LTRRT_439	0	0
LTRRT_440	0	0	LTRRT_440	1	35.447166
LTRRT_441	0	0	LTRRT_441	0	0
LTRRT_442	3	296.61855	LTRRT_442	0	0
LTRRT_443	67	6624.4809	LTRRT_443	0	0
LTRRT_444	1	98.87285	LTRRT_444	3	106.3415
LTRRT_445	0	0	LTRRT_445	0	0
LTRRT_446	0	0	LTRRT_446	0	0
LTRRT_447	6	593.2371	LTRRT_447	0	0
LTRRT_448	0	0	LTRRT_448	0	0
LTRRT_449	0	0	LTRRT_449	7	248.13016
LTRRT_450	0	0	LTRRT_450	0	0
LTRRT_451	13	1285.347	LTRRT_451	0	0
LTRRT_452	2	197.7457	LTRRT_452	0	0
LTRRT_453	43	4251.5325	LTRRT_453	7	248.13016
LTRRT_454	0	0	LTRRT_454	0	0
LTRRT_455	0	0	LTRRT_455	0	0
LTRRT_456	0	0	LTRRT_456	0	0
LTRRT_457	0	0	LTRRT_457	6	212.683
LTRRT_458	3	296.61855	LTRRT_458	0	0
LTRRT_459	0	0	LTRRT_459	1	35.447166
LTRRT_460	1	98.87285	LTRRT_460	0	0
LTRRT_461	6	593.2371	LTRRT_461	384	13611.712
LTRRT_462	0	0	LTRRT_462	48	1701.464
LTRRT_463	0	0	LTRRT_463	0	0
LTRRT_464	1	98.87285	LTRRT_464	0	0
LTRRT_465	0	0	LTRRT_465	11	389.91883
LTRRT_466	0	0	LTRRT_466	2	70.894332
LTRRT_467	11	1087.6013	LTRRT_467	0	0
LTRRT_468	0	0	LTRRT_468	0	0
LTRRT_469	20	1977.457	LTRRT_469	0	0
LTRRT_470	0	0	LTRRT_470	6	212.683
LTRRT_471	0	0	LTRRT_471	0	0
LTRRT_472	0	0	LTRRT_472	31	1098.8621
LTRRT_473	2	197.7457	LTRRT_473	2	70.894332
LTRRT_474	2	197.7457	LTRRT_474	0	0
LTRRT_475	0	0	LTRRT_475	28	992.52065
LTRRT_476	0	0	LTRRT_476	0	0
LTRRT_477	1	98.87285	LTRRT_477	0	0
LTRRT_478	0	0	LTRRT_478	2	70.894332
LTRRT_479	0	0	LTRRT_479	52	1843.2526
LTRRT_480	0	0	LTRRT_480	216	7656.5879

LTRRT_481	0	0	LTRRT_481	10	354.47166
LTRRT_482	21	2076.3298	LTRRT_482	0	0
LTRRT_483	1	98.87285	LTRRT_483	0	0
LTRRT_484	0	0	LTRRT_484	0	0
LTRRT_485	0	0	LTRRT_485	0	0
LTRRT_486	0	0	LTRRT_486	53	1878.6998
LTRRT_487	0	0	LTRRT_487	0	0
LTRRT_488	0	0	LTRRT_488	6	212.683
LTRRT_489	0	0	LTRRT_489	1	35.447166
LTRRT_490	0	0	LTRRT_490	0	0
LTRRT_491	0	0	LTRRT_491	0	0
LTRRT_492	13	1285.347	LTRRT_492	0	0
LTRRT_493	0	0	LTRRT_493	0	0
LTRRT_494	0	0	LTRRT_494	0	0
LTRRT_495	0	0	LTRRT_495	0	0
LTRRT_496	0	0	LTRRT_496	0	0
LTRRT_497	0	0	LTRRT_497	0	0
LTRRT_498	0	0	LTRRT_498	0	0
LTRRT_499	0	0	LTRRT_499	0	0
LTRRT_500	0	0	LTRRT_500	0	0
LTRRT_501	0	0	LTRRT_501	3	106.3415
LTRRT_502	0	0	LTRRT_502	0	0
LTRRT_503	6	593.2371	LTRRT_503	0	0
LTRRT_504	6	593.2371	LTRRT_504	0	0
LTRRT_505	0	0	LTRRT_505	0	0
LTRRT_506	1	98.87285	LTRRT_506	3	106.3415
LTRRT_507	0	0	LTRRT_507	0	0
LTRRT_508	0	0	LTRRT_508	1	35.447166
LTRRT_509	0	0	LTRRT_509	13	460.81316
LTRRT_510	3	296.61855	LTRRT_510	0	0
LTRRT_511	5	494.36425	LTRRT_511	7	248.13016
LTRRT_512	0	0	LTRRT_512	0	0
LTRRT_513	0	0	LTRRT_513	0	0
LTRRT_514	0	0	LTRRT_514	0	0
LTRRT_515	0	0	LTRRT_515	0	0
LTRRT_516	0	0	LTRRT_516	0	0
LTRRT_517	0	0	LTRRT_517	0	0
LTRRT_518	0	0	LTRRT_518	0	0
LTRRT_519	0	0	LTRRT_519	12	425.36599
LTRRT_520	0	0	LTRRT_520	0	0
LTRRT_521	0	0	LTRRT_521	0	0
LTRRT_522	2	197.7457	LTRRT_522	108	3828.2939
LTRRT_523	0	0	LTRRT_523	0	0
LTRRT_524	5	494.36425	LTRRT_524	0	0

LTRRT_525	0	0	LTRRT_525	6	212.683
LTRRT_526	0	0	LTRRT_526	0	0
LTRRT_527	2	197.7457	LTRRT_527	0	0
LTRRT_528	1	98.87285	LTRRT_528	0	0
LTRRT_529	0	0	LTRRT_529	4	141.78866
LTRRT_530	0	0	LTRRT_530	0	0
LTRRT_531	1	98.87285	LTRRT_531	0	0
LTRRT_532	1	98.87285	LTRRT_532	1	35.447166
LTRRT_533	1	98.87285	LTRRT_533	177	6274.1484
LTRRT_534	0	0	LTRRT_534	0	0
LTRRT_535	0	0	LTRRT_535	0	0
LTRRT_536	0	0	LTRRT_536	0	0
LTRRT_537	0	0	LTRRT_537	0	0
LTRRT_538	0	0	LTRRT_538	0	0
LTRRT_539	0	0	LTRRT_539	0	0
LTRRT_540	0	0	LTRRT_540	0	0
LTRRT_541	0	0	LTRRT_541	0	0
LTRRT_542	0	0	LTRRT_542	582	20630.251
LTRRT_543	0	0	LTRRT_543	0	0
LTRRT_544	0	0	LTRRT_544	535	18964.234
LTRRT_545	1	98.87285	LTRRT_545	41	1453.3338
LTRRT_546	1	98.87285	LTRRT_546	0	0
LTRRT_547	0	0	LTRRT_547	0	0
LTRRT_548	0	0	LTRRT_548	0	0
LTRRT_549	0	0	LTRRT_549	0	0
LTRRT_550	0	0	LTRRT_550	0	0
LTRRT_551	0	0	LTRRT_551	0	0
LTRRT_552	0	0	LTRRT_552	0	0
LTRRT_553	0	0	LTRRT_553	0	0
LTRRT_554	0	0	LTRRT_554	0	0
LTRRT_555	0	0	LTRRT_555	0	0
LTRRT_556	0	0	LTRRT_556	4	141.78866
LTRRT_557	557	55072.177	LTRRT_557	5	177.23583
LTRRT_558	0	0	LTRRT_558	54	1914.147
LTRRT_559	0	0	LTRRT_559	0	0
LTRRT_560	58	5734.6253	LTRRT_560	28	992.52065
LTRRT_561	1	98.87285	LTRRT_561	0	0
LTRRT_562	0	0	LTRRT_562	13	460.81316
LTRRT_563	2	197.7457	LTRRT_563	5	177.23583
LTRRT_564	0	0	LTRRT_564	5	177.23583
LTRRT_565	1	98.87285	LTRRT_565	0	0
LTRRT_566	0	0	LTRRT_566	0	0
LTRRT_567	0	0	LTRRT_567	0	0
LTRRT_568	0	0	LTRRT_568	0	0

LTRRT_569	2	197.7457	LTRRT_569	0	0
LTRRT_570	0	0	LTRRT_570	0	0
LTRRT_571	1	98.87285	LTRRT_571	48	1701.464
LTRRT_572	0	0	LTRRT_572	0	0
LTRRT_573	0	0	LTRRT_573	1	35.447166
LTRRT_574	0	0	LTRRT_574	0	0
LTRRT_575	0	0	LTRRT_575	0	0
LTRRT_576	0	0	LTRRT_576	2	70.894332
LTRRT_577	0	0	LTRRT_577	3	106.3415
LTRRT_578	1	98.87285	LTRRT_578	0	0
LTRRT_579	0	0	LTRRT_579	0	0
LTRRT_580	0	0	LTRRT_580	0	0
LTRRT_581	1	98.87285	LTRRT_581	0	0
LTRRT_582	2	197.7457	LTRRT_582	0	0
LTRRT_583	0	0	LTRRT_583	0	0
LTRRT_584	2	197.7457	LTRRT_584	0	0
LTRRT_585	2	197.7457	LTRRT_585	0	0
LTRRT_586	0	0	LTRRT_586	80	2835.7733
LTRRT_587	0	0	LTRRT_587	7	248.13016
LTRRT_588	5	494.36425	LTRRT_588	12	425.36599
LTRRT_589	0	0	LTRRT_589	21	744.39049
LTRRT_590	0	0	LTRRT_590	0	0
LTRRT_591	0	0	LTRRT_591	0	0
LTRRT_592	0	0	LTRRT_592	0	0
LTRRT_593	0	0	LTRRT_593	0	0
LTRRT_594	3	296.61855	LTRRT_594	38	1346.9923
LTRRT_595	0	0	LTRRT_595	26	921.62632
LTRRT_596	0	0	LTRRT_596	0	0
LTRRT_597	0	0	LTRRT_597	0	0
LTRRT_598	1	98.87285	LTRRT_598	0	0
LTRRT_599	0	0	LTRRT_599	0	0
LTRRT_600	0	0	LTRRT_600	22	779.83765
LTRRT_601	3	296.61855	LTRRT_601	5	177.23583
LTRRT_602	1	98.87285	LTRRT_602	0	0
LTRRT_603	0	0	LTRRT_603	0	0
LTRRT_604	0	0	LTRRT_604	0	0
LTRRT_605	0	0	LTRRT_605	11	389.91883
LTRRT_606	6	593.2371	LTRRT_606	0	0
LTRRT_607	0	0	LTRRT_607	0	0
LTRRT_608	1	98.87285	LTRRT_608	0	0
LTRRT_609	0	0	LTRRT_609	25	886.17915
LTRRT_610	0	0	LTRRT_610	0	0
LTRRT_611	0	0	LTRRT_611	471	16695.615
LTRRT_612	0	0	LTRRT_612	0	0

LTRRT_613	1	98.87285	LTRRT_613	0	0
LTRRT_614	0	0	LTRRT_614	0	0
LTRRT_615	3	296.61855	LTRRT_615	0	0
LTRRT_616	0	0	LTRRT_616	0	0
LTRRT_617	2	197.7457	LTRRT_617	0	0
LTRRT_618	0	0	LTRRT_618	6	212.683
LTRRT_619	0	0	LTRRT_619	0	0
LTRRT_620	2	197.7457	LTRRT_620	157	5565.2051
LTRRT_621	0	0	LTRRT_621	29	1027.9678
LTRRT_622	0	0	LTRRT_622	0	0
LTRRT_623	0	0	LTRRT_623	0	0
LTRRT_624	0	0	LTRRT_624	0	0
LTRRT_625	0	0	LTRRT_625	0	0
LTRRT_626	0	0	LTRRT_626	0	0
LTRRT_627	0	0	LTRRT_627	0	0
LTRRT_628	0	0	LTRRT_628	1	35.447166
LTRRT_629	0	0	LTRRT_629	0	0
LTRRT_630	3	296.61855	LTRRT_630	0	0
LTRRT_631	0	0	LTRRT_631	10	354.47166
LTRRT_632	0	0	LTRRT_632	0	0
LTRRT_633	4	395.4914	LTRRT_633	2	70.894332
LTRRT_634	0	0	LTRRT_634	2	70.894332
LTRRT_635	5	494.36425	LTRRT_635	122	4324.5543
LTRRT_636	13	1285.347	LTRRT_636	0	0
LTRRT_637	0	0	LTRRT_637	0	0
LTRRT_638	0	0	LTRRT_638	0	0
LTRRT_639	0	0	LTRRT_639	0	0
LTRRT_640	0	0	LTRRT_640	0	0
LTRRT_641	0	0	LTRRT_641	0	0
LTRRT_642	1	98.87285	LTRRT_642	1	35.447166
LTRRT_643	0	0	LTRRT_643	0	0
LTRRT_644	0	0	LTRRT_644	33	1169.7565
LTRRT_645	0	0	LTRRT_645	0	0
LTRRT_646	0	0	LTRRT_646	0	0
LTRRT_647	0	0	LTRRT_647	0	0
LTRRT_648	0	0	LTRRT_648	0	0
LTRRT_649	0	0	LTRRT_649	0	0
LTRRT_650	13	1285.347	LTRRT_650	0	0
LTRRT_651	0	0	LTRRT_651	2	70.894332
LTRRT_652	6	593.2371	LTRRT_652	0	0
LTRRT_653	0	0	LTRRT_653	0	0
LTRRT_654	2	197.7457	LTRRT_654	12	425.36599
LTRRT_655	0	0	LTRRT_655	0	0
LTRRT_656	2	197.7457	LTRRT_656	0	0

LTRRT_657	0	0	LTRRT_657	4	141.78866
LTRRT_658	18	1779.7113	LTRRT_658	0	0
LTRRT_659	0	0	LTRRT_659	0	0
LTRRT_660	0	0	LTRRT_660	0	0
LTRRT_661	0	0	LTRRT_661	18	638.04899
LTRRT_662	0	0	LTRRT_662	3	106.3415
LTRRT_663	0	0	LTRRT_663	0	0
LTRRT_664	0	0	LTRRT_664	0	0
LTRRT_665	0	0	LTRRT_665	0	0
LTRRT_666	0	0	LTRRT_666	0	0
LTRRT_667	4	395.4914	LTRRT_667	5	177.23583
LTRRT_668	0	0	LTRRT_668	0	0
LTRRT_669	0	0	LTRRT_669	0	0
LTRRT_670	0	0	LTRRT_670	18	638.04899
LTRRT_671	1	98.87285	LTRRT_671	0	0
LTRRT_672	1	98.87285	LTRRT_672	0	0
LTRRT_673	0	0	LTRRT_673	0	0
LTRRT_674	0	0	LTRRT_674	200	7089.4332
LTRRT_675	0	0	LTRRT_675	0	0
LTRRT_676	2	197.7457	LTRRT_676	5	177.23583
LTRRT_677	0	0	LTRRT_677	0	0
LTRRT_678	0	0	LTRRT_678	0	0
LTRRT_679	2	197.7457	LTRRT_679	0	0
LTRRT_680	0	0	LTRRT_680	0	0
LTRRT_681	1	98.87285	LTRRT_681	0	0
LTRRT_682	0	0	LTRRT_682	0	0
LTRRT_683	1	98.87285	LTRRT_683	2	70.894332
LTRRT_684	0	0	LTRRT_684	0	0
LTRRT_685	0	0	LTRRT_685	0	0
LTRRT_686	18	1779.7113	LTRRT_686	0	0
LTRRT_687	12	1186.4742	LTRRT_687	0	0
LTRRT_688	0	0	LTRRT_688	0	0
LTRRT_689	0	0	LTRRT_689	0	0
LTRRT_690	0	0	LTRRT_690	0	0
LTRRT_691	0	0	LTRRT_691	0	0
LTRRT_692	15	1483.0927	LTRRT_692	0	0
LTRRT_693	0	0	LTRRT_693	0	0
LTRRT_694	0	0	LTRRT_694	1	35.447166
LTRRT_695	0	0	LTRRT_695	0	0
LTRRT_696	1	98.87285	LTRRT_696	0	0
LTRRT_697	0	0	LTRRT_697	0	0
LTRRT_698	6	593.2371	LTRRT_698	28	992.52065
LTRRT_699	0	0	LTRRT_699	0	0
LTRRT_700	0	0	LTRRT_700	0	0

LTRRT_701	1	98.87285	LTRRT_701	0	0
LTRRT_702	13	1285.347	LTRRT_702	1	35.447166
LTRRT_703	5	494.36425	LTRRT_703	2	70.894332
LTRRT_704	0	0	LTRRT_704	97	3438.3751
LTRRT_705	17	1680.8384	LTRRT_705	0	0
LTRRT_706	1	98.87285	LTRRT_706	0	0
LTRRT_707	3	296.61855	LTRRT_707	0	0
LTRRT_708	1	98.87285	LTRRT_708	0	0
LTRRT_709	4	395.4914	LTRRT_709	1	35.447166
LTRRT_710	8	790.9828	LTRRT_710	0	0
LTRRT_711	0	0	LTRRT_711	2	70.894332
LTRRT_712	0	0	LTRRT_712	2	70.894332
LTRRT_713	0	0	LTRRT_713	0	0
LTRRT_714	1	98.87285	LTRRT_714	7	248.13016
LTRRT_715	0	0	LTRRT_715	0	0
LTRRT_716	0	0	LTRRT_716	5	177.23583
LTRRT_717	0	0	LTRRT_717	3	106.3415
LTRRT_718	0	0	LTRRT_718	0	0
LTRRT_719	0	0	LTRRT_719	0	0
LTRRT_720	16	1581.9656	LTRRT_720	0	0
LTRRT_721	0	0	LTRRT_721	0	0
LTRRT_722	0	0	LTRRT_722	0	0
LTRRT_723	0	0	LTRRT_723	6	212.683
LTRRT_724	0	0	LTRRT_724	0	0
LTRRT_725	0	0	LTRRT_725	9	319.02449
LTRRT_726	0	0	LTRRT_726	0	0
LTRRT_727	0	0	LTRRT_727	1	35.447166
LTRRT_728	0	0	LTRRT_728	2	70.894332
LTRRT_729	5	494.36425	LTRRT_729	1	35.447166
LTRRT_730	0	0	LTRRT_730	0	0
LTRRT_731	1	98.87285	LTRRT_731	0	0
LTRRT_732	16	1581.9656	LTRRT_732	13	460.81316
LTRRT_733	0	0	LTRRT_733	17	602.60182
LTRRT_734	0	0	LTRRT_734	0	0
LTRRT_735	0	0	LTRRT_735	0	0
LTRRT_736	0	0	LTRRT_736	0	0
LTRRT_737	0	0	LTRRT_737	0	0
LTRRT_738	0	0	LTRRT_738	0	0
LTRRT_739	0	0	LTRRT_739	43	1524.2281
LTRRT_740	1	98.87285	LTRRT_740	0	0
LTRRT_741	0	0	LTRRT_741	0	0
LTRRT_742	0	0	LTRRT_742	1	35.447166
LTRRT_743	0	0	LTRRT_743	0	0
LTRRT_744	0	0	LTRRT_744	0	0

LTRRT_745	0	0	LTRRT_745	0	0
LTRRT_746	0	0	LTRRT_746	0	0
LTRRT_747	0	0	LTRRT_747	0	0
LTRRT_748	13	1285.347	LTRRT_748	0	0
LTRRT_749	2	197.7457	LTRRT_749	0	0
LTRRT_750	0	0	LTRRT_750	0	0
LTRRT_751	0	0	LTRRT_751	0	0
LTRRT_752	1	98.87285	LTRRT_752	0	0
LTRRT_753	0	0	LTRRT_753	2	70.894332
LTRRT_754	0	0	LTRRT_754	0	0
LTRRT_755	3	296.61855	LTRRT_755	0	0
LTRRT_756	0	0	LTRRT_756	1	35.447166
LTRRT_757	0	0	LTRRT_757	0	0
LTRRT_758	0	0	LTRRT_758	0	0
LTRRT_759	0	0	LTRRT_759	3	106.3415
LTRRT_760	0	0	LTRRT_760	0	0
LTRRT_761	0	0	LTRRT_761	0	0
LTRRT_762	0	0	LTRRT_762	0	0
LTRRT_763	0	0	LTRRT_763	0	0
LTRRT_764	1	98.87285	LTRRT_764	3	106.3415
LTRRT_765	0	0	LTRRT_765	1	35.447166
LTRRT_766	0	0	LTRRT_766	7	248.13016
LTRRT_767	0	0	LTRRT_767	2	70.894332
LTRRT_768	6	593.2371	LTRRT_768	13	460.81316
LTRRT_769	0	0	LTRRT_769	0	0
LTRRT_770	1	98.87285	LTRRT_770	6	212.683
LTRRT_771	0	0	LTRRT_771	2	70.894332
LTRRT_772	0	0	LTRRT_772	0	0
LTRRT_773	1	98.87285	LTRRT_773	1	35.447166
LTRRT_774	0	0	LTRRT_774	1	35.447166
LTRRT_775	4	395.4914	LTRRT_775	0	0
LTRRT_776	0	0	LTRRT_776	0	0
LTRRT_777	0	0	LTRRT_777	0	0
LTRRT_778	0	0	LTRRT_778	0	0
LTRRT_779	0	0	LTRRT_779	0	0
LTRRT_780	0	0	LTRRT_780	0	0
LTRRT_781	0	0	LTRRT_781	0	0
LTRRT_782	0	0	LTRRT_782	30	1063.415
LTRRT_783	0	0	LTRRT_783	0	0
LTRRT_784	0	0	LTRRT_784	0	0
LTRRT_785	0	0	LTRRT_785	0	0
LTRRT_786	0	0	LTRRT_786	0	0
LTRRT_787	0	0	LTRRT_787	13	460.81316
LTRRT_788	1	98.87285	LTRRT_788	0	0

LTRRT_789	0	0	LTRRT_789	0	0
LTRRT_790	0	0	LTRRT_790	0	0
LTRRT_791	0	0	LTRRT_791	0	0
LTRRT_792	0	0	LTRRT_792	0	0
LTRRT_793	0	0	LTRRT_793	0	0
LTRRT_794	0	0	LTRRT_794	0	0
LTRRT_795	0	0	LTRRT_795	0	0
LTRRT_796	0	0	LTRRT_796	0	0
LTRRT_797	0	0	LTRRT_797	21	744.39049
LTRRT_798	0	0	LTRRT_798	1	35.447166
LTRRT_799	0	0	LTRRT_799	0	0
LTRRT_800	0	0	LTRRT_800	0	0
LTRRT_801	1	98.87285	LTRRT_801	0	0
LTRRT_802	0	0	LTRRT_802	2	70.894332
LTRRT_803	0	0	LTRRT_803	0	0
LTRRT_804	0	0	LTRRT_804	16	567.15466
LTRRT_805	4	395.4914	LTRRT_805	0	0
LTRRT_806	0	0	LTRRT_806	6	212.683
LTRRT_807	0	0	LTRRT_807	0	0
LTRRT_808	0	0	LTRRT_808	0	0
LTRRT_809	2	197.7457	LTRRT_809	0	0
LTRRT_810	0	0	LTRRT_810	0	0
LTRRT_811	0	0	LTRRT_811	5	177.23583
LTRRT_812	0	0	LTRRT_812	0	0
LTRRT_813	0	0	LTRRT_813	4	141.78866
LTRRT_814	30	2966.1855	LTRRT_814	0	0
LTRRT_815	0	0	LTRRT_815	0	0
LTRRT_816	0	0	LTRRT_816	0	0
LTRRT_817	0	0	LTRRT_817	0	0
LTRRT_818	0	0	LTRRT_818	0	0
LTRRT_819	1	98.87285	LTRRT_819	0	0
LTRRT_820	0	0	LTRRT_820	0	0
LTRRT_821	0	0	LTRRT_821	0	0
LTRRT_822	0	0	LTRRT_822	0	0
LTRRT_823	0	0	LTRRT_823	2	70.894332
LTRRT_824	1	98.87285	LTRRT_824	0	0
LTRRT_825	0	0	LTRRT_825	5	177.23583
LTRRT_826	3	296.61855	LTRRT_826	3	106.3415
LTRRT_827	2	197.7457	LTRRT_827	0	0
LTRRT_828	0	0	LTRRT_828	0	0
LTRRT_829	0	0	LTRRT_829	0	0
LTRRT_830	4	395.4914	LTRRT_830	0	0
LTRRT_831	22	2175.2027	LTRRT_831	318	11272.199
LTRRT_832	0	0	LTRRT_832	0	0

LTRRT_833	0	0	LTRRT_833	0	0
LTRRT_834	0	0	LTRRT_834	0	0
LTRRT_835	0	0	LTRRT_835	0	0
LTRRT_836	0	0	LTRRT_836	0	0
LTRRT_837	1	98.87285	LTRRT_837	0	0
LTRRT_838	0	0	LTRRT_838	0	0
LTRRT_839	0	0	LTRRT_839	0	0
LTRRT_840	0	0	LTRRT_840	15	531.70749
LTRRT_841	0	0	LTRRT_841	0	0
LTRRT_842	0	0	LTRRT_842	3	106.3415
LTRRT_843	2384	235712.87	LTRRT_843	0	0
LTRRT_844	191	18884.714	LTRRT_844	0	0
LTRRT_845	5	494.36425	LTRRT_845	0	0
LTRRT_846	12	1186.4742	LTRRT_846	0	0
LTRRT_847	6	593.2371	LTRRT_847	1	35.447166
LTRRT_848	0	0	LTRRT_848	0	0
LTRRT_849	23	2274.0755	LTRRT_849	2	70.894332
LTRRT_850	9	889.85565	LTRRT_850	0	0
LTRRT_851	0	0	LTRRT_851	0	0
LTRRT_852	0	0	LTRRT_852	0	0
LTRRT_853	4	395.4914	LTRRT_853	0	0
LTRRT_854	0	0	LTRRT_854	0	0
LTRRT_855	0	0	LTRRT_855	0	0
LTRRT_856	0	0	LTRRT_856	3	106.3415
LTRRT_857	0	0	LTRRT_857	0	0
LTRRT_858	1	98.87285	LTRRT_858	0	0
LTRRT_859	0	0	LTRRT_859	0	0
LTRRT_860	0	0	LTRRT_860	0	0
LTRRT_861	0	0	LTRRT_861	0	0
LTRRT_862	1	98.87285	LTRRT_862	0	0
LTRRT_863	18	1779.7113	LTRRT_863	0	0
LTRRT_864	2	197.7457	LTRRT_864	2	70.894332
LTRRT_865	0	0	LTRRT_865	0	0
LTRRT_866	0	0	LTRRT_866	0	0
LTRRT_867	0	0	LTRRT_867	0	0
LTRRT_868	1	98.87285	LTRRT_868	381	13505.37
LTRRT_869	1	98.87285	LTRRT_869	55	1949.5941
LTRRT_870	0	0	LTRRT_870	40	1417.8866
LTRRT_871	0	0	LTRRT_871	1	35.447166
LTRRT_872	15	1483.0927	LTRRT_872	0	0
LTRRT_873	0	0	LTRRT_873	0	0
LTRRT_874	8	790.9828	LTRRT_874	46	1630.5696
LTRRT_875	0	0	LTRRT_875	20	708.94332
LTRRT_876	0	0	LTRRT_876	2	70.894332

LTRRT_877	0	0	LTRRT_877	0	0
LTRRT_878	0	0	LTRRT_878	0	0
LTRRT_879	0	0	LTRRT_879	4805	170323.63
LTRRT_880	0	0	LTRRT_880	8	283.57733
LTRRT_881	12	1186.4742	LTRRT_881	273	9677.0763
LTRRT_882	9	889.85565	LTRRT_882	0	0
LTRRT_883	0	0	LTRRT_883	0	0
LTRRT_884	0	0	LTRRT_884	2	70.894332
LTRRT_885	24	2372.9484	LTRRT_885	23	815.28482
LTRRT_886	0	0	LTRRT_886	28	992.52065
LTRRT_887	2	197.7457	LTRRT_887	21	744.39049
LTRRT_888	0	0	LTRRT_888	0	0
LTRRT_889	0	0	LTRRT_889	14	496.26032
LTRRT_890	0	0	LTRRT_890	4	141.78866
LTRRT_891	0	0	LTRRT_891	0	0
LTRRT_892	0	0	LTRRT_892	0	0
LTRRT_893	0	0	LTRRT_893	1	35.447166
LTRRT_894	0	0	LTRRT_894	13	460.81316
LTRRT_895	0	0	LTRRT_895	12	425.36599
LTRRT_896	0	0	LTRRT_896	0	0
LTRRT_897	1	98.87285	LTRRT_897	205	7266.669
LTRRT_898	0	0	LTRRT_898	0	0
LTRRT_899	1	98.87285	LTRRT_899	0	0
LTRRT_900	0	0	LTRRT_900	0	0
LTRRT_901	0	0	LTRRT_901	0	0
LTRRT_902	0	0	LTRRT_902	2	70.894332
LTRRT_903	0	0	LTRRT_903	0	0
LTRRT_904	0	0	LTRRT_904	0	0
LTRRT_905	0	0	LTRRT_905	0	0
LTRRT_906	1	98.87285	LTRRT_906	0	0
LTRRT_907	3	296.61855	LTRRT_907	0	0
LTRRT_908	0	0	LTRRT_908	340	12052.036
LTRRT_909	0	0	LTRRT_909	91	3225.6921
LTRRT_910	1	98.87285	LTRRT_910	31	1098.8621
LTRRT_911	0	0	LTRRT_911	3	106.3415
LTRRT_912	0	0	LTRRT_912	322	11413.987
LTRRT_913	1	98.87285	LTRRT_913	0	0
LTRRT_914	0	0	LTRRT_914	0	0
LTRRT_915	4	395.4914	LTRRT_915	1	35.447166
LTRRT_916	0	0	LTRRT_916	0	0
LTRRT_917	0	0	LTRRT_917	0	0
LTRRT_918	0	0	LTRRT_918	2	70.894332
LTRRT_919	0	0	LTRRT_919	0	0
LTRRT_920	0	0	LTRRT_920	0	0

LTRRT_921	3	296.61855	LTRRT_921	0	0
LTRRT_922	0	0	LTRRT_922	0	0
LTRRT_923	0	0	LTRRT_923	30	1063.415
LTRRT_924	1	98.87285	LTRRT_924	0	0
LTRRT_925	0	0	LTRRT_925	0	0
LTRRT_926	0	0	LTRRT_926	0	0
LTRRT_927	0	0	LTRRT_927	21	744.39049
LTRRT_928	0	0	LTRRT_928	0	0
LTRRT_929	0	0	LTRRT_929	3	106.3415
LTRRT_930	0	0	LTRRT_930	0	0
LTRRT_931	0	0	LTRRT_931	1	35.447166
LTRRT_932	0	0	LTRRT_932	0	0
LTRRT_933	2	197.7457	LTRRT_933	519	18397.079
LTRRT_934	0	0	LTRRT_934	0	0
LTRRT_935	1	98.87285	LTRRT_935	0	0
LTRRT_936	0	0	LTRRT_936	0	0
LTRRT_937	0	0	LTRRT_937	1	35.447166
LTRRT_938	2	197.7457	LTRRT_938	0	0
LTRRT_939	3	296.61855	LTRRT_939	58	2055.9356
LTRRT_940	0	0	LTRRT_940	189	6699.5144
LTRRT_941	0	0	LTRRT_941	0	0
LTRRT_942	0	0	LTRRT_942	434	15384.07
LTRRT_943	0	0	LTRRT_943	0	0
LTRRT_944	1	98.87285	LTRRT_944	0	0
LTRRT_945	1	98.87285	LTRRT_945	0	0
LTRRT_946	0	0	LTRRT_946	0	0
LTRRT_947	0	0	LTRRT_947	8	283.57733
LTRRT_948	0	0	LTRRT_948	0	0
LTRRT_949	2	197.7457	LTRRT_949	11	389.91883
LTRRT_950	0	0	LTRRT_950	0	0
LTRRT_951	1	98.87285	LTRRT_951	1	35.447166
LTRRT_952	0	0	LTRRT_952	0	0
LTRRT_953	0	0	LTRRT_953	0	0
LTRRT_954	4	395.4914	LTRRT_954	0	0
LTRRT_955	62	6130.1167	LTRRT_955	0	0
LTRRT_956	0	0	LTRRT_956	0	0
LTRRT_957	0	0	LTRRT_957	0	0
LTRRT_958	0	0	LTRRT_958	0	0
LTRRT_959	47	4647.0239	LTRRT_959	0	0
LTRRT_960	1	98.87285	LTRRT_960	0	0
LTRRT_961	0	0	LTRRT_961	1	35.447166
LTRRT_962	8	790.9828	LTRRT_962	0	0
LTRRT_963	0	0	LTRRT_963	0	0
LTRRT_964	14	1384.2199	LTRRT_964	57	2020.4885

LTRRT_965	0	0	LTRRT_965	0	0
LTRRT_966	32	3163.9312	LTRRT_966	0	0
LTRRT_967	0	0	LTRRT_967	1	35.447166
LTRRT_968	0	0	LTRRT_968	4	141.78866
LTRRT_969	0	0	LTRRT_969	1	35.447166
LTRRT_970	0	0	LTRRT_970	0	0
LTRRT_971	0	0	LTRRT_971	0	0
LTRRT_972	0	0	LTRRT_972	2	70.894332
LTRRT_973	0	0	LTRRT_973	0	0
LTRRT_974	0	0	LTRRT_974	0	0
LTRRT_975	0	0	LTRRT_975	0	0
LTRRT_976	1	98.87285	LTRRT_976	22	779.83765
LTRRT_977	0	0	LTRRT_977	0	0
LTRRT_978	0	0	LTRRT_978	4	141.78866
LTRRT_979	0	0	LTRRT_979	0	0
LTRRT_980	1	98.87285	LTRRT_980	0	0
LTRRT_981	0	0	LTRRT_981	0	0
LTRRT_982	0	0	LTRRT_982	0	0
LTRRT_983	2	197.7457	LTRRT_983	41	1453.3338
LTRRT_984	0	0	LTRRT_984	0	0
LTRRT_985	0	0	LTRRT_985	0	0
LTRRT_986	0	0	LTRRT_986	14	496.26032
LTRRT_987	0	0	LTRRT_987	0	0
LTRRT_988	0	0	LTRRT_988	0	0
LTRRT_989	0	0	LTRRT_989	11	389.91883
LTRRT_990	5	494.36425	LTRRT_990	1	35.447166
LTRRT_991	0	0	LTRRT_991	0	0
LTRRT_992	0	0	LTRRT_992	0	0
LTRRT_993	0	0	LTRRT_993	3	106.3415
LTRRT_994	3	296.61855	LTRRT_994	2	70.894332
LTRRT_995	3	296.61855	LTRRT_995	0	0
LTRRT_996	0	0	LTRRT_996	0	0
LTRRT_997	0	0	LTRRT_997	0	0
LTRRT_998	0	0	LTRRT_998	27	957.07348
LTRRT_999	0	0	LTRRT_999	0	0
LTRRT_1000	0	0	LTRRT_1000	0	0
LTRRT_1001	1	98.87285	LTRRT_1001	0	0
LTRRT_1002	0	0	LTRRT_1002	0	0
LTRRT_1003	0	0	LTRRT_1003	0	0
LTRRT_1004	0	0	LTRRT_1004	0	0
LTRRT_1005	0	0	LTRRT_1005	1	35.447166
LTRRT_1006	0	0	LTRRT_1006	0	0
LTRRT_1007	0	0	LTRRT_1007	0	0
LTRRT_1008	1	98.87285	LTRRT_1008	0	0

LTRRT_1009	0	0	LTRRT_1009	34	1205.2036
LTRRT_1010	0	0	LTRRT_1010	0	0
LTRRT_1011	0	0	LTRRT_1011	0	0
LTRRT_1012	0	0	LTRRT_1012	0	0
LTRRT_1013	0	0	LTRRT_1013	0	0
LTRRT_1014	0	0	LTRRT_1014	0	0
LTRRT_1015	4	395.4914	LTRRT_1015	0	0
LTRRT_1016	0	0	LTRRT_1016	0	0
LTRRT_1017	0	0	LTRRT_1017	0	0
LTRRT_1018	0	0	LTRRT_1018	0	0
LTRRT_1019	0	0	LTRRT_1019	61	2162.2771
LTRRT_1020	0	0	LTRRT_1020	0	0
LTRRT_1021	0	0	LTRRT_1021	2	70.894332
LTRRT_1022	0	0	LTRRT_1022	0	0
LTRRT_1023	0	0	LTRRT_1023	0	0
LTRRT_1024	0	0	LTRRT_1024	0	0
LTRRT_1025	0	0	LTRRT_1025	0	0
LTRRT_1026	6	593.2371	LTRRT_1026	0	0
LTRRT_1027	0	0	LTRRT_1027	0	0
LTRRT_1028	0	0	LTRRT_1028	0	0
LTRRT_1029	0	0	LTRRT_1029	0	0
LTRRT_1030	0	0	LTRRT_1030	0	0
LTRRT_1031	0	0	LTRRT_1031	3	106.3415
LTRRT_1032	0	0	LTRRT_1032	5	177.23583
LTRRT_1033	0	0	LTRRT_1033	1211	42926.518
LTRRT_1034	0	0	LTRRT_1034	0	0
LTRRT_1035	13	1285.347	LTRRT_1035	41	1453.3338
LTRRT_1036	77	7613.2094	LTRRT_1036	1	35.447166
LTRRT_1037	8	790.9828	LTRRT_1037	0	0
LTRRT_1038	0	0	LTRRT_1038	2	70.894332
LTRRT_1039	0	0	LTRRT_1039	0	0
LTRRT_1040	0	0	LTRRT_1040	0	0
LTRRT_1041	3	296.61855	LTRRT_1041	0	0
LTRRT_1042	3	296.61855	LTRRT_1042	0	0
LTRRT_1043	0	0	LTRRT_1043	0	0
LTRRT_1044	0	0	LTRRT_1044	0	0
LTRRT_1045	0	0	LTRRT_1045	4	141.78866
LTRRT_1046	0	0	LTRRT_1046	0	0
LTRRT_1047	0	0	LTRRT_1047	0	0
LTRRT_1048	0	0	LTRRT_1048	0	0
LTRRT_1049	0	0	LTRRT_1049	133	4714.4731
LTRRT_1050	0	0	LTRRT_1050	0	0
LTRRT_1051	0	0	LTRRT_1051	0	0
LTRRT_1052	1	98.87285	LTRRT_1052	0	0

LTRRT_1053	21	2076.3298	LTRRT_1053	8	283.57733
LTRRT_1054	0	0	LTRRT_1054	313	11094.963
LTRRT_1055	0	0	LTRRT_1055	0	0
LTRRT_1056	19	1878.5841	LTRRT_1056	0	0
LTRRT_1057	17	1680.8384	LTRRT_1057	0	0
LTRRT_1058	7	692.10995	LTRRT_1058	1	35.447166
LTRRT_1059	0	0	LTRRT_1059	0	0
LTRRT_1060	0	0	LTRRT_1060	0	0
LTRRT_1061	0	0	LTRRT_1061	0	0
LTRRT_1062	2	197.7457	LTRRT_1062	0	0
LTRRT_1063	0	0	LTRRT_1063	0	0
LTRRT_1064	2	197.7457	LTRRT_1064	0	0
LTRRT_1065	0	0	LTRRT_1065	2	70.894332
LTRRT_1066	0	0	LTRRT_1066	0	0
LTRRT_1067	3	296.61855	LTRRT_1067	1	35.447166
LTRRT_1068	0	0	LTRRT_1068	318	11272.199
LTRRT_1069	0	0	LTRRT_1069	0	0
LTRRT_1070	0	0	LTRRT_1070	14	496.26032
LTRRT_1071	0	0	LTRRT_1071	3	106.3415
LTRRT_1072	0	0	LTRRT_1072	1	35.447166
LTRRT_1073	0	0	LTRRT_1073	0	0
LTRRT_1074	0	0	LTRRT_1074	0	0
LTRRT_1075	0	0	LTRRT_1075	0	0
LTRRT_1076	0	0	LTRRT_1076	134	4749.9202
LTRRT_1077	0	0	LTRRT_1077	0	0
LTRRT_1078	0	0	LTRRT_1078	0	0
LTRRT_1079	20	1977.457	LTRRT_1079	3	106.3415
LTRRT_1080	0	0	LTRRT_1080	1	35.447166
LTRRT_1081	9	889.85565	LTRRT_1081	0	0
LTRRT_1082	2	197.7457	LTRRT_1082	0	0
LTRRT_1083	0	0	LTRRT_1083	0	0
LTRRT_1084	41	4053.7868	LTRRT_1084	22	779.83765
LTRRT_1085	0	0	LTRRT_1085	0	0
LTRRT_1086	10	988.7285	LTRRT_1086	0	0
LTRRT_1087	0	0	LTRRT_1087	0	0
LTRRT_1088	5	494.36425	LTRRT_1088	0	0
LTRRT_1089	5	494.36425	LTRRT_1089	0	0
LTRRT_1090	0	0	LTRRT_1090	0	0
LTRRT_1091	13	1285.347	LTRRT_1091	0	0
LTRRT_1092	1	98.87285	LTRRT_1092	0	0
LTRRT_1093	0	0	LTRRT_1093	2	70.894332
LTRRT_1094	0	0	LTRRT_1094	5	177.23583
LTRRT_1095	0	0	LTRRT_1095	0	0
LTRRT_1096	23	2274.0755	LTRRT_1096	0	0

LTRRT_1097	0	0	LTRRT_1097	0	0
LTRRT_1098	0	0	LTRRT_1098	0	0
LTRRT_1099	4	395.4914	LTRRT_1099	16	567.15466
LTRRT_1100	0	0	LTRRT_1100	1	35.447166
LTRRT_1101	2	197.7457	LTRRT_1101	0	0
LTRRT_1102	0	0	LTRRT_1102	0	0
LTRRT_1103	0	0	LTRRT_1103	0	0
LTRRT_1104	0	0	LTRRT_1104	0	0
LTRRT_1105	0	0	LTRRT_1105	9	319.02449
LTRRT_1106	0	0	LTRRT_1106	0	0
LTRRT_1107	0	0	LTRRT_1107	2	70.894332
LTRRT_1108	5	494.36425	LTRRT_1108	0	0
LTRRT_1109	0	0	LTRRT_1109	0	0
LTRRT_1110	0	0	LTRRT_1110	0	0
LTRRT_1111	0	0	LTRRT_1111	8	283.57733
LTRRT_1112	23	2274.0755	LTRRT_1112	45	1595.1225
LTRRT_1113	0	0	LTRRT_1113	0	0
LTRRT_1114	0	0	LTRRT_1114	0	0
LTRRT_1115	0	0	LTRRT_1115	0	0
LTRRT_1116	0	0	LTRRT_1116	1	35.447166
LTRRT_1117	0	0	LTRRT_1117	0	0
LTRRT_1118	0	0	LTRRT_1118	0	0
LTRRT_1119	0	0	LTRRT_1119	37	1311.5451
LTRRT_1120	0	0	LTRRT_1120	43	1524.2281
LTRRT_1121	0	0	LTRRT_1121	9	319.02449
LTRRT_1122	0	0	LTRRT_1122	65	2304.0658
LTRRT_1123	0	0	LTRRT_1123	106	3757.3996
LTRRT_1124	0	0	LTRRT_1124	0	0
LTRRT_1125	0	0	LTRRT_1125	0	0
LTRRT_1126	0	0	LTRRT_1126	0	0
LTRRT_1127	0	0	LTRRT_1127	0	0
LTRRT_1128	0	0	LTRRT_1128	0	0
LTRRT_1129	2	197.7457	LTRRT_1129	0	0
LTRRT_1130	0	0	LTRRT_1130	18	638.04899
LTRRT_1131	0	0	LTRRT_1131	0	0
LTRRT_1132	0	0	LTRRT_1132	0	0
LTRRT_1133	1	98.87285	LTRRT_1133	0	0
LTRRT_1134	0	0	LTRRT_1134	7	248.13016
LTRRT_1135	0	0	LTRRT_1135	1	35.447166
LTRRT_1136	0	0	LTRRT_1136	0	0
LTRRT_1137	0	0	LTRRT_1137	0	0
LTRRT_1138	5	494.36425	LTRRT_1138	0	0
LTRRT_1139	0	0	LTRRT_1139	6	212.683
LTRRT_1140	0	0	LTRRT_1140	0	0

LTRRT_1141	0	0	LTRRT_1141	0	0
LTRRT_1142	2	197.7457	LTRRT_1142	1	35.447166
LTRRT_1143	0	0	LTRRT_1143	0	0
LTRRT_1144	0	0	LTRRT_1144	0	0
LTRRT_1145	0	0	LTRRT_1145	4	141.78866
LTRRT_1146	0	0	LTRRT_1146	33	1169.7565
LTRRT_1147	0	0	LTRRT_1147	1	35.447166
LTRRT_1148	0	0	LTRRT_1148	2	70.894332
LTRRT_1149	0	0	LTRRT_1149	0	0
LTRRT_1150	0	0	LTRRT_1150	195	6912.1974
LTRRT_1151	0	0	LTRRT_1151	0	0
LTRRT_1152	10	988.7285	LTRRT_1152	0	0
LTRRT_1153	0	0	LTRRT_1153	1	35.447166
LTRRT_1154	0	0	LTRRT_1154	60	2126.83
LTRRT_1155	2	197.7457	LTRRT_1155	2	70.894332
LTRRT_1156	0	0	LTRRT_1156	0	0
LTRRT_1157	0	0	LTRRT_1157	0	0
LTRRT_1158	0	0	LTRRT_1158	0	0
LTRRT_1159	0	0	LTRRT_1159	0	0
LTRRT_1160	0	0	LTRRT_1160	0	0
LTRRT_1161	4	395.4914	LTRRT_1161	11	389.91883
LTRRT_1162	1	98.87285	LTRRT_1162	0	0
LTRRT_1163	0	0	LTRRT_1163	5	177.23583
LTRRT_1164	9	889.85565	LTRRT_1164	51	1807.8055
LTRRT_1165	0	0	LTRRT_1165	2	70.894332
LTRRT_1166	0	0	LTRRT_1166	0	0
LTRRT_1167	0	0	LTRRT_1167	0	0
LTRRT_1168	0	0	LTRRT_1168	0	0
LTRRT_1169	0	0	LTRRT_1169	0	0
LTRRT_1170	0	0	LTRRT_1170	3	106.3415
LTRRT_1171	0	0	LTRRT_1171	0	0
LTRRT_1172	0	0	LTRRT_1172	0	0
LTRRT_1173	0	0	LTRRT_1173	0	0
LTRRT_1174	0	0	LTRRT_1174	0	0
LTRRT_1175	0	0	LTRRT_1175	0	0
LTRRT_1176	0	0	LTRRT_1176	0	0
LTRRT_1177	7	692.10995	LTRRT_1177	0	0
LTRRT_1178	0	0	LTRRT_1178	9	319.02449
LTRRT_1179	0	0	LTRRT_1179	7	248.13016
LTRRT_1180	0	0	LTRRT_1180	82	2906.6676
LTRRT_1181	0	0	LTRRT_1181	1	35.447166
LTRRT_1182	0	0	LTRRT_1182	0	0
LTRRT_1183	4	395.4914	LTRRT_1183	0	0
LTRRT_1184	8	790.9828	LTRRT_1184	0	0

LTRRT_1185	0	0	LTRRT_1185	0	0
LTRRT_1186	0	0	LTRRT_1186	0	0
LTRRT_1187	7	692.10995	LTRRT_1187	0	0
LTRRT_1188	0	0	LTRRT_1188	1	35.447166
LTRRT_1189	0	0	LTRRT_1189	0	0
LTRRT_1190	115	11370.378	LTRRT_1190	0	0
LTRRT_1191	0	0	LTRRT_1191	0	0
LTRRT_1192	0	0	LTRRT_1192	0	0
LTRRT_1193	0	0	LTRRT_1193	0	0
LTRRT_1194	0	0	LTRRT_1194	99	3509.2694
LTRRT_1195	1	98.87285	LTRRT_1195	0	0
LTRRT_1196	0	0	LTRRT_1196	0	0
LTRRT_1197	0	0	LTRRT_1197	0	0
LTRRT_1198	0	0	LTRRT_1198	23	815.28482
LTRRT_1199	2	197.7457	LTRRT_1199	0	0
LTRRT_1200	0	0	LTRRT_1200	0	0
LTRRT_1201	0	0	LTRRT_1201	92	3261.1393
LTRRT_1202	5	494.36425	LTRRT_1202	12	425.36599
LTRRT_1203	5	494.36425	LTRRT_1203	17	602.60182
LTRRT_1204	0	0	LTRRT_1204	0	0
LTRRT_1205	0	0	LTRRT_1205	0	0
LTRRT_1206	0	0	LTRRT_1206	0	0
LTRRT_1207	0	0	LTRRT_1207	6	212.683
LTRRT_1208	0	0	LTRRT_1208	0	0
LTRRT_1209	0	0	LTRRT_1209	0	0
LTRRT_1210	0	0	LTRRT_1210	0	0
LTRRT_1211	0	0	LTRRT_1211	0	0
LTRRT_1212	5	494.36425	LTRRT_1212	0	0
LTRRT_1213	0	0	LTRRT_1213	36	1276.098
LTRRT_1214	0	0	LTRRT_1214	160	5671.5466
LTRRT_1215	0	0	LTRRT_1215	0	0
LTRRT_1216	0	0	LTRRT_1216	14	496.26032
LTRRT_1217	0	0	LTRRT_1217	0	0
LTRRT_1218	0	0	LTRRT_1218	0	0
LTRRT_1219	0	0	LTRRT_1219	0	0
LTRRT_1220	0	0	LTRRT_1220	0	0
LTRRT_1221	0	0	LTRRT_1221	0	0
LTRRT_1222	0	0	LTRRT_1222	1	35.447166
LTRRT_1223	0	0	LTRRT_1223	0	0
LTRRT_1224	0	0	LTRRT_1224	0	0
LTRRT_1225	0	0	LTRRT_1225	0	0
LTRRT_1226	3	296.61855	LTRRT_1226	0	0
LTRRT_1227	0	0	LTRRT_1227	0	0
LTRRT_1228	0	0	LTRRT_1228	0	0

LTRRT_1229	18	1779.7113	LTRRT_1229	0	0
LTRRT_1230	8	790.9828	LTRRT_1230	1	35.447166
LTRRT_1231	0	0	LTRRT_1231	0	0
LTRRT_1232	5	494.36425	LTRRT_1232	0	0
LTRRT_1233	0	0	LTRRT_1233	15	531.70749
LTRRT_1234	0	0	LTRRT_1234	2	70.894332
LTRRT_1235	0	0	LTRRT_1235	7	248.13016
LTRRT_1236	0	0	LTRRT_1236	18	638.04899
LTRRT_1237	0	0	LTRRT_1237	0	0
LTRRT_1238	0	0	LTRRT_1238	10	354.47166
LTRRT_1239	0	0	LTRRT_1239	0	0
LTRRT_1240	0	0	LTRRT_1240	0	0
LTRRT_1241	0	0	LTRRT_1241	0	0
LTRRT_1242	0	0	LTRRT_1242	0	0
LTRRT_1243	0	0	LTRRT_1243	19	673.49615
LTRRT_1244	0	0	LTRRT_1244	0	0
LTRRT_1245	0	0	LTRRT_1245	0	0
LTRRT_1246	0	0	LTRRT_1246	0	0
LTRRT_1247	0	0	LTRRT_1247	0	0
LTRRT_1248	0	0	LTRRT_1248	0	0
LTRRT_1249	0	0	LTRRT_1249	0	0
LTRRT_1250	2	197.7457	LTRRT_1250	3	106.3415
LTRRT_1251	0	0	LTRRT_1251	0	0
LTRRT_1252	0	0	LTRRT_1252	0	0
LTRRT_1253	0	0	LTRRT_1253	0	0
LTRRT_1254	0	0	LTRRT_1254	0	0
LTRRT_1255	0	0	LTRRT_1255	0	0
LTRRT_1256	0	0	LTRRT_1256	0	0
LTRRT_1257	1	98.87285	LTRRT_1257	0	0
LTRRT_1258	0	0	LTRRT_1258	0	0
LTRRT_1259	0	0	LTRRT_1259	0	0
LTRRT_1260	1	98.87285	LTRRT_1260	20	708.94332
LTRRT_1261	0	0	LTRRT_1261	34	1205.2036
LTRRT_1262	1	98.87285	LTRRT_1262	0	0
LTRRT_1263	2	197.7457	LTRRT_1263	0	0
LTRRT_1264	0	0	LTRRT_1264	0	0
LTRRT_1265	0	0	LTRRT_1265	0	0
LTRRT_1266	0	0	LTRRT_1266	1	35.447166
LTRRT_1267	0	0	LTRRT_1267	0	0
LTRRT_1268	0	0	LTRRT_1268	2	70.894332
LTRRT_1269	0	0	LTRRT_1269	0	0
LTRRT_1270	0	0	LTRRT_1270	0	0
LTRRT_1271	0	0	LTRRT_1271	10	354.47166
LTRRT_1272	0	0	LTRRT_1272	0	0

LTRRT_1273	14	1384.2199	LTRRT_1273	0	0
LTRRT_1274	0	0	LTRRT_1274	0	0
LTRRT_1275	0	0	LTRRT_1275	2	70.894332
LTRRT_1276	0	0	LTRRT_1276	0	0
LTRRT_1277	0	0	LTRRT_1277	9	319.02449
LTRRT_1278	0	0	LTRRT_1278	1	35.447166
LTRRT_1279	0	0	LTRRT_1279	0	0
LTRRT_1280	0	0	LTRRT_1280	0	0
LTRRT_1281	0	0	LTRRT_1281	46	1630.5696
LTRRT_1282	0	0	LTRRT_1282	73	2587.6431
LTRRT_1283	1	98.87285	LTRRT_1283	0	0
LTRRT_1284	0	0	LTRRT_1284	0	0
LTRRT_1285	0	0	LTRRT_1285	354	12548.297
LTRRT_1286	0	0	LTRRT_1286	0	0
LTRRT_1287	17	1680.8384	LTRRT_1287	1	35.447166
LTRRT_1288	0	0	LTRRT_1288	0	0
LTRRT_1289	0	0	LTRRT_1289	0	0
LTRRT_1290	1	98.87285	LTRRT_1290	15	531.70749
LTRRT_1291	0	0	LTRRT_1291	0	0
LTRRT_1292	0	0	LTRRT_1292	0	0
LTRRT_1293	2	197.7457	LTRRT_1293	3	106.3415
LTRRT_1294	2	197.7457	LTRRT_1294	1	35.447166
LTRRT_1295	2	197.7457	LTRRT_1295	3	106.3415
LTRRT_1296	9	889.85565	LTRRT_1296	0	0
LTRRT_1297	0	0	LTRRT_1297	0	0
LTRRT_1298	0	0	LTRRT_1298	679	24068.626
LTRRT_1299	0	0	LTRRT_1299	8	283.57733
LTRRT_1300	4	395.4914	LTRRT_1300	25	886.17915
LTRRT_1301	0	0	LTRRT_1301	0	0
LTRRT_1302	0	0	LTRRT_1302	0	0
LTRRT_1303	0	0	LTRRT_1303	0	0
LTRRT_1304	0	0	LTRRT_1304	0	0
LTRRT_1305	0	0	LTRRT_1305	3	106.3415
LTRRT_1306	0	0	LTRRT_1306	0	0
LTRRT_1307	13	1285.347	LTRRT_1307	0	0
LTRRT_1308	2	197.7457	LTRRT_1308	0	0
LTRRT_1309	0	0	LTRRT_1309	0	0
LTRRT_1310	0	0	LTRRT_1310	0	0
LTRRT_1311	0	0	LTRRT_1311	0	0
LTRRT_1312	0	0	LTRRT_1312	0	0
LTRRT_1313	0	0	LTRRT_1313	3	106.3415
LTRRT_1314	0	0	LTRRT_1314	0	0
LTRRT_1315	0	0	LTRRT_1315	0	0
LTRRT_1316	0	0	LTRRT_1316	0	0

LTRRT_1317	0	0	LTRRT_1317	0	0
LTRRT_1318	3	296.61855	LTRRT_1318	0	0
LTRRT_1319	0	0	LTRRT_1319	0	0
LTRRT_1320	1	98.87285	LTRRT_1320	0	0
LTRRT_1321	0	0	LTRRT_1321	0	0
LTRRT_1322	0	0	LTRRT_1322	8	283.57733
LTRRT_1323	0	0	LTRRT_1323	0	0
LTRRT_1324	3	296.61855	LTRRT_1324	221	7833.8237
LTRRT_1325	0	0	LTRRT_1325	1	35.447166
LTRRT_1326	2	197.7457	LTRRT_1326	1	35.447166
LTRRT_1327	0	0	LTRRT_1327	0	0
LTRRT_1328	0	0	LTRRT_1328	6	212.683
LTRRT_1329	0	0	LTRRT_1329	0	0
LTRRT_1330	0	0	LTRRT_1330	4	141.78866
LTRRT_1331	0	0	LTRRT_1331	0	0
LTRRT_1332	0	0	LTRRT_1332	363	12867.321
LTRRT_1333	2	197.7457	LTRRT_1333	0	0
LTRRT_1334	0	0	LTRRT_1334	0	0
LTRRT_1335	0	0	LTRRT_1335	0	0
LTRRT_1336	23	2274.0755	LTRRT_1336	11	389.91883
LTRRT_1337	1	98.87285	LTRRT_1337	0	0
LTRRT_1338	34	3361.6769	LTRRT_1338	0	0
LTRRT_1339	0	0	LTRRT_1339	257	9109.9217
LTRRT_1340	0	0	LTRRT_1340	0	0
LTRRT_1341	0	0	LTRRT_1341	0	0
LTRRT_1342	0	0	LTRRT_1342	0	0
LTRRT_1343	0	0	LTRRT_1343	0	0
LTRRT_1344	0	0	LTRRT_1344	0	0
LTRRT_1345	0	0	LTRRT_1345	0	0
LTRRT_1346	0	0	LTRRT_1346	0	0
LTRRT_1347	26	2570.6941	LTRRT_1347	0	0
LTRRT_1348	0	0	LTRRT_1348	0	0
LTRRT_1349	1	98.87285	LTRRT_1349	0	0
LTRRT_1350	2	197.7457	LTRRT_1350	23	815.28482
LTRRT_1351	13	1285.347	LTRRT_1351	0	0
LTRRT_1352	0	0	LTRRT_1352	0	0
LTRRT_1353	0	0	LTRRT_1353	0	0
LTRRT_1354	0	0	LTRRT_1354	0	0
LTRRT_1355	0	0	LTRRT_1355	214	7585.6935
LTRRT_1356	0	0	LTRRT_1356	0	0
LTRRT_1357	0	0	LTRRT_1357	0	0
LTRRT_1358	0	0	LTRRT_1358	4	141.78866
LTRRT_1359	0	0	LTRRT_1359	0	0
LTRRT_1360	0	0	LTRRT_1360	2	70.894332

LTRRT_1361	0	0	LTRRT_1361	0	0
LTRRT_1362	0	0	LTRRT_1362	0	0
LTRRT_1363	0	0	LTRRT_1363	3	106.3415
LTRRT_1364	0	0	LTRRT_1364	42	1488.781
LTRRT_1365	0	0	LTRRT_1365	0	0
LTRRT_1366	0	0	LTRRT_1366	0	0
LTRRT_1367	0	0	LTRRT_1367	0	0
LTRRT_1368	6	593.2371	LTRRT_1368	214	7585.6935
LTRRT_1369	18	1779.7113	LTRRT_1369	0	0
LTRRT_1370	1	98.87285	LTRRT_1370	1	35.447166
LTRRT_1371	3	296.61855	LTRRT_1371	0	0
LTRRT_1372	0	0	LTRRT_1372	0	0
LTRRT_1373	0	0	LTRRT_1373	0	0
LTRRT_1374	1	98.87285	LTRRT_1374	0	0
LTRRT_1375	66	6525.6081	LTRRT_1375	0	0
LTRRT_1376	0	0	LTRRT_1376	0	0
LTRRT_1377	0	0	LTRRT_1377	0	0
LTRRT_1378	0	0	LTRRT_1378	0	0
LTRRT_1379	5	494.36425	LTRRT_1379	35	1240.6508
LTRRT_1380	0	0	LTRRT_1380	0	0
LTRRT_1381	2	197.7457	LTRRT_1381	0	0
LTRRT_1382	0	0	LTRRT_1382	36	1276.098
LTRRT_1383	0	0	LTRRT_1383	0	0
LTRRT_1384	0	0	LTRRT_1384	34	1205.2036
LTRRT_1385	10	988.7285	LTRRT_1385	0	0
LTRRT_1386	0	0	LTRRT_1386	0	0
LTRRT_1387	0	0	LTRRT_1387	0	0
LTRRT_1388	0	0	LTRRT_1388	0	0
LTRRT_1389	0	0	LTRRT_1389	0	0
LTRRT_1390	0	0	LTRRT_1390	0	0
LTRRT_1391	0	0	LTRRT_1391	0	0
LTRRT_1392	0	0	LTRRT_1392	25	886.17915
LTRRT_1393	0	0	LTRRT_1393	0	0
LTRRT_1394	0	0	LTRRT_1394	0	0
LTRRT_1395	0	0	LTRRT_1395	1	35.447166
LTRRT_1396	0	0	LTRRT_1396	0	0
LTRRT_1397	0	0	LTRRT_1397	289	10244.231
LTRRT_1398	0	0	LTRRT_1398	4	141.78866
LTRRT_1399	0	0	LTRRT_1399	100	3544.7166
LTRRT_1400	0	0	LTRRT_1400	0	0
LTRRT_1401	0	0	LTRRT_1401	1	35.447166
LTRRT_1402	0	0	LTRRT_1402	19	673.49615
LTRRT_1403	0	0	LTRRT_1403	0	0
LTRRT_1404	0	0	LTRRT_1404	10	354.47166

LTRRT_1405	1	98.87285	LTRRT_1405	0	0
LTRRT_1406	0	0	LTRRT_1406	0	0
LTRRT_1407	0	0	LTRRT_1407	6	212.683
LTRRT_1408	0	0	LTRRT_1408	0	0
LTRRT_1409	0	0	LTRRT_1409	0	0
LTRRT_1410	0	0	LTRRT_1410	0	0
LTRRT_1411	17	1680.8384	LTRRT_1411	0	0
LTRRT_1412	0	0	LTRRT_1412	0	0
LTRRT_1413	4	395.4914	LTRRT_1413	0	0
LTRRT_1414	1	98.87285	LTRRT_1414	2	70.894332
LTRRT_1415	0	0	LTRRT_1415	0	0
LTRRT_1416	1	98.87285	LTRRT_1416	0	0
LTRRT_1417	0	0	LTRRT_1417	0	0
LTRRT_1418	0	0	LTRRT_1418	0	0
LTRRT_1419	0	0	LTRRT_1419	0	0
LTRRT_1420	0	0	LTRRT_1420	0	0
LTRRT_1421	0	0	LTRRT_1421	0	0
LTRRT_1422	0	0	LTRRT_1422	39	1382.4395
LTRRT_1423	0	0	LTRRT_1423	69	2445.8545
LTRRT_1424	0	0	LTRRT_1424	0	0
LTRRT_1425	0	0	LTRRT_1425	2	70.894332
LTRRT_1426	0	0	LTRRT_1426	0	0
LTRRT_1427	0	0	LTRRT_1427	0	0
LTRRT_1428	0	0	LTRRT_1428	19	673.49615
LTRRT_1429	0	0	LTRRT_1429	4	141.78866
LTRRT_1430	0	0	LTRRT_1430	0	0
LTRRT_1431	0	0	LTRRT_1431	293	10386.02
LTRRT_1432	2	197.7457	LTRRT_1432	0	0
LTRRT_1433	3	296.61855	LTRRT_1433	0	0
LTRRT_1434	0	0	LTRRT_1434	0	0
LTRRT_1435	0	0	LTRRT_1435	0	0
LTRRT_1436	0	0	LTRRT_1436	0	0
LTRRT_1437	1	98.87285	LTRRT_1437	0	0
LTRRT_1438	0	0	LTRRT_1438	11	389.91883
LTRRT_1439	5	494.36425	LTRRT_1439	0	0
LTRRT_1440	0	0	LTRRT_1440	0	0
LTRRT_1441	2	197.7457	LTRRT_1441	0	0
LTRRT_1442	15	1483.0927	LTRRT_1442	0	0
LTRRT_1443	4	395.4914	LTRRT_1443	1	35.447166
LTRRT_1444	0	0	LTRRT_1444	0	0
LTRRT_1445	2	197.7457	LTRRT_1445	0	0
LTRRT_1446	0	0	LTRRT_1446	0	0
LTRRT_1447	0	0	LTRRT_1447	1	35.447166
LTRRT_1448	0	0	LTRRT_1448	0	0

LTRRT_1449	0	0	LTRRT_1449	0	0
LTRRT_1450	0	0	LTRRT_1450	0	0
LTRRT_1451	0	0	LTRRT_1451	6	212.683
LTRRT_1452	0	0	LTRRT_1452	0	0
LTRRT_1453	0	0	LTRRT_1453	0	0
LTRRT_1454	0	0	LTRRT_1454	0	0
LTRRT_1455	0	0	LTRRT_1455	0	0
LTRRT_1456	0	0	LTRRT_1456	8	283.57733
LTRRT_1457	0	0	LTRRT_1457	0	0
LTRRT_1458	0	0	LTRRT_1458	0	0
LTRRT_1459	0	0	LTRRT_1459	18	638.04899
LTRRT_1460	0	0	LTRRT_1460	40	1417.8866
LTRRT_1461	0	0	LTRRT_1461	177	6274.1484
LTRRT_1462	0	0	LTRRT_1462	0	0
LTRRT_1463	0	0	LTRRT_1463	0	0
LTRRT_1464	1	98.87285	LTRRT_1464	0	0
LTRRT_1465	0	0	LTRRT_1465	3	106.3415
LTRRT_1466	0	0	LTRRT_1466	0	0
LTRRT_1467	1	98.87285	LTRRT_1467	3	106.3415
LTRRT_1468	15	1483.0927	LTRRT_1468	0	0
LTRRT_1469	0	0	LTRRT_1469	0	0
LTRRT_1470	2	197.7457	LTRRT_1470	0	0
LTRRT_1471	0	0	LTRRT_1471	0	0
LTRRT_1472	0	0	LTRRT_1472	0	0
LTRRT_1473	4	395.4914	LTRRT_1473	6	212.683
LTRRT_1474	0	0	LTRRT_1474	0	0
LTRRT_1475	1	98.87285	LTRRT_1475	0	0
LTRRT_1476	1	98.87285	LTRRT_1476	0	0
LTRRT_1477	0	0	LTRRT_1477	0	0
LTRRT_1478	0	0	LTRRT_1478	0	0
LTRRT_1479	0	0	LTRRT_1479	0	0
LTRRT_1480	0	0	LTRRT_1480	3	106.3415
LTRRT_1481	0	0	LTRRT_1481	2	70.894332
LTRRT_1482	0	0	LTRRT_1482	0	0
LTRRT_1483	0	0	LTRRT_1483	0	0
LTRRT_1484	0	0	LTRRT_1484	2	70.894332
LTRRT_1485	14	1384.2199	LTRRT_1485	0	0
LTRRT_1486	0	0	LTRRT_1486	0	0
LTRRT_1487	0	0	LTRRT_1487	0	0
LTRRT_1488	0	0	LTRRT_1488	0	0
LTRRT_1489	1	98.87285	LTRRT_1489	79	2800.3261
LTRRT_1490	0	0	LTRRT_1490	0	0
LTRRT_1491	0	0	LTRRT_1491	0	0
LTRRT_1492	0	0	LTRRT_1492	0	0

LTRRT_1493	0	0	LTRRT_1493	0	0
LTRRT_1494	0	0	LTRRT_1494	0	0
LTRRT_1495	0	0	LTRRT_1495	0	0
LTRRT_1496	0	0	LTRRT_1496	864	30626.351
LTRRT_1497	1	98.87285	LTRRT_1497	0	0
LTRRT_1498	0	0	LTRRT_1498	0	0
LTRRT_1499	0	0	LTRRT_1499	0	0
LTRRT_1500	0	0	LTRRT_1500	0	0
LTRRT_1501	0	0	LTRRT_1501	0	0
LTRRT_1502	0	0	LTRRT_1502	0	0
LTRRT_1503	1	98.87285	LTRRT_1503	0	0
LTRRT_1504	0	0	LTRRT_1504	8	283.57733
LTRRT_1505	0	0	LTRRT_1505	0	0
LTRRT_1506	0	0	LTRRT_1506	11	389.91883
LTRRT_1507	6	593.2371	LTRRT_1507	3	106.3415
LTRRT_1508	2	197.7457	LTRRT_1508	0	0
LTRRT_1509	0	0	LTRRT_1509	6	212.683
LTRRT_1510	0	0	LTRRT_1510	2	70.894332
LTRRT_1511	0	0	LTRRT_1511	0	0
LTRRT_1512	0	0	LTRRT_1512	0	0
LTRRT_1513	0	0	LTRRT_1513	0	0
LTRRT_1514	0	0	LTRRT_1514	0	0
LTRRT_1515	0	0	LTRRT_1515	10	354.47166
LTRRT_1516	0	0	LTRRT_1516	0	0
LTRRT_1517	0	0	LTRRT_1517	0	0
LTRRT_1518	0	0	LTRRT_1518	93	3296.5864
LTRRT_1519	0	0	LTRRT_1519	0	0
LTRRT_1520	0	0	LTRRT_1520	8	283.57733
LTRRT_1521	0	0	LTRRT_1521	0	0
LTRRT_1522	2	197.7457	LTRRT_1522	19	673.49615
LTRRT_1523	0	0	LTRRT_1523	0	0
LTRRT_1524	0	0	LTRRT_1524	13	460.81316
LTRRT_1525	0	0	LTRRT_1525	2	70.894332
LTRRT_1526	0	0	LTRRT_1526	210	7443.9049
LTRRT_1527	0	0	LTRRT_1527	0	0
LTRRT_1528	2	197.7457	LTRRT_1528	0	0
LTRRT_1529	0	0	LTRRT_1529	1	35.447166
LTRRT_1530	0	0	LTRRT_1530	0	0
LTRRT_1531	0	0	LTRRT_1531	0	0
LTRRT_1532	0	0	LTRRT_1532	0	0
LTRRT_1533	0	0	LTRRT_1533	18	638.04899
LTRRT_1534	0	0	LTRRT_1534	1	35.447166
LTRRT_1535	1	98.87285	LTRRT_1535	0	0
LTRRT_1536	7	692.10995	LTRRT_1536	0	0

LTRRT_1537	0	0	LTRRT_1537	0	0
LTRRT_1538	1	98.87285	LTRRT_1538	1	35.447166
LTRRT_1539	0	0	LTRRT_1539	0	0
LTRRT_1540	1	98.87285	LTRRT_1540	1	35.447166
LTRRT_1541	0	0	LTRRT_1541	0	0
LTRRT_1542	8	790.9828	LTRRT_1542	0	0
LTRRT_1543	0	0	LTRRT_1543	0	0
LTRRT_1544	0	0	LTRRT_1544	0	0
LTRRT_1545	9	889.85565	LTRRT_1545	4	141.78866
LTRRT_1546	0	0	LTRRT_1546	0	0
LTRRT_1547	0	0	LTRRT_1547	0	0
LTRRT_1548	0	0	LTRRT_1548	0	0
LTRRT_1549	0	0	LTRRT_1549	0	0
LTRRT_1550	0	0	LTRRT_1550	13	460.81316
LTRRT_1551	0	0	LTRRT_1551	1	35.447166
LTRRT_1552	12	1186.4742	LTRRT_1552	0	0
LTRRT_1553	0	0	LTRRT_1553	0	0
LTRRT_1554	0	0	LTRRT_1554	0	0
LTRRT_1555	0	0	LTRRT_1555	5	177.23583
LTRRT_1556	0	0	LTRRT_1556	9	319.02449
LTRRT_1557	0	0	LTRRT_1557	0	0
LTRRT_1558	0	0	LTRRT_1558	0	0
LTRRT_1559	0	0	LTRRT_1559	0	0
LTRRT_1560	0	0	LTRRT_1560	0	0
LTRRT_1561	1	98.87285	LTRRT_1561	0	0
LTRRT_1562	9	889.85565	LTRRT_1562	0	0
LTRRT_1563	0	0	LTRRT_1563	0	0
LTRRT_1564	0	0	LTRRT_1564	0	0
LTRRT_1565	0	0	LTRRT_1565	0	0
LTRRT_1566	0	0	LTRRT_1566	0	0
LTRRT_1567	0	0	LTRRT_1567	0	0
LTRRT_1568	0	0	LTRRT_1568	6	212.683
LTRRT_1569	0	0	LTRRT_1569	15	531.70749
LTRRT_1570	0	0	LTRRT_1570	0	0
LTRRT_1571	0	0	LTRRT_1571	0	0
LTRRT_1572	0	0	LTRRT_1572	7	248.13016
LTRRT_1573	0	0	LTRRT_1573	0	0
LTRRT_1574	0	0	LTRRT_1574	0	0
LTRRT_1575	0	0	LTRRT_1575	7	248.13016
LTRRT_1576	0	0	LTRRT_1576	0	0
LTRRT_1577	0	0	LTRRT_1577	0	0
LTRRT_1578	1	98.87285	LTRRT_1578	0	0
LTRRT_1579	1	98.87285	LTRRT_1579	1	35.447166
LTRRT_1580	0	0	LTRRT_1580	0	0

LTRRT_1581	0	0	LTRRT_1581	0	0
LTRRT_1582	1	98.87285	LTRRT_1582	0	0
LTRRT_1583	0	0	LTRRT_1583	0	0
LTRRT_1584	0	0	LTRRT_1584	0	0
LTRRT_1585	0	0	LTRRT_1585	0	0
LTRRT_1586	0	0	LTRRT_1586	10	354.47166
LTRRT_1587	0	0	LTRRT_1587	0	0
LTRRT_1588	0	0	LTRRT_1588	0	0
LTRRT_1589	0	0	LTRRT_1589	0	0
LTRRT_1590	0	0	LTRRT_1590	86	3048.4563
LTRRT_1591	0	0	LTRRT_1591	16	567.15466
LTRRT_1592	0	0	LTRRT_1592	0	0
LTRRT_1593	0	0	LTRRT_1593	0	0
LTRRT_1594	0	0	LTRRT_1594	0	0
LTRRT_1595	0	0	LTRRT_1595	0	0
LTRRT_1596	0	0	LTRRT_1596	5	177.23583
LTRRT_1597	0	0	LTRRT_1597	8	283.57733
LTRRT_1598	0	0	LTRRT_1598	10	354.47166
LTRRT_1599	0	0	LTRRT_1599	12	425.36599
LTRRT_1600	0	0	LTRRT_1600	0	0
LTRRT_1601	0	0	LTRRT_1601	0	0
LTRRT_1602	0	0	LTRRT_1602	0	0
LTRRT_1603	0	0	LTRRT_1603	0	0
LTRRT_1604	0	0	LTRRT_1604	14	496.26032
LTRRT_1605	0	0	LTRRT_1605	0	0
LTRRT_1606	0	0	LTRRT_1606	0	0
LTRRT_1607	1	98.87285	LTRRT_1607	0	0
LTRRT_1608	1	98.87285	LTRRT_1608	0	0
LTRRT_1609	1	98.87285	LTRRT_1609	0	0
LTRRT_1610	30	2966.1855	LTRRT_1610	0	0
LTRRT_1611	0	0	LTRRT_1611	0	0
LTRRT_1612	0	0	LTRRT_1612	0	0
LTRRT_1613	0	0	LTRRT_1613	0	0
LTRRT_1614	0	0	LTRRT_1614	222	7869.2709
LTRRT_1615	28	2768.4398	LTRRT_1615	88	3119.3506
LTRRT_1616	0	0	LTRRT_1616	0	0
LTRRT_1617	0	0	LTRRT_1617	0	0
LTRRT_1618	0	0	LTRRT_1618	0	0
LTRRT_1619	3	296.61855	LTRRT_1619	13	460.81316
LTRRT_1620	1	98.87285	LTRRT_1620	1	35.447166
LTRRT_1621	0	0	LTRRT_1621	393	13930.736
LTRRT_1622	0	0	LTRRT_1622	0	0
LTRRT_1623	0	0	LTRRT_1623	0	0
LTRRT_1624	0	0	LTRRT_1624	0	0

LTRRT_1625	1	98.87285
LTRRT_1626	0	0
LTRRT_1627	1	98.87285
LTRRT_1628	0	0
LTRRT_1629	0	0
LTRRT_1630	3	296.61855
LTRRT_1631	0	0
LTRRT_1632	0	0
LTRRT_1633	0	0
LTRRT_1634	0	0
LTRRT_1635	3	296.61855
LTRRT_1636	5	494.36425
LTRRT_1637	0	0
LTRRT_1638	0	0
LTRRT_1639	0	0
LTRRT_1640	2	197.7457
LTRRT_1641	0	0
LTRRT_1642	0	0
LTRRT_1643	0	0
LTRRT_1644	0	0
LTRRT_1645	0	0
LTRRT_1646	7	692.10995
LTRRT_1647	0	0
LTRRT_1648	0	0
LTRRT_1649	0	0
LTRRT_1650	6	593.2371
LTRRT_1651	0	0
LTRRT_1652	0	0
LTRRT_1653	0	0
LTRRT_1654	0	0
LTRRT_1655	1	98.87285
LTRRT_1656	19	1878.5841
LTRRT_1657	36	3559.4226
LTRRT_1658	0	0
LTRRT_1659	0	0
LTRRT_1660	0	0
LTRRT_1661	0	0
LTRRT_1662	0	0
LTRRT_1663	0	0
LTRRT_1664	23	2274.0755
LTRRT_1665	0	0
LTRRT_1666	0	0
LTRRT_1667	1	98.87285
LTRRT_1668	0	0

LTRRT_1625	171	6061.4654
LTRRT_1626	0	0
LTRRT_1627	0	0
LTRRT_1628	1	35.447166
LTRRT_1629	0	0
LTRRT_1630	10	354.47166
LTRRT_1631	0	0
LTRRT_1632	4	141.78866
LTRRT_1633	37	1311.5451
LTRRT_1634	0	0
LTRRT_1635	0	0

LTRRT_1669	10	988.7285
LTRRT_1670	0	0
LTRRT_1671	1	98.87285
LTRRT_1672	0	0
LTRRT_1673	0	0
LTRRT_1674	0	0
LTRRT_1675	0	0
LTRRT_1676	0	0
LTRRT_1677	0	0
LTRRT_1678	0	0
LTRRT_1679	0	0
LTRRT_1680	0	0
LTRRT_1681	1	98.87285
LTRRT_1682	0	0
LTRRT_1683	1	98.87285
LTRRT_1684	49	4844.7696
LTRRT_1685	0	0
LTRRT_1686	0	0
LTRRT_1687	0	0
LTRRT_1688	0	0
LTRRT_1689	0	0
LTRRT_1690	0	0
LTRRT_1691	0	0
LTRRT_1692	0	0
LTRRT_1693	0	0
LTRRT_1694	0	0
LTRRT_1695	0	0
LTRRT_1696	0	0
LTRRT_1697	0	0
LTRRT_1698	2	197.7457
LTRRT_1699	0	0
LTRRT_1700	0	0
LTRRT_1701	0	0
LTRRT_1702	0	0
LTRRT_1703	1	98.87285
LTRRT_1704	0	0
LTRRT_1705	0	0
LTRRT_1706	0	0
LTRRT_1707	1	98.87285
LTRRT_1708	2	197.7457
LTRRT_1709	0	0
LTRRT_1710	0	0
LTRRT_1711	0	0
LTRRT_1712	0	0

LTRRT_1713	0	0
LTRRT_1714	0	0
LTRRT_1715	0	0
LTRRT_1716	1	98.87285
LTRRT_1717	0	0
LTRRT_1718	0	0
LTRRT_1719	0	0
LTRRT_1720	1	98.87285
LTRRT_1721	0	0
LTRRT_1722	0	0
LTRRT_1723	0	0
LTRRT_1724	0	0
LTRRT_1725	0	0
LTRRT_1726	0	0
LTRRT_1727	0	0
LTRRT_1728	0	0
LTRRT_1729	3	296.61855
LTRRT_1730	0	0
LTRRT_1731	0	0
LTRRT_1732	0	0
LTRRT_1733	0	0
LTRRT_1734	0	0
LTRRT_1735	0	0
LTRRT_1736	0	0
LTRRT_1737	4	395.4914
LTRRT_1738	0	0
LTRRT_1739	0	0
LTRRT_1740	0	0
LTRRT_1741	0	0
LTRRT_1742	0	0
LTRRT_1743	3	296.61855
LTRRT_1744	0	0
LTRRT_1745	0	0
LTRRT_1746	5	494.36425
LTRRT_1747	0	0
LTRRT_1748	0	0
LTRRT_1749	0	0
LTRRT_1750	0	0
LTRRT_1751	0	0
LTRRT_1752	33	3262.804
LTRRT_1753	0	0
LTRRT_1754	0	0
LTRRT_1755	1	98.87285
LTRRT_1756	0	0

LTRRT_1757	0	0
LTRRT_1758	0	0
LTRRT_1759	0	0
LTRRT_1760	0	0
LTRRT_1761	0	0
LTRRT_1762	0	0
LTRRT_1763	0	0
LTRRT_1764	0	0
LTRRT_1765	0	0
LTRRT_1766	0	0
LTRRT_1767	0	0
LTRRT_1768	0	0
LTRRT_1769	1	98.87285
LTRRT_1770	2	197.7457
LTRRT_1771	0	0
LTRRT_1772	0	0
LTRRT_1773	0	0
LTRRT_1774	6	593.2371
LTRRT_1775	0	0
LTRRT_1776	0	0
LTRRT_1777	1	98.87285
LTRRT_1778	551	54478.94
LTRRT_1779	0	0
LTRRT_1780	0	0
LTRRT_1781	0	0
LTRRT_1782	0	0
LTRRT_1783	0	0
LTRRT_1784	0	0
LTRRT_1785	1	98.87285
LTRRT_1786	0	0
LTRRT_1787	22	2175.2027
LTRRT_1788	15	1483.0927
LTRRT_1789	24	2372.9484
LTRRT_1790	0	0
LTRRT_1791	1	98.87285
LTRRT_1792	0	0
LTRRT_1793	23	2274.0755
LTRRT_1794	0	0
LTRRT_1795	0	0
LTRRT_1796	0	0
LTRRT_1797	2	197.7457
LTRRT_1798	2	197.7457
LTRRT_1799	1	98.87285
LTRRT_1800	0	0

LTRRT_1801	0	0
LTRRT_1802	0	0
LTRRT_1803	0	0
LTRRT_1804	0	0
LTRRT_1805	11	1087.6013
LTRRT_1806	0	0
LTRRT_1807	0	0
LTRRT_1808	0	0
LTRRT_1809	0	0
LTRRT_1810	0	0
LTRRT_1811	0	0
LTRRT_1812	0	0
LTRRT_1813	0	0
LTRRT_1814	51	5042.5153
LTRRT_1815	0	0
LTRRT_1816	0	0
LTRRT_1817	0	0
LTRRT_1818	0	0
LTRRT_1819	0	0
LTRRT_1820	0	0
LTRRT_1821	0	0
LTRRT_1822	1	98.87285
LTRRT_1823	0	0
LTRRT_1824	13	1285.347
LTRRT_1825	2	197.7457
LTRRT_1826	0	0
LTRRT_1827	0	0
LTRRT_1828	0	0
LTRRT_1829	8	790.9828
LTRRT_1830	14	1384.2199
LTRRT_1831	0	0
LTRRT_1832	0	0
LTRRT_1833	0	0
LTRRT_1834	1	98.87285
LTRRT_1835	0	0
LTRRT_1836	0	0
LTRRT_1837	0	0
LTRRT_1838	1	98.87285
LTRRT_1839	0	0
LTRRT_1840	11	1087.6013
LTRRT_1841	0	0
LTRRT_1842	0	0
LTRRT_1843	0	0
LTRRT_1844	0	0

LTRRT_1845	0	0
LTRRT_1846	1	98.87285
LTRRT_1847	4	395.4914
LTRRT_1848	1	98.87285
LTRRT_1849	11	1087.6013
LTRRT_1850	0	0
LTRRT_1851	0	0
LTRRT_1852	0	0
LTRRT_1853	0	0
LTRRT_1854	0	0
LTRRT_1855	0	0
LTRRT_1856	0	0
LTRRT_1857	0	0
LTRRT_1858	0	0
LTRRT_1859	0	0
LTRRT_1860	0	0
LTRRT_1861	107	10579.395
LTRRT_1862	0	0
LTRRT_1863	3	296.61855
LTRRT_1864	0	0
LTRRT_1865	0	0
LTRRT_1866	17	1680.8384
LTRRT_1867	0	0
LTRRT_1868	3	296.61855
LTRRT_1869	0	0
LTRRT_1870	0	0
LTRRT_1871	11	1087.6013
LTRRT_1872	0	0
LTRRT_1873	0	0
LTRRT_1874	0	0
LTRRT_1875	0	0
LTRRT_1876	0	0
LTRRT_1877	0	0
LTRRT_1878	0	0
LTRRT_1879	0	0
LTRRT_1880	0	0
LTRRT_1881	0	0
LTRRT_1882	0	0
LTRRT_1883	0	0
LTRRT_1884	0	0
LTRRT_1885	1	98.87285
LTRRT_1886	0	0
LTRRT_1887	12	1186.4742
LTRRT_1888	0	0

LTRRT_1889	0	0
LTRRT_1890	0	0
LTRRT_1891	0	0
LTRRT_1892	0	0
LTRRT_1893	0	0
LTRRT_1894	16	1581.9656
LTRRT_1895	7	692.10995
LTRRT_1896	2	197.7457
LTRRT_1897	1	98.87285
LTRRT_1898	0	0
LTRRT_1899	0	0
LTRRT_1900	0	0
LTRRT_1901	1	98.87285
LTRRT_1902	0	0
LTRRT_1903	2	197.7457
LTRRT_1904	16	1581.9656
LTRRT_1905	0	0
LTRRT_1906	3	296.61855
LTRRT_1907	0	0
LTRRT_1908	4	395.4914
LTRRT_1909	0	0
LTRRT_1910	0	0
LTRRT_1911	0	0
LTRRT_1912	3	296.61855
LTRRT_1913	0	0
LTRRT_1914	0	0
LTRRT_1915	0	0
LTRRT_1916	4	395.4914
LTRRT_1917	16	1581.9656
LTRRT_1918	0	0
LTRRT_1919	0	0
LTRRT_1920	0	0
LTRRT_1921	0	0
LTRRT_1922	1	98.87285
LTRRT_1923	13	1285.347
LTRRT_1924	2	197.7457
LTRRT_1925	0	0
LTRRT_1926	4	395.4914
LTRRT_1927	0	0
LTRRT_1928	0	0
LTRRT_1929	3	296.61855
LTRRT_1930	0	0
LTRRT_1931	0	0
LTRRT_1932	0	0

LTRRT_1933	0	0
LTRRT_1934	2	197.7457
LTRRT_1935	0	0
LTRRT_1936	14	1384.2199
LTRRT_1937	0	0
LTRRT_1938	1	98.87285
LTRRT_1939	2	197.7457
LTRRT_1940	0	0
LTRRT_1941	0	0
LTRRT_1942	8	790.9828
LTRRT_1943	0	0
LTRRT_1944	0	0
LTRRT_1945	0	0
LTRRT_1946	0	0
LTRRT_1947	0	0
LTRRT_1948	23	2274.0755
LTRRT_1949	0	0
LTRRT_1950	0	0
LTRRT_1951	0	0
LTRRT_1952	0	0
LTRRT_1953	0	0
LTRRT_1954	0	0
LTRRT_1955	0	0
LTRRT_1956	0	0
LTRRT_1957	0	0
LTRRT_1958	0	0
LTRRT_1959	3	296.61855
LTRRT_1960	1	98.87285
LTRRT_1961	0	0
LTRRT_1962	0	0
LTRRT_1963	2	197.7457
LTRRT_1964	0	0
LTRRT_1965	0	0
LTRRT_1966	0	0
LTRRT_1967	0	0
LTRRT_1968	0	0
LTRRT_1969	0	0
LTRRT_1970	0	0
LTRRT_1971	1	98.87285
LTRRT_1972	1	98.87285
LTRRT_1973	0	0
LTRRT_1974	1	98.87285
LTRRT_1975	0	0
LTRRT_1976	36	3559.4226

LTRRT_1977	0	0
LTRRT_1978	1	98.87285
LTRRT_1979	0	0
LTRRT_1980	0	0
LTRRT_1981	0	0
LTRRT_1982	0	0
LTRRT_1983	0	0
LTRRT_1984	2	197.7457
LTRRT_1985	13	1285.347
LTRRT_1986	0	0
LTRRT_1987	1	98.87285
LTRRT_1988	9	889.85565
LTRRT_1989	0	0
LTRRT_1990	0	0
LTRRT_1991	0	0
LTRRT_1992	0	0
LTRRT_1993	0	0
LTRRT_1994	0	0
LTRRT_1995	0	0
LTRRT_1996	0	0
LTRRT_1997	0	0
LTRRT_1998	0	0
LTRRT_1999	28	2768.4398
LTRRT_2000	0	0
LTRRT_2001	1	98.87285
LTRRT_2002	1	98.87285
LTRRT_2003	3	296.61855
LTRRT_2004	1	98.87285
LTRRT_2005	0	0
LTRRT_2006	0	0
LTRRT_2007	0	0
LTRRT_2008	1	98.87285
LTRRT_2009	10	988.7285
LTRRT_2010	0	0
LTRRT_2011	1	98.87285
LTRRT_2012	3	296.61855
LTRRT_2013	0	0
LTRRT_2014	0	0
LTRRT_2015	0	0
LTRRT_2016	0	0
LTRRT_2017	0	0
LTRRT_2018	5	494.36425
LTRRT_2019	0	0
LTRRT_2020	8	790.9828

LTRRT_2021	0	0
LTRRT_2022	1	98.87285
LTRRT_2023	3	296.61855
LTRRT_2024	1	98.87285
LTRRT_2025	2	197.7457
LTRRT_2026	2	197.7457
LTRRT_2027	7	692.10995
LTRRT_2028	0	0
LTRRT_2029	0	0
LTRRT_2030	0	0
LTRRT_2031	0	0
LTRRT_2032	2	197.7457
LTRRT_2033	47	4647.0239
LTRRT_2034	0	0
LTRRT_2035	0	0
LTRRT_2036	0	0
LTRRT_2037	0	0
LTRRT_2038	0	0
LTRRT_2039	0	0
LTRRT_2040	0	0
LTRRT_2041	0	0
LTRRT_2042	0	0
LTRRT_2043	3	296.61855
LTRRT_2044	0	0
LTRRT_2045	0	0
LTRRT_2046	7	692.10995
LTRRT_2047	0	0
LTRRT_2048	0	0
LTRRT_2049	0	0
LTRRT_2050	1	98.87285
LTRRT_2051	0	0
LTRRT_2052	0	0
LTRRT_2053	0	0
LTRRT_2054	0	0
LTRRT_2055	0	0
LTRRT_2056	0	0
LTRRT_2057	0	0
LTRRT_2058	0	0
LTRRT_2059	1	98.87285
LTRRT_2060	30	2966.1855
LTRRT_2061	0	0
LTRRT_2062	0	0
LTRRT_2063	12	1186.4742
LTRRT_2064	0	0

LTRRT_2065	10	988.7285
LTRRT_2066	2	197.7457
LTRRT_2067	0	0
LTRRT_2068	0	0
LTRRT_2069	1	98.87285
LTRRT_2070	7	692.10995
LTRRT_2071	2	197.7457
LTRRT_2072	0	0
LTRRT_2073	0	0
LTRRT_2074	1	98.87285
LTRRT_2075	15	1483.0927
LTRRT_2076	0	0
LTRRT_2077	0	0
LTRRT_2078	1	98.87285
LTRRT_2079	0	0
LTRRT_2080	0	0
LTRRT_2081	3	296.61855
LTRRT_2082	2	197.7457
LTRRT_2083	0	0
LTRRT_2084	0	0
LTRRT_2085	0	0
LTRRT_2086	1	98.87285
LTRRT_2087	0	0
LTRRT_2088	0	0
LTRRT_2089	0	0
LTRRT_2090	0	0
LTRRT_2091	1	98.87285
LTRRT_2092	1	98.87285
LTRRT_2093	10	988.7285
LTRRT_2094	0	0
LTRRT_2095	2	197.7457
LTRRT_2096	0	0
LTRRT_2097	0	0
LTRRT_2098	0	0
LTRRT_2099	0	0
LTRRT_2100	8	790.9828
LTRRT_2101	0	0
LTRRT_2102	0	0
LTRRT_2103	177	17500.494
LTRRT_2104	0	0
LTRRT_2105	0	0
LTRRT_2106	0	0
LTRRT_2107	0	0
LTRRT_2108	1	98.87285

LTRRT_2109	8	790.9828
LTRRT_2110	0	0
LTRRT_2111	0	0
LTRRT_2112	0	0
LTRRT_2113	1	98.87285
LTRRT_2114	0	0
LTRRT_2115	0	0
LTRRT_2116	0	0
LTRRT_2117	0	0
LTRRT_2118	0	0
LTRRT_2119	0	0
LTRRT_2120	0	0
LTRRT_2121	0	0
LTRRT_2122	0	0
LTRRT_2123	0	0
LTRRT_2124	1	98.87285
LTRRT_2125	0	0
LTRRT_2126	0	0
LTRRT_2127	0	0
LTRRT_2128	0	0
LTRRT_2129	0	0
LTRRT_2130	0	0
LTRRT_2131	2	197.7457
LTRRT_2132	0	0
LTRRT_2133	0	0
LTRRT_2134	2	197.7457
LTRRT_2135	0	0
LTRRT_2136	0	0
LTRRT_2137	0	0
LTRRT_2138	0	0
LTRRT_2139	33	3262.804
LTRRT_2140	3	296.61855
LTRRT_2141	0	0
LTRRT_2142	0	0
LTRRT_2143	0	0
LTRRT_2144	0	0
LTRRT_2145	0	0
LTRRT_2146	0	0
LTRRT_2147	0	0
LTRRT_2148	0	0
LTRRT_2149	0	0
LTRRT_2150	5	494.36425
LTRRT_2151	0	0
LTRRT_2152	0	0

LTRRT_2153	0	0
LTRRT_2154	0	0
LTRRT_2155	0	0
LTRRT_2156	0	0
LTRRT_2157	0	0
LTRRT_2158	0	0
LTRRT_2159	0	0
LTRRT_2160	0	0
LTRRT_2161	0	0
LTRRT_2162	0	0
LTRRT_2163	0	0
LTRRT_2164	1	98.87285
LTRRT_2165	6	593.2371
LTRRT_2166	0	0
LTRRT_2167	0	0
LTRRT_2168	0	0
LTRRT_2169	0	0
LTRRT_2170	0	0
LTRRT_2171	0	0
LTRRT_2172	5	494.36425
LTRRT_2173	24	2372.9484
LTRRT_2174	0	0
LTRRT_2175	0	0
LTRRT_2176	0	0
LTRRT_2177	0	0
LTRRT_2178	0	0
LTRRT_2179	0	0
LTRRT_2180	0	0
LTRRT_2181	0	0
LTRRT_2182	0	0
LTRRT_2183	0	0
LTRRT_2184	2	197.7457
LTRRT_2185	0	0
LTRRT_2186	0	0
LTRRT_2187	0	0
LTRRT_2188	0	0
LTRRT_2189	0	0
LTRRT_2190	0	0
LTRRT_2191	0	0
LTRRT_2192	0	0
LTRRT_2193	0	0
LTRRT_2194	1	98.87285
LTRRT_2195	1	98.87285
LTRRT_2196	0	0

LTRRT_2197	0	0
LTRRT_2198	0	0
LTRRT_2199	0	0
LTRRT_2200	1	98.87285
LTRRT_2201	1	98.87285
LTRRT_2202	1	98.87285
LTRRT_2203	0	0
LTRRT_2204	0	0
LTRRT_2205	0	0
LTRRT_2206	0	0
LTRRT_2207	0	0
LTRRT_2208	0	0
LTRRT_2209	0	0
LTRRT_2210	0	0
LTRRT_2211	1	98.87285
LTRRT_2212	2	197.7457
LTRRT_2213	0	0
LTRRT_2214	0	0
LTRRT_2215	1	98.87285
LTRRT_2216	0	0
LTRRT_2217	0	0
LTRRT_2218	1	98.87285
LTRRT_2219	0	0
LTRRT_2220	2	197.7457
LTRRT_2221	0	0
LTRRT_2222	1	98.87285
LTRRT_2223	1	98.87285
LTRRT_2224	929	91852.877
LTRRT_2225	0	0
LTRRT_2226	0	0
LTRRT_2227	0	0
LTRRT_2228	0	0
LTRRT_2229	0	0
LTRRT_2230	14	1384.2199
LTRRT_2231	0	0
LTRRT_2232	0	0
LTRRT_2233	0	0
LTRRT_2234	0	0
LTRRT_2235	0	0
LTRRT_2236	0	0
LTRRT_2237	0	0
LTRRT_2238	0	0
LTRRT_2239	4	395.4914
LTRRT_2240	0	0

LTRRT_2241	0	0
LTRRT_2242	0	0
LTRRT_2243	0	0
LTRRT_2244	11	1087.6013
LTRRT_2245	1	98.87285
LTRRT_2246	0	0
LTRRT_2247	0	0
LTRRT_2248	0	0
LTRRT_2249	0	0
LTRRT_2250	1	98.87285
LTRRT_2251	4	395.4914
LTRRT_2252	20	1977.457
LTRRT_2253	1	98.87285
LTRRT_2254	2	197.7457
LTRRT_2255	3	296.61855
LTRRT_2256	2	197.7457
LTRRT_2257	1	98.87285
LTRRT_2258	0	0
LTRRT_2259	0	0
LTRRT_2260	0	0
LTRRT_2261	0	0
LTRRT_2262	1	98.87285
LTRRT_2263	14	1384.2199
LTRRT_2264	1	98.87285
LTRRT_2265	0	0
LTRRT_2266	0	0
LTRRT_2267	12	1186.4742
LTRRT_2268	5	494.36425
LTRRT_2269	0	0
LTRRT_2270	2	197.7457
LTRRT_2271	0	0
LTRRT_2272	0	0
LTRRT_2273	0	0
LTRRT_2274	0	0
LTRRT_2275	0	0
LTRRT_2276	0	0
LTRRT_2277	0	0
LTRRT_2278	0	0
LTRRT_2279	1	98.87285
LTRRT_2280	0	0
LTRRT_2281	5	494.36425
LTRRT_2282	0	0
LTRRT_2283	0	0
LTRRT_2284	0	0

LTRRT_2285	0	0
LTRRT_2286	0	0
LTRRT_2287	0	0
LTRRT_2288	0	0
LTRRT_2289	1	98.87285
LTRRT_2290	0	0
LTRRT_2291	91	8997.4293
LTRRT_2292	1	98.87285
LTRRT_2293	0	0
LTRRT_2294	0	0
LTRRT_2295	0	0
LTRRT_2296	0	0
LTRRT_2297	0	0
LTRRT_2298	0	0
LTRRT_2299	0	0
LTRRT_2300	0	0
LTRRT_2301	0	0
LTRRT_2302	0	0
LTRRT_2303	2	197.7457
LTRRT_2304	0	0
LTRRT_2305	0	0
LTRRT_2306	0	0
LTRRT_2307	0	0
LTRRT_2308	0	0
LTRRT_2309	0	0
LTRRT_2310	0	0
LTRRT_2311	0	0
LTRRT_2312	2	197.7457
LTRRT_2313	25	2471.8212
LTRRT_2314	1	98.87285
LTRRT_2315	0	0
LTRRT_2316	50	4943.6425
LTRRT_2317	0	0
LTRRT_2318	0	0
LTRRT_2319	0	0
LTRRT_2320	3	296.61855
LTRRT_2321	0	0
LTRRT_2322	4	395.4914
LTRRT_2323	0	0
LTRRT_2324	0	0
LTRRT_2325	0	0
LTRRT_2326	14	1384.2199
LTRRT_2327	0	0
LTRRT_2328	0	0

LTRRT_2329	0	0
LTRRT_2330	0	0
LTRRT_2331	0	0
LTRRT_2332	0	0
LTRRT_2333	0	0
LTRRT_2334	0	0
LTRRT_2335	0	0
LTRRT_2336	1	98.87285
LTRRT_2337	0	0
LTRRT_2338	0	0
LTRRT_2339	0	0
LTRRT_2340	1	98.87285
LTRRT_2341	0	0
LTRRT_2342	0	0
LTRRT_2343	1	98.87285
LTRRT_2344	0	0
LTRRT_2345	0	0
LTRRT_2346	0	0
LTRRT_2347	0	0
LTRRT_2348	0	0
LTRRT_2349	10	988.7285
LTRRT_2350	0	0
LTRRT_2351	0	0
LTRRT_2352	1	98.87285
LTRRT_2353	0	0
LTRRT_2354	0	0
LTRRT_2355	0	0
LTRRT_2356	0	0
LTRRT_2357	1	98.87285
LTRRT_2358	0	0
LTRRT_2359	0	0
LTRRT_2360	2	197.7457
LTRRT_2361	0	0
LTRRT_2362	10	988.7285
LTRRT_2363	0	0
LTRRT_2364	0	0
LTRRT_2365	0	0
LTRRT_2366	0	0
LTRRT_2367	0	0
LTRRT_2368	0	0
LTRRT_2369	0	0
LTRRT_2370	0	0
LTRRT_2371	0	0
LTRRT_2372	0	0

LTRRT_2373	1	98.87285
LTRRT_2374	0	0
LTRRT_2375	0	0
LTRRT_2376	0	0
LTRRT_2377	0	0
LTRRT_2378	2	197.7457
LTRRT_2379	0	0
LTRRT_2380	0	0
LTRRT_2381	0	0
LTRRT_2382	3	296.61855
LTRRT_2383	0	0
LTRRT_2384	0	0
LTRRT_2385	0	0
LTRRT_2386	1	98.87285
LTRRT_2387	1	98.87285
LTRRT_2388	1	98.87285
LTRRT_2389	0	0
LTRRT_2390	0	0
LTRRT_2391	0	0
LTRRT_2392	0	0
LTRRT_2393	0	0
LTRRT_2394	0	0
LTRRT_2395	0	0
LTRRT_2396	0	0
LTRRT_2397	5	494.36425
LTRRT_2398	0	0
LTRRT_2399	0	0
LTRRT_2400	0	0
LTRRT_2401	26	2570.6941
LTRRT_2402	0	0
LTRRT_2403	0	0
LTRRT_2404	0	0
LTRRT_2405	0	0
LTRRT_2406	0	0
LTRRT_2407	0	0
LTRRT_2408	0	0
LTRRT_2409	0	0
LTRRT_2410	74	7316.5909
LTRRT_2411	0	0
LTRRT_2412	0	0
LTRRT_2413	0	0
LTRRT_2414	0	0
LTRRT_2415	0	0
LTRRT_2416	2	197.7457

LTRRT_2417	6	593.2371
LTRRT_2418	2	197.7457
LTRRT_2419	0	0
LTRRT_2420	0	0
LTRRT_2421	0	0
LTRRT_2422	0	0
LTRRT_2423	0	0
LTRRT_2424	7	692.10995
LTRRT_2425	0	0
LTRRT_2426	13	1285.347
LTRRT_2427	0	0
LTRRT_2428	2	197.7457
LTRRT_2429	5	494.36425
LTRRT_2430	0	0
LTRRT_2431	0	0
LTRRT_2432	1	98.87285
LTRRT_2433	1	98.87285
LTRRT_2434	1	98.87285
LTRRT_2435	1	98.87285
LTRRT_2436	0	0
LTRRT_2437	0	0
LTRRT_2438	3	296.61855
LTRRT_2439	0	0
LTRRT_2440	2	197.7457
LTRRT_2441	0	0
LTRRT_2442	0	0
LTRRT_2443	0	0
LTRRT_2444	0	0
LTRRT_2445	0	0
LTRRT_2446	1	98.87285
LTRRT_2447	0	0
LTRRT_2448	0	0
LTRRT_2449	0	0
LTRRT_2450	0	0
LTRRT_2451	2	197.7457
LTRRT_2452	0	0
LTRRT_2453	0	0
LTRRT_2454	0	0
LTRRT_2455	0	0
LTRRT_2456	8	790.9828
LTRRT_2457	0	0
LTRRT_2458	0	0
LTRRT_2459	0	0
LTRRT_2460	0	0

LTRRT_2461	0	0
LTRRT_2462	200	19774.57
LTRRT_2463	2	197.7457
LTRRT_2464	0	0
LTRRT_2465	0	0
LTRRT_2466	0	0
LTRRT_2467	1	98.87285
LTRRT_2468	0	0
LTRRT_2469	0	0
LTRRT_2470	0	0
LTRRT_2471	10	988.7285
LTRRT_2472	14	1384.2199
LTRRT_2473	0	0
LTRRT_2474	0	0
LTRRT_2475	0	0
LTRRT_2476	0	0
LTRRT_2477	0	0
LTRRT_2478	0	0
LTRRT_2479	0	0
LTRRT_2480	0	0
LTRRT_2481	0	0
LTRRT_2482	0	0
LTRRT_2483	0	0
LTRRT_2484	8	790.9828
LTRRT_2485	0	0
LTRRT_2486	1	98.87285
LTRRT_2487	0	0
LTRRT_2488	0	0
LTRRT_2489	0	0
LTRRT_2490	0	0
LTRRT_2491	5	494.36425
LTRRT_2492	1	98.87285
LTRRT_2493	0	0
LTRRT_2494	0	0
LTRRT_2495	2	197.7457
LTRRT_2496	0	0
LTRRT_2497	0	0
LTRRT_2498	0	0
LTRRT_2499	3	296.61855
LTRRT_2500	0	0
LTRRT_2501	0	0
LTRRT_2502	0	0
LTRRT_2503	1	98.87285
LTRRT_2504	0	0

LTRRT_2505	0	0
LTRRT_2506	0	0
LTRRT_2507	0	0
LTRRT_2508	5	494.36425
LTRRT_2509	0	0
LTRRT_2510	0	0
LTRRT_2511	0	0
LTRRT_2512	0	0
LTRRT_2513	0	0
LTRRT_2514	1	98.87285
LTRRT_2515	0	0
LTRRT_2516	8	790.9828
LTRRT_2517	1	98.87285
LTRRT_2518	0	0
LTRRT_2519	0	0
LTRRT_2520	0	0
LTRRT_2521	0	0
LTRRT_2522	0	0
LTRRT_2523	0	0
LTRRT_2524	2	197.7457
LTRRT_2525	0	0
LTRRT_2526	0	0
LTRRT_2527	0	0
LTRRT_2528	0	0
LTRRT_2529	27	2669.5669
LTRRT_2530	0	0
LTRRT_2531	0	0
LTRRT_2532	29	2867.3126
LTRRT_2533	0	0
LTRRT_2534	0	0
LTRRT_2535	0	0
LTRRT_2536	0	0
LTRRT_2537	0	0
LTRRT_2538	0	0
LTRRT_2539	0	0
LTRRT_2540	2	197.7457
LTRRT_2541	0	0
LTRRT_2542	0	0
LTRRT_2543	2	197.7457
LTRRT_2544	0	0
LTRRT_2545	2	197.7457
LTRRT_2546	6	593.2371
LTRRT_2547	0	0
LTRRT_2548	0	0

LTRRT_2549	0	0
LTRRT_2550	3	296.61855
LTRRT_2551	46	4548.1511
LTRRT_2552	0	0
LTRRT_2553	0	0
LTRRT_2554	3	296.61855
LTRRT_2555	0	0
LTRRT_2556	0	0
LTRRT_2557	0	0
LTRRT_2558	0	0
LTRRT_2559	0	0
LTRRT_2560	0	0
LTRRT_2561	0	0
LTRRT_2562	2	197.7457
LTRRT_2563	0	0
LTRRT_2564	176	17401.622
LTRRT_2565	1	98.87285
LTRRT_2566	0	0
LTRRT_2567	0	0
LTRRT_2568	0	0
LTRRT_2569	0	0
LTRRT_2570	0	0
LTRRT_2571	0	0
LTRRT_2572	7	692.10995
LTRRT_2573	0	0
LTRRT_2574	0	0
LTRRT_2575	10	988.7285
LTRRT_2576	0	0
LTRRT_2577	0	0
LTRRT_2578	0	0
LTRRT_2579	0	0
LTRRT_2580	0	0

Supplementary Table 5. Criteria for assessment of genomic quality.

Species	BUSCO (%)	Contig N50	Scaffold N50	LAI*
Vigna angularis	98.8	38 kb	1.29 Mb	4.37
Vigna radiata	98.02	10.34 Mb	43.78 Mb	10.58
Vigna unguiculata	93.2	15.2 kb	2.7 Mb	10.23
Vigna subterranea	92.1	19.15 kb	640.67 kb	2.66
Phaseolus vulgaris	99.3	-	526.48 kb	2.95
Phaseolus acutifolius	95.8	47.04 kb	42.6 Mb	2.74
Phaseolus lunatus	93	-	3.99 Mb	0
Lablab purpureus	93.2	32.22 kb	621.37 kb	0
Glycine max	95.2	-	50.39 Mb	2.54
Glycine soja	99.8	3.33 Mb	50.69 Mb	3.87
Glycine latifolia	92.6	-	853.57 kb	1.81
Amphicarpaea edgeworthii	93.4	1.44 Mb	2.4 Mb	2.9
Pueraria montana	92.9	593.7 kb	-	2.11
Cajanus cajan	92.7	-	510 kb	4.18
Spatholobus suberectus	99.1	2.1 Mb	6.9 Mb	8.76
Abrus precatorius	98.8	11.84 Mb	35.86 Mb	10.36
Abrus melanospermus	97.1	18.93 Mb	18.95 Mb	10.51
Pongamia pinnata	96.78	-	88.49 Mb	12.4
Medicago polymorpha	91.8	11.02 Mb	57.72 Mb	4.78
Medicago truncatula	99.3	23.31 Mb	56.24 Mb	9.99
Medicago sativa	97.7	3.86 Mb	-	1.47
Medicago ruthenica	91.3	612.99 kb	99.39 Mb	12.78
Melilotus albus	96.67	7.49 Mb	-	4.61
Trifolium pratense	92.2	-	22.68 Mb	1.65
Trifolium subterranum	93.4	-	280 kb	0
Trifolium occidentale	94	-	191.74 kb	0
Trifolium pallescens	94	-	172.94 kb	5.6
Trifolium repens	92	-	121.66 kb	0
Pisum sativum	92.3	37.93 kb	415.94 kb	0
Vicia sativa	97.8	684.59 kb	290.13 Mb	2.64
Cicer arietinum	93.2	-	39.99 Mb	2.03
Oxytropis ochrocephala	93.26	1.39 Mb	121.79 Mb	0
Astragalus sinicus	91.1	1.50 Mb	78.42 Mb	7.19
Glycyrrhiza uralensis	90.32	7.32 kb	109.27 kb	2.15
Lotus japonicus	98.2	807 kb	-	11.98
Dalbergia odorifera	92.2	5.92 Mb	56.16 Mb	10.55
Aeschynomene evenia	94.4	-	985 kb	0
Arachis hypogaea	99.3	1.5 Mb	9 Mb	11.71
Arachis monticola	90	106.66 kb	124.92 Mb	5.98
Arachis duranensis	92.6	-	110.04 Mb	6.95
Arachis ipaensis	91.7	-	136.18 Mb	3.1

Nissolia schottii	95	21 kb	180 kb	0
Lupinus albus	95.2	1.76 Mb	18.66 Mb	0
Lupinus angustifolius	99.8	5.64 Mb	30.79 Mb	7.1
Ammopiptanthus nanus	92.15	2.76 Mb	-	5.71
Styphnolobium japonicum	98.1	17.34 Mb	31.32 Mb	10.88
Faidherbia albida	85.5	42.03 kb	692.04 kb	6.81
Mimosa pudica	94.3	11 kb	119.68 kb	0
Entada phaseoloides	98.8	6.34 Mb	30.9 Mb	6.07
Chamaecrista fasciculata	93.2	15 kb	96.64 kb	0
Senna tora	94.3	4.03 Mb	41.7 Mb	3.91
Cercis canadensis	95.7	13 kb	421.03 kb	4.18
Bauhinia variegata	98.7	4.54 Mb	22.1 Mb	11.41
Sindora glabra	90.9	1.27 Mb	84.87 Mb	10.08

*LTR-RT Assembly Index. This assessment of genome assembly quality was referred to Ou et al (Ou et al, 2018, Nucleic Acids Research).
